# Supplementary material for: Effectiveness of analgesic ear drops as add-on treatment to oral analgesics in children with acute otitis media: study protocol of the OPTIMA pragmatic randomised controlled trial
Source: BMJ Open. 2023 Feb 22;13(2):e062071. doi: 10.1136/bmjopen-2022-062071 (PMC9950909; doi:10.1136/bmjopen-2022-062071)
Supplement: Supplementary data [file bmjopen-2022-062071supp001.pdf]

Supplemental material 1. Methods and analyses mixed-method process evaluation

Surveys

Surveys will include brief statements which will be rated by parents on a 5-point scale (strongly disagree/ strongly agree). Parents of all trial participants will complete this survey at baseline and at 4 weeks (end of follow-up). Beliefs about the necessity and harms of antibiotics for AOM, oral analgesics and analgesic eardrops were assessed using questions based on the two key dimensions of treatment perceptions in the extended Common Sense Model: 'It is usually necessary to take antibiotics/oral analgesics/analgesic eardrops for an ear infection' and 'Taking antibiotics/oral analgesics/analgesic eardrops for an ear infection can do more harm than good'.[17] Patient satisfaction with the consultation will be assessed by a three-item Consultation Satisfaction Questionnaire that assessed whether they felt they had received all the information and advice they needed and were generally satisfied with the consultation.[18]

Semi-structured interviews

Semi-structured interviews will be carried out with parents and GPs participating in the trial. Interviews with parents will capture experiences of and satisfaction with care and subsequent views and experiences of management of symptoms (including eardrops and antibiotic use). Parents will be recruited from both the intervention and control arm and will be purposively sampled after study participation to obtain variation in age of child, symptom presentation, antibiotic prescribed, eardrops prescribed, gender, number of children, education level and employment status. Interviews will be carried out preferably within 2 weeks after study participation. Interviews with GPs will capture views and experiences of AOM consultations and the use of eardrops in these consultations. GPs will be purposively sampled to obtain variation in practice setting, gender, age and experience. Interviews with GPs will be carried out after the inclusion period. Data will be collected until saturation is reached; based on previous experience, we expect to conduct approximately 10 to 20 semi-structured interviews with both parents and GPs to reach saturation.[19 20]

Each interview will be audio recorded with the participant's permission. Recordings will allow verbatim transcription of interviews in Microsoft Word. Once transcripts are checked, recordings will be deleted. Transcripts will be labelled with a unique participant number and will omit any identifiable data either identifying the participant or their general practice.

Analysis of qualitative data

Qualitative data collection by means of semi-structured interviews and qualitative data analysis will be done concurrently. During the process, multiple members of the study team will review the transcripts of earlier interviews for accuracy and decide whether the topic lists need adaptation.

Interviews with parents and GPs, will be analysed using thematic and framework analysis taking an inductive approach.[21 22] NVivo software will be used to assist with the organisation of data. A thematic framework will be used to chart data across all interviews and will aid comparisons between participants.
