# Supplementary material for: Effectiveness of analgesic ear drops as add-on treatment to oral analgesics in children with acute otitis media: study protocol of the OPTIMA pragmatic randomised controlled trial
Source: BMJ Open. 2023 Feb 22;13(2):e062071. doi: 10.1136/bmjopen-2022-062071 (PMC9950909; doi:10.1136/bmjopen-2022-062071)
Supplement: Supplementary data [file bmjopen-2022-062071supp002.pdf]

Proefpersoneninformatie en toestemmingsformulier voor deelname aan medisch-wetenschappelijk onderzoek

OPTIMA: pijnstillende oordruppels bij acute middenoorontsteking

Officiële titel: Effectiviteit van pijnstillende oordruppels als toevoeging aan systemische pijnstillers bij kinderen met otitis media acuta: een pragmatische gerandomiseerde gecontroleerde studie

Inleiding

Geachte heer/mevrouw,

Met deze informatiebrief willen we u vragen of u met uw kind wilt meedoen aan medisch-wetenschappelijk onderzoek. Meedoen is vrijwillig. U krijgt deze brief omdat uw kind een acute middenoorontsteking heeft.
U leest hier om wat voor onderzoek het gaat, wat het voor u betekent, en wat de voordelen en nadelen zijn. Het is veel informatie. Wilt u de informatie doorlezen en beslissen of u wilt meedoen? Als u wilt meedoen, kunt u het formulier invullen dat u vindt in bijlage C.

Stel uw vragen

U kunt uw beslissing nemen met de informatie die u in deze informatiebrief vindt. Daarnaast raden we u aan om dit te doen:
- Stel vragen aan de onderzoeker die u deze informatie geeft.
- Praat met uw partner, familie of vrienden over dit onderzoek.
- Stel vragen aan de onafhankelijk deskundige, Dr. P.C.J.L. Bruijning-Verhagen (zie bijlage A voor contactgegevens).
- Lees de informatie op www.rijksoverheid.nl/mensenonderzoek.

1. Algemene informatie

Het Universitair Medisch Centrum (UMC) Utrecht heeft dit onderzoek opgezet. Voor dit onderzoek zijn 300 patiënten nodig. Dit onderzoek wordt gefinancierd door ZonMw. Deze organisatie financiert gezondheidsonderzoek in opdracht van het Ministerie van Volksgezondheid, Welzijn en Sport (VWS), en de Nederlandse Organisatie voor Wetenschappelijk Onderzoek (NWO).
De medisch-ethische toetsingscommissie Utrecht heeft dit onderzoek goedgekeurd.

de Sévaux JLH, *et al. BMJ Open* 2023; 13:e062071. doi: 10.1136/bmjopen-2022-062071

2. Wat is het doel van het onderzoek?

In dit onderzoek bekijken we wat het effect is van pijnstillende oordruppels bij kinderen met een acute middenoorontsteking, een ontsteking van de holte achter het trommelvlies.

3. Wat is de achtergrond van het onderzoek?

Een acute middenoorontsteking komt vaak voor op de kinderleeftijd. Het wordt veroorzaakt door een virus of een bacterie. Standaardzorg bestaat momenteel voornamelijk uit pijnstillende behandeling (paracetamol en zo nodig ibuprofen) en indien nodig antibiotica. Ondanks de inzet van deze standaardzorg, gaat de aandoening gepaard met oorpijn en ongemak bij kinderen en kan het een aanzienlijke impact op het gehele gezin hebben door slapeloze nachten en school- en werkverzuim. Het is daarom belangrijk dat er gezocht blijft worden naar andere behandelingen die de klachten kunnen verlichten. Eerdere studies lieten zien dat pijnstillende oordruppels mogelijk een gunstig effect hebben, maar het huidige bewijs is van onvoldoende kwaliteit om een zekere uitspraak over het effect te kunnen doen. Daarom willen we in deze studie onderzoeken of de pijnstillende oordruppels een betere verlichting van de oorpijn geven dan de gebruikelijke pijnstillers alleen. We gaan dit onderzoeken door 300 kinderen middels loting toe te wijzen aan 1) pijnstillende oordruppels bovenop de standaardzorg of 2) standaardzorg

4. Hoe verloopt het onderzoek?

Hoelang duurt het onderzoek?

Doet uw kind mee met het onderzoek? Dan duurt dat in totaal 4 weken.

Stap 1: is uw kind geschikt om mee te doen?

We willen eerst weten of uw kind geschikt is om mee te doen. Uw huisarts controleert of uw kind in aanmerking komt voor studiedeelname. Vervolgens geeft uw huisarts – met uw goedkeuring – uw gegevens door aan het onderzoeksteam in het UMC Utrecht en geeft u deze informatiefolder mee. Het onderzoeksteam neemt telefonisch contact met u op en één van de onderzoekers bezoekt u thuis, binnen 24 uur na uw bezoek aan de huisarts.. Aanvullend aan de beoordeling van de huisarts, zal de onderzoeker u enkele vragen stellen als extra controle op geschiktheid voor deelname.

Tijdens dit bezoek zal de onderzoeker u verder informeren over de studie en mogelijke vragen die u heeft beantwoorden. Pas daarna besluit u of u met uw kind wilt deelnemen aan het onderzoek. Indien u besluit om mee te doen vragen we aan beide ouders/voogden om een toestemmingsformulier te ondertekenen. De onderzoeker ondertekent dit formulier ook. Indien één van de ouders/voogden tijdens het eerste huisbezoek niet aanwezig is, zal van de betreffende ouder mondeling toestemming worden gevraagd. Alleen indien deze ook (mondelinge) toestemming geeft voor deelname, kan uw kind deelnemen aan het onderzoek. In het betreffende geval zal een extra proefpersoneninformatie en toestemmingsformulier bij u thuis worden achtergelaten om door de andere ouder te laten ondertekenen. Het getekende formulier zal op een ander moment opgehaald worden. In bijlage D staat

de Sévaux JLH, *et al. BMJ Open* 2023; 13:e062071. doi: 10.1136/bmjopen-2022-062071

bovenstaande procedure van informeren en toestemming geven (informed consent) op een schematische manier weergegeven.

Stap 2: de behandeling

Voor dit onderzoek verdelen we de deelnemende kinderen gelijk over 2 groepen:

- **Groep 1 (interventie groep)**. De kinderen in deze groep krijgen pijnstillende oordruppels (Otalgan® maximaal 6 keer/dag 1-2 druppels gedurende 7 dagen) bovenop de standaardzorg bestaande uit gebruikelijke pijnstillers en indien nodig naar inschatting van de huisarts antibiotica (50% van de kinderen).
  Let op: indien uw kind een loopoor krijgt dan moet u **direct stoppen** met het gebruik van de pijnstillende oordruppels in dat oor, neem in dat geval contact op met de onderzoeker.
- **Groep 2 (controle groep)**. De kinderen in deze groep krijgen de standaardzorg (50% van de kinderen).

Loting bepaalt in welke groep uw kind terecht zal komen.

Stap 3: verzamelen van gegevens

- Het **huisbezoek** op de dag van studiedeelname duurt circa één uur. Tijdens dit huisbezoek zal, nadat er toestemming gegeven is, een kort lichamelijk onderzoek worden verricht waarbij in de oren van uw kind gekeken wordt. Daarnaast neemt de onderzoeker de eerste vragenlijsten met u door. Aan het einde van het eerste huisbezoek zal de arts-onderzoeker via internet contact opnemen met het onderzoekscentrum in het UMC Utrecht om te zien wat het resultaat is van de loting die bepaalt welke behandeling uw kind krijgt. Dit geven wij door aan de huisarts en apotheek van uw kind. Indien uw kind in groep 1 ingedeeld wordt, ontvangt u aan het einde van het bezoek de oordruppels.
- We vragen u gedurende 4 weken om dagelijks een **klachtendagboekje** bij te houden. Dit kan online of op papier gedaan worden. U krijgt hier uitleg over tijdens het huisbezoek.
- Na **3 dagen** neemt een medewerker van het onderzoek **telefonisch contact** met u op om het dagboekje en eventuele vragen te bespreken. Dit duurt circa 10 minuten.
- Na **4 weken** neemt de onderzoeker nogmaals **telefonisch contact** met u op om de studie af te ronden en de laatste vragenlijsten met u door te nemen. Dit telefoon gesprek duurt maximaal 30 minuten.
- Optioneel kunt u ook nog deelnemen aan een telefonisch interview over uw ervaringen met de behandeling van de acute middenoorontsteking en de studie. Dit gebeurt alleen indien u hier toestemming voor geeft.

Wat is er anders dan bij gewone zorg?

Er is bij dit onderzoek niet zoveel anders dan bij gewone zorg. Indien uw kind in groep 1 geloot wordt, krijgt uw kind pijnstillende oordruppels bovenop de gewone zorg. Indien uw kind in groep 2 geplaatst wordt krijgt uw kind de gewone zorg.

5. Welke afspraken maken we met u?

We willen graag dat het onderzoek goed verloopt. Daarom maken we de volgende afspraken met u:

- U geeft het medicijn aan uw kind op de manier die de onderzoeker u heeft uitgelegd.
- Uw kind doet tijdens dit onderzoek niet ook nog mee aan een ander medisch-wetenschappelijk onderzoek.
- U vult de vragenlijsten en het dagboekje in zoals met u besproken is.
- U draagt de deelnemerskaart van het onderzoek bij u. Bijvoorbeeld in uw portemonnee. Hierop staat dat uw kind meedoet aan dit onderzoek. En wie men moet waarschuwen bij een noodsituatie. Laat deze kaart zien als uw kind bij een (andere) arts komt.
- Uw huisarts blijft ten alle tijden verantwoordelijk voor de medische behandeling van uw kind. Bij eventuele gezondheidsproblemen van uw kind neemt u contact op met uw huisarts zoals u normaal gesproken ook zou doen.
- U neemt contact op met de onderzoeker in deze specifieke situaties:
    - Uw kind krijgt een **loopoor**. In dit geval moet u **direct stoppen** met het gebruik van de pijnstillende oordruppels in dat oor.
    - Uw kind wordt in een ziekenhuis opgenomen of behandeld.
    - U en/of uw kind wilt niet meer meedoen met het onderzoek.
    - Uw telefoonnummer, adres of e-mailadres verandert.

6. Van welke bijwerkingen, nadelige effecten of ongemakken kunt u last krijgen?

Alle werkzame medicijnen kunnen bijwerkingen hebben. In de bijsluiter van de oordruppels staat dat er een kleine kans is op een overgevoeligheidsreactie. Deze bijwerking komt zelden voor (bij hooguit 1 op de 1.000 personen). We adviseren u dan om direct contact op te nemen met een (huis)arts.
In de bijsluiter van de oordruppels staat verder vermeld dat deze medicatie niet gebruikt dient te worden bij een loopoor of perforatie (gaatje/scheurtje) van het trommelvlies. Uw kind kan niet deelnemen aan het onderzoek als dit het geval is. Zowel de huisarts als de onderzoeker controleert dit aan het begin van het onderzoek door in de oren van uw kind te kijken.

Indien uw kind tijdens het onderzoek een **loopoor** krijgt mag uw kind de oordruppels **niet meer gebruiken**. Neem in dat geval contact op met de onderzoeker.

Doet u mee aan het onderzoek? Dan krijgt u de bijsluiter mee bij het middel.

7. Wat zijn de voordelen en de nadelen als u meedoet aan het onderzoek?

Meedoen aan het onderzoek kan voordelen en nadelen hebben. Hieronder zetten we ze op een rij. Denk hier goed over na, en praat erover met anderen.

Meedoen aan het onderzoek kan deze voordelen hebben:
De oordruppels kunnen mogelijk de mate van oorpijn van uw kind en het gebruik van antibiotica verminderen, maar zeker is dit niet.

Meedoen aan het onderzoek kan deze nadelen hebben:

- Uw kind kan last krijgen van bijwerkingen of nadelige effecten van de oordruppels, zoals beschreven in paragraaf 6.
- Meedoen aan het onderzoek kost u extra tijd.
- U moet zich houden aan de afspraken die horen bij het onderzoek.

Wilt u niet meedoen?
U beslist zelf of u met uw kind meedoet aan dit onderzoek. Deelname is geheel vrijwillig. Als u besluit niet mee te doen, hoeft u verder niets te doen. U hoeft niets te tekenen. U hoeft ook niet te zeggen waarom u niet wilt meedoen als u dat niet wilt. Uw kind krijgt dan gewoon de begeleiding en behandeling die hij/zij anders ook zou krijgen van uw huisarts.
Indien u wel meedoet, kunt u, of kan uw kind, zich op ieder moment van de studie bedenken en toch stoppen zonder de reden te melden. Dit zal geen gevolgen hebben voor de behandeling van uw kind.

8. Verzet van uw kind

Het kan gebeuren dat uw kind zich op een bepaald moment tijdens het onderzoek verzet (niet meewerkt). Indien u merkt dat dit het geval is, neem dan direct contact op met de onderzoeker. De onderzoeker moet het onderzoek dan direct stoppen. Het is moeilijk om precies te omschrijven wat verzet is. Voor de start van het onderzoek overleggen we met u wat wij zien als verzet.
De onderzoeker zal zich houden aan de Gedragscode ‘Verzet van minderjarigen die deelnemen aan medisch-wetenschappelijk onderzoek’ die door het NVK is vastgesteld.

9. Wanneer stopt het onderzoek?

De onderzoeker laat het u weten als er nieuwe informatie over het onderzoek komt die belangrijk voor u is. De onderzoeker vraagt u daarna of u blijft meedoen.

In deze situaties stopt voor u het onderzoek:

- Alle onderzoeken volgens het schema zijn voorbij.
- Het einde van het hele onderzoek is bereikt (na 4 weken)
- U of uw kind wilt zelf stoppen met het onderzoek. Dat mag op ieder moment. Meld dit dan meteen bij de onderzoeker. U hoeft er niet bij te vertellen waarom u stopt. Uw kind krijgt dan weer de gewone behandeling voor de middenoorontsteking. De onderzoeker zal u mogelijk nog wel uitnodigen voor een nagesprek.

de Sévaux JLH, *et al. BMJ Open* 2023; 13:e062071. doi: 10.1136/bmjopen-2022-062071

Optima
pijnstillende oordruppels bij otitis media acuta

UMC Utrecht

- De onderzoeker vindt het beter voor u om te stoppen. De onderzoeker zal u mogelijk nog wel uitnodigen voor een nagesprek.
- Een van de volgende instanties besluit dat het onderzoek moet stoppen:
    - UMC Utrecht
    - de overheid, of
    - de medisch-ethische commissie die het onderzoek beoordeelt.

Wat gebeurt er als u stopt met het onderzoek?
De onderzoekers gebruiken de gegevens die tot het moment van stoppen zijn verzameld.

Het hele onderzoek is afgelopen als alle deelnemers klaar zijn.

10. Wat gebeurt er na het onderzoek?

Krijgt u de resultaten van het onderzoek?
Na afloop van de hele studie, als de gegevens van alle 300 kinderen zijn verzameld en verwerkt, ontvangt u van ons bericht over de belangrijkste uitkomsten van het onderzoek.

11. Wat doen we met de gegevens van uw kind?

Doet u mee met het onderzoek? Dan geeft u ook toestemming om de gegevens van uw kind te verzamelen, gebruiken en bewaren.

Welke gegevens bewaren we?
We bewaren deze gegevens:
- naam, geslacht en geboortedatum van uw kind
- adres, telefoonnummer en emailadres
- gegevens over gezondheid van uw kind
- (medische) gegevens die we tijdens het onderzoek verzamelen

Waarom verzamelen, gebruiken en bewaren we de gegevens van uw kind?
We verzamelen, gebruiken en bewaren de gegevens van uw kind om de vragen van dit onderzoek te kunnen beantwoorden. En om de resultaten te kunnen publiceren.

Hoe beschermen we de privacy van u en uw kind?
Om de privacy te beschermen geven wij de gegevens een code. Op de gegevens zetten we alleen deze code. De sleutel van de code bewaren we op een beveiligde plek in het UMC Utrecht. Als we de gegevens van uw kind verwerken, gebruiken we steeds alleen die code. Ook in rapporten en publicaties over het onderzoek kan niemand terughalen dat het over uw kind ging.

Wie kunnen de gegevens van uw kind zien?

Sommige personen kunnen wel de naam en andere persoonlijke gegevens van uw kind zonder code inzien. Dit zijn mensen die controleren of de onderzoekers het onderzoek goed en betrouwbaar uitvoeren. Deze personen kunnen bij de gegevens van uw kind komen:

- Een controleur die voor het UMC Utrecht werkt.
- Nationale toezichthoudende autoriteiten. Bijvoorbeeld de Inspectie Gezondheidszorg en Jeugd.

Deze personen houden de gegevens van uw kind geheim. Wij vragen u voor deze inzage toestemming te geven.

Hoelang bewaren we de gegevens van uw kind?
We bewaren de gegevens van uw kind 25 jaar.

Mogen we de gegevens van uw kind gebruiken voor ander onderzoek?
De gegevens van uw kind kunnen na afloop van dit onderzoek ook nog van belang zijn voor ander wetenschappelijk onderzoek op het gebied van acute middenoorontsteking. Daarvoor zullen de gegevens van uw kind 25 jaar worden bewaard in het onderzoekscentrum. In het toestemmingformulier geeft u aan of u dit goed vindt. Geeft u geen toestemming? Dan kunt u nog steeds meedoen met dit onderzoek. Uw kind krijgt dezelfde zorg.

Kunt u uw toestemming voor het gebruik van de gegevens van uw kind weer intrekken?
U kunt uw toestemming voor het gebruik van de gegevens van uw kind op ieder moment intrekken. Dit geldt voor het gebruik in dit onderzoek en voor het gebruik in ander onderzoek. Maar let op: trekt u uw toestemming in, en hebben onderzoekers dan al gegevens verzameld voor een onderzoek? Dan mogen zij deze gegevens nog wel gebruiken.

Mogelijke sturen we de gegevens van uw kind gecodeerd naar landen buiten de Europese Unie
In dit onderzoek kan het zijn dat we de gecodeerde gegevens van uw kind ook naar landen buiten de Europese Unie sturen. In die landen gelden niet de privacyregels van de Europese Unie. Maar de privacy van uw kind zal op een gelijkwaardig niveau worden beschermd.

Wilt u meer weten over de privacy van uw kind?

- Wilt u meer weten over de rechten van uw kind bij de verwerking van persoonsgegevens? Kijk dan op www.autoriteitpersoonsgegevens.nl.
- Heeft u vragen over de rechten van uw kind? Of heeft u een klacht over de verwerking van de persoonsgegevens van uw kind? Neem dan contact op met degene die verantwoordelijk is voor de verwerking van de persoonsgegevens van uw kind. Voor uw onderzoek is dat:
  - Het UMC Utrecht. Zie bijlage A voor contactgegevens, en website.
- Als u klachten heeft over de verwerking van de persoonsgegevens van uw kind, raden we u aan om deze eerst te bespreken met het onderzoeksteam. U kunt ook naar de

de Sévaux JLH, *et al. BMJ Open* 2023; 13:e062071. doi: 10.1136/bmjopen-2022-062071

Functionaris Gegevensbescherming van het UMC Utrecht gaan. Of u dient een klacht in bij de Autoriteit Persoonsgegevens.

Waar vindt u meer informatie over het onderzoek?
Op de volgende website vindt u meer informatie over het onderzoek: https://www.trialregister.nl/trial/9500. Na het onderzoek kan de website een samenvatting van de resultaten van dit onderzoek tonen. U vindt het onderzoek door te zoeken op ‘NL9500’.

12. Krijgt u een vergoeding als uw kind meedoet aan het onderzoek?

Aan het onderzoek zijn voor u geen kosten verbonden. Er is geen vergoeding beschikbaar voor deelname, na afloop van het huisbezoek krijgt uw kind wel een klein cadeautje.

13. Bent u verzekerd tijdens het onderzoek?

Voor iedereen die meedoet aan dit onderzoek is een verzekering afgesloten. De verzekering betaalt voor schade door het onderzoek. Maar niet voor alle schade. In **bijlage B** vindt u meer informatie over de verzekering en de uitzonderingen. Daar staat ook aan wie u schade kunt melden.

14. We informeren de huisarts en apotheker van uw kind

De onderzoeker stuurt de huisarts en apotheker van uw kind een bericht om te laten weten dat u meedoet aan het onderzoek. Daarnaast vragen we de apotheek naar bekende allergieën. Dit is voor de eigen veiligheid van uw kind.

15. Heeft u vragen?

Vragen over het onderzoek kunt u stellen aan het onderzoeksteam. Wilt u advies van iemand die er geen belang bij heeft? Dan kunt u terecht bij een onafhankelijke deskundige. Voor ons onderzoek is dat dr. P.C.J.L. Bruijning-Verhagen (zie bijlage A voor contactgegevens). Zij weet veel over het onderzoek, maar werkt niet mee aan dit onderzoek.
Heeft u een klacht? Bespreek dit dan met de onderzoeker of de arts die u behandelt. Wilt u dit liever niet? Ga dan naar Klachtenbemiddeling van het UMC Utrecht. In bijlage A staat waar u die kunt vinden.

16. Hoe geeft u toestemming voor het onderzoek?

U kunt eerst rustig nadenken over dit onderzoek. Daarna vertelt u de onderzoeker of u de informatie begrijpt en of u wel of niet wilt meedoen met uw kind. Wilt u met uw kind meedoen? Dan vult u het toestemmingsformulier in dat u bij deze informatiebrief vindt. U en de onderzoeker krijgen allebei een getekende versie van deze toestemmingsverklaring.

Dank voor uw tijd.

de Sévaux JLH, *et al. BMJ Open* 2023; 13:e062071. doi: 10.1136/bmjopen-2022-062071

Met vriendelijke groet,

Drs. Joline de Sévaux, arts-onderzoeker, mede namens OPTIMA onderzoeksteam:

Dr. Roderick P Venekamp, huisarts

Professor dr. Roger AMJ Damoiseaux, huisarts

Professor dr. Anne GM Schilder, KNO-arts

Drs. Saskia Hullegie, arts-onderzoeker

17. Bijlagen bij deze informatie

A. Contactgegevens
B. Informatie over de verzekering
C. Toestemmingsformulier
D. Procedure informeren en toestemming geven (informed consent)

de Sévaux JLH, *et al. BMJ Open* 2023; 13:e062071. doi: 10.1136/bmjopen-2022-062071

Bijlage A: contactgegevens voor UMC Utrecht

<u>Contactpersoon onderzoeksteam:</u>
Naam: J.L.H. de Sévaux, arts-onderzoeker
Julius Centrum voor Gezondheidswetenschappen en Eerstelijnsgeneeskunde, Afdeling Huisartsgeneeskunde
Universitair Medisch Centrum Utrecht
e-mail: optima@umcutrecht.nl
tel. nummer: 0625710099
Bereikbaar: dagelijks tijdens kantooruren.

<u>Onafhankelijk deskundige:</u>
Naam: Dr. P.C.J.L. Bruijning-Verhagen, kinderarts
UMC Utrecht
email: p.bruijning@umcutrecht.nl
tel. nummer: 088 75 681 81

<u>Klachten:</u>
Als u klachten heeft kunt u dit melden aan de onderzoeker of aan uw behandelend arts. Mocht u ontevreden zijn over de gang van zaken bij het onderzoek en een klacht willen indienen dan kunt u contact opnemen met de klachtenbemiddelaars. Deze zijn bereikbaar via tel. +31 88 755 62 08. Of digitaal via: https://www.umcutrecht.nl/nl/een-klacht-indienen

Functionaris voor de Gegevensbescherming van de instelling: privacy@umcutrecht.nl

Raadpleeg de website van het UMC Utrecht voor meer informatie over uw rechten:
https://www.umcutrecht.nl/nl/ziekenhuis/privacy

de Sévaux JLH, et al. BMJ Open 2023; 13:e062071. doi: 10.1136/bmjopen-2022-062071

Optima
pijnstillende oordruppels bij otitis media acuta

UMC Utrecht

Bijlage B: informatie over de verzekering

UMC Utrecht heeft een verzekering afgesloten voor iedereen die meedoet aan het onderzoek. De verzekering betaalt de schade die u heeft doordat u aan het onderzoek meedeed. Het gaat om schade die u krijgt tijdens het onderzoek, of binnen 4 jaar na het onderzoek. U moet schade binnen 4 jaar melden bij de verzekeraar.

Heeft u schade door het onderzoek? Meld dit dan telefonisch of per post bij deze verzekeraar:

De verzekeraar van het onderzoek is:	
Naam:	CNA Insurance Company Ltd
Adres:	Polarisavenue 140, 2132 JX Hoofddorp
Telefoonnummer:	020 57 37 274
Polisnummer:	10201366
Contactpersoon:	Mw. Esther van Herk

De verzekering betaalt maximaal € 650.000 per persoon en € 5.000.000 voor het hele onderzoek en € 7.500.000 per jaar voor alle onderzoeken van dezelfde opdrachtgever (het UMC Utrecht).

Let op: de verzekering dekt de volgende schade **niet**:

- Schade door een risico waarover we u informatie hebben gegeven in deze brief. Maar dit geldt niet als het risico groter bleek te zijn dan we van tevoren dachten. Of als het risico heel onwaarschijnlijk was.
- Schade aan uw gezondheid die ook zou zijn ontstaan als u niet aan het onderzoek had meegedaan.
- Schade die ontstaat doordat u aanwijzingen of instructies niet of niet goed opvolgde.
- Schade aan de gezondheid van uw kinderen of kleinkinderen.
- Schade door een behandelmethode die al bestaat. Of door onderzoek naar een behandelmethode die al bestaat.

Deze bepalingen staan in het 'Besluit verplichte verzekering bij medisch-wetenschappelijk onderzoek met mensen 2015'. Dit besluit staat in de Wettenbank van de overheid (https://wetten.overheid.nl).

de Sévaux JLH, *et al. BMJ Open* 2023; 13:e062071. doi: 10.1136/bmjopen-2022-062071

Bijlage C: Toestemmingsformulier ouders of voogd

Behorende bij de **OPTIMA-studie (pijnstillende oordruppels bij acute middenoorontsteking)**

Ik ben gevraagd om toestemming te geven voor deelname van mijn kind aan dit medisch-wetenschappelijke onderzoek:

Naam proefpersoon (kind): …………………….. Geboortedatum: ___ / ___ / _____

- Ik heb de informatiebrief voor de ouders/verzorgers gelezen. Ook kon ik vragen stellen. Mijn vragen zijn goed genoeg beantwoord. Ik had genoeg tijd om te beslissen of ik wil dat mijn kind meedoet.
- Ik weet dat meedoen vrijwillig is. Ook weet ik dat ik op ieder moment kan beslissen dat mijn kind toch niet meedoet. Ik hoef dan niet te zeggen waarom ik dat wil.
- Ik geef de onderzoeker toestemming om mijn huisarts en apotheker te laten weten dat mijn kind meedoet aan dit onderzoek.
- Ik geef de onderzoekers toestemming om de gegevens van mijn kind te verzamelen en te gebruiken. De onderzoekers doen dit alleen om de onderzoeksvraag in dit onderzoek te beantwoorden.
- Ik weet dat voor de controle van het onderzoek sommige mensen toegang tot alle gegevens van mijn kind kunnen krijgen. Die mensen staan in deze informatiebrief. Ik geef deze mensen toestemming om de gegevens van mijn kind in te zien voor deze controle.
- Ik weet dat mijn gecodeerde gegevens naar landen buiten de EU kunnen worden gestuurd waar privacyregels van de EU niet gelden.
- Wilt u in de tabel hieronder ja of nee aankruisen?

Ik geef toestemming om mij na dit onderzoek te benaderen voor een interview over uw ervaringen met betrekking tot de studiedeelname en de behandeling.	Ja ☐	Nee☐
Ik geef toestemming om mijn kind na dit onderzoek te vragen of hij/zij wil meedoen met een vervolgonderzoek.	Ja ☐	Nee☐
Ik geef toestemming om de gegevens van mijn kind te bewaren om dit te gebruiken voor ander onderzoek, zoals in de informatiebrief staat.	Ja ☐	Nee☐

- Ik ga ermee akkoord dat mijn kind meedoet aan dit onderzoek.

de Sévaux JLH, et al. BMJ Open 2023; 13:e062071. doi: 10.1136/bmjopen-2022-062071

Naam ouder/voogd**: ………………………………

Handtekening: ………………………………… Datum: ___ / ___ / _____

Naam andere ouder/voogd**: ……………………

Handtekening: ……………………………………… Datum: ___ / ___ / _____

Ik verklaar dat ik de persoon/personen hierboven volledig heb geïnformeerd over het genoemde onderzoek.

Wordt er tijdens het onderzoek informatie bekend die de toestemming van de ouder of voogd kan beïnvloeden? Dan laat ik dit op tijd aan hem/haar weten.

Naam onderzoeker (of diens vertegenwoordiger): …………………………

Handtekening: ……………………… Datum: ___ / ___ / _____

----

* Doorhalen wat niet van toepassing is.

** Als het kind jonger dan 16 jaar is, ondertekenen de ouders die het gezag uitoefenen of de voogd dit formulier. Kinderen van 12 t/m 15 jaar die zelfstandig beslissingen kunnen nemen (wilsbekwaam zijn), moeten daarnaast zelf een formulier ondertekenen

De ouder/voogd krijgt een volledige informatiebrief mee, samen met een getekende versie van het toestemmingsformulier.

de Sévaux JLH, *et al. BMJ Open* 2023; 13:e062071. doi: 10.1136/bmjopen-2022-062071

Optima
pijnstillende oordruppels bij otitis media acuta

UMC Utrecht

Bijlage D: Procedure informeren en toestemming geven (informed consent)

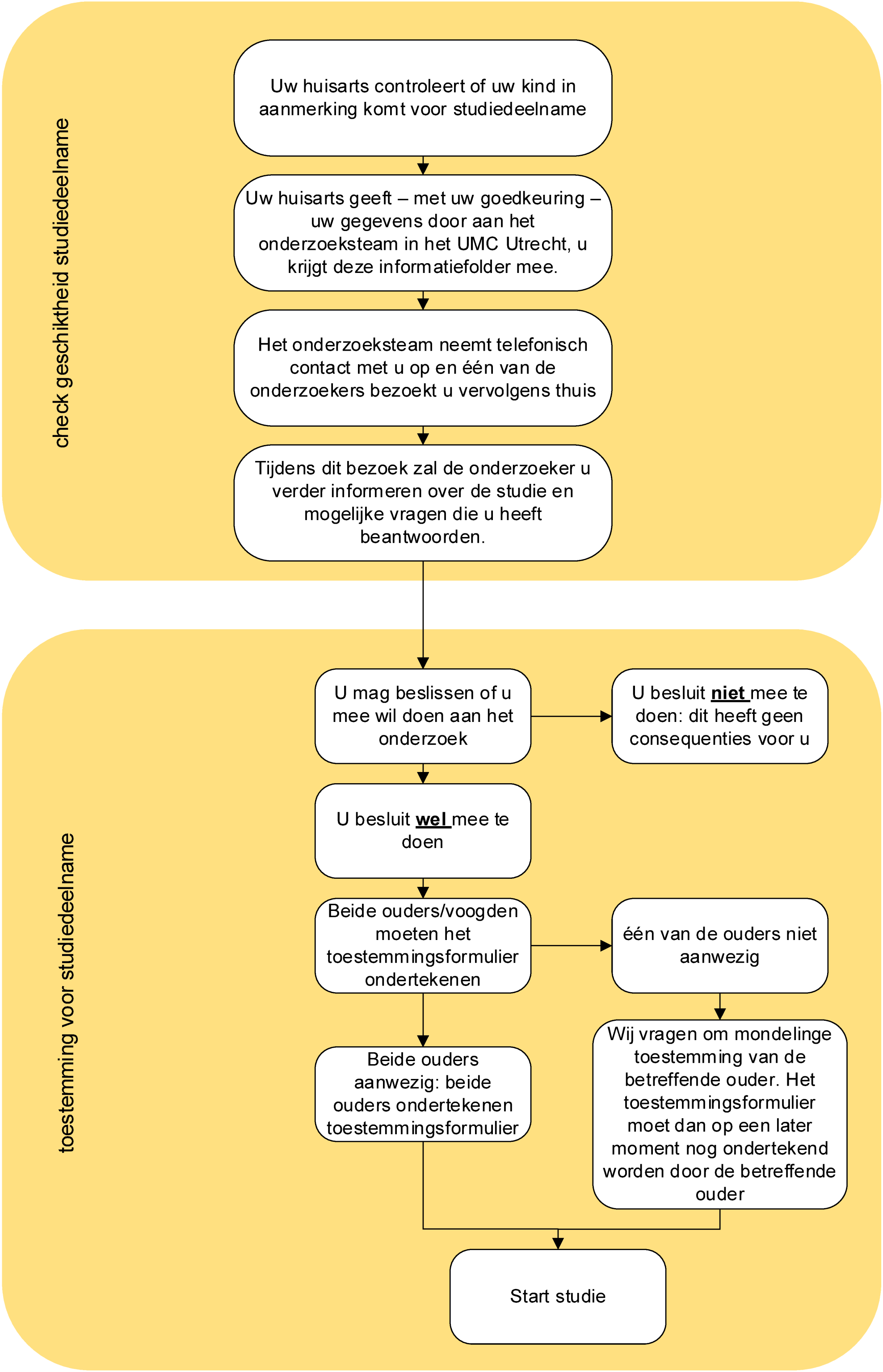

de Sévaux JLH, *et al. BMJ Open* 2023; 13:e062071. doi: 10.1136/bmjopen-2022-062071
