# Supplementary material for: Effectiveness of analgesic ear drops as add-on treatment to oral analgesics in children with acute otitis media: study protocol of the OPTIMA pragmatic randomised controlled trial
Source: BMJ Open. 2023 Feb 22;13(2):e062071. doi: 10.1136/bmjopen-2022-062071 (PMC9950909; doi:10.1136/bmjopen-2022-062071)
Supplement: Author's manuscript [file bmjopen-2022-062071.draft_revisions.pdf]

BMJ Open is committed to open peer review. As part of this commitment we make the peer review history of every article we publish publicly available.

When an article is published we post the peer reviewers' comments and the authors' responses online. We also post the versions of the paper that were used during peer review. These are the versions that the peer review comments apply to.

The versions of the paper that follow are the versions that were submitted during the peer review process. They are not the versions of record or the final published versions. They should not be cited or distributed as the published version of this manuscript.

BMJ Open is an open access journal and the full, final, typeset and author-corrected version of record of the manuscript is available on our site with no access controls, subscription charges or pay-per-view fees (http://bmjopen.bmj.com).

If you have any questions on BMJ Open's open peer review process please email info.bmjopen@bmj.com

BMJ Open

Effectiveness of analgesic ear drops as add-on treatment to oral analgesics in children with acute otitis media: study protocol of the OPTIMA pragmatic randomised controlled trial

Journal:	*BMJ Open*
Manuscript ID	bmjopen-2022-062071
Article Type:	Protocol
Date Submitted by the Author:	18-Feb-2022

Complete List of Authors:	de Sévaux, Joline; University Medical Centre Utrecht, Julius Center for Health Sciences and Primary Care<br>Damoiseaux, Roger; University Medical Centre Utrecht, Julius Center for Health Sciences and Primary Care<br>Hullegie, Saskia; University Medical Centre Utrecht, Julius Center for Health Sciences and Primary Care<br>Sanders, Elisabeth ; Wilhelmina Children's Hospital University Medical Centre, Department of Paediatric Immunology and Infectious Diseases; National Institute for Public Health and the Environment, Centre for Infectious Disease Control<br>Wit, Ardine; University Medical Centre Utrecht, Julius Center for Health Sciences and Primary Care; National Institute for Public Health and the Environment, Centre for Nutrition, Prevention and Healthcare<br>Zuithoff, Nicolaas; University Medical Centre Utrecht, Julius Center for Health Sciences and Primary Care<br>Yardley, Lucy; University of Southampton, School of Psychology, Faculty of Environmental and Life Sciences; University of Bristol, School of Psychological Science, Faculty of Life Sciences<br>Anthierens , Sibyl ; University of Antwerp Faculty of Medicine and Health Sciences, Department of Family Medicine and Population Health<br>Little, Paul; University of Southampton, Primary Care Population Sciences and Medical Education, Aldermoor Health Centre<br>Hay, Alastair; University of Bristol, Centre for Academic Primary Care, School of Social and Community Medicine<br>Schilder, Anne; University Medical Centre Utrecht, Julius Center for Health Sciences and Primary Care; NIHR University College London Hospitals Biomedical Research Centre<br>Venekamp , Roderick ; University Medical Centre Utrecht, Julius Center for Health Sciences and Primary Care

Keywords:	PRIMARY CARE, Epidemiology < INFECTIOUS DISEASES, Paediatric otolaryngology < OTOLARYNGOLOGY, Paediatric infectious disease & immunisation < PAEDIATRICS, PAIN MANAGEMENT, QUALITATIVE RESEARCH

Effectiveness of analgesic ear drops as add-on treatment to oral analgesics in children with acute otitis media: study protocol of the OPTIMA pragmatic randomised controlled trial

Joline LH de Sévaux[1], Roger AMJ Damoiseaux[1], Saskia Hullegie[1], Elisabeth AM Sanders[2,3], G Ardine de Wit[1,4], Nicolaas P A Zuithoff[1], Lucy Yardley[5,6], Sibyl Anthierens[7], Paul Little[8], Alastair D Hay[9], Anne GM Schilder[1,10,11], Roderick P Venekamp[1]

[1] Julius Center for Health Sciences and Primary Care, University Medical Center Utrecht, Utrecht University, Utrecht, The Netherlands
[2] Department of Paediatric Immunology and Infectious Diseases, Wilhelmina Children's Hospital, University Medical Center Utrecht, Utrecht University, Utrecht, The Netherlands
[3] Centre for Infectious Disease Control, National Institute of Public Health and the Environment (RIVM), Bilthoven, The Netherlands
[4] Centre for Nutrition, Prevention and Healthcare, National Institute of Public Health and the Environment (RIVM), Bilthoven, the Netherlands
[5]School of Psychological Science, Faculty of Life Sciences, University of Bristol, Bristol, United Kingdom
[6] School of Psychology, Faculty of Environmental and Life Sciences, University of Southampton, Southampton, United Kingdom
[7] Department of Family Medicine and Population Health, Faculty of Medicine and Health Sciences, University of Antwerp, Antwerp, Belgium
[8] Primary Care Population Sciences and Medical Education, Aldermoor Health Centre, University of Southampton, Southampton, United Kingdom
[9] Centre for Academic Primary Care, School of Social and Community Medicine, University of Bristol, Bristol, United Kingdom
[10] National Institute for Health Research University College London Hospitals Biomedical Research Centre
[11] evidENT, Ear Institute, University College London, London, United Kingdom

Corresponding author

Joline LH de Sévaux
Julius Center for Health Sciences and Primary Care
University Medical Center Utrecht
Str. 6.131, P.O. Box 85500, 3508 GA Utrecht, the Netherlands
Tel: +31 6 25 71 00 99
Email: j.l.h.desevaux-2@umcutrecht.nl

For peer review only

For peer review only - http://bmjopen.bmj.com/site/about/guidelines.xhtml

ABSTRACT

Introduction

Ear pain is the most prominent symptom of childhood acute otitis media (AOM). To control the pain and reduce reliance on antibiotics, evidence of effectiveness for alternative interventions is urgently needed. This trial aims to investigate whether analgesic ear drops added to usual care provide superior ear pain relief over usual care alone in children presenting to primary care with AOM.

Methods and analysis

This is a pragmatic, two arm, individually randomised, open, superiority trial with cost-effectiveness analysis and nested mixed-methods process evaluation in general practices in the Netherlands. We aim to recruit 300 children aged 1-6 years with a general practitioner (GP)-diagnosis of AOM and ear pain. Children will be randomly allocated to either 1) lidocaine hydrochloride 5mg/g ear drops (Otalgan) 1-2 drops up to six times daily for a maximum of 7 days in addition to usual care (oral analgesics, with/without antibiotics) or 2) usual care. Parents will complete a symptom diary for 4 weeks as well as generic and disease-specific quality of life questionnaires at baseline and 4 weeks. The primary outcome is the mean parent-reported ear pain score (0-10) over the first 3 days. Secondary outcomes include proportion of children consuming antibiotics, oral analgesic use and overall symptom burden in the first 7 days; number of days with ear pain, number of GP reconsultations and subsequent antibiotic prescribing, adverse events, complications of AOM and cost-effectiveness during 4-week follow-up; generic and disease-specific quality of life at 4 weeks; parents' and GPs' views and experiences with treatment acceptability, usability and satisfaction.

Ethics and dissemination

The Medical Research Ethics Committee Utrecht, the Netherlands, has approved the protocol (21-447/G-D). All parents/guardians of participants will provide written informed consent. Study results will be submitted for publication in peer reviewed medical journals and presented at relevant (inter)national scientific meetings.

Trial registration: The Netherlands Trial Register; NL9500. Date of registration: 28 May 2021.

Word count: 3519 (excluding title page, abstract, references, figures and boxes).

Keywords: acute otitis media; children, primary care; analgesic ear drops; randomised controlled trial.

For peer review only - http://bmjopen.bmj.com/site/about/guidelines.xhtml

ARTICLE SUMMARY

Strengths

- The pragmatic, open design enhances the applicability of the trial findings in daily practice.
- The pragmatic, open design is most suited to address the key secondary outcomes of the impact of the analgesic ear drops on the proportion of children consuming antibiotics and its cost-effectiveness in everyday practice.

Limitations

- The combination of the open design and the subjective, parent-reported outcomes does not control for the placebo effects, but will more closely reflect the benefits that would accrue in everyday practice with regard to analgesic effects.
- Contamination is possible given the over-the-counter availability of analgesic eardrops.

For peer review only

INTRODUCTION

Although the current COVID-19 pandemic has a profound influence on the incidence of acute otitis media (AOM) in children[1-3], it will remain an important cause of primary care consultations.[4 5] Ear pain is the predominant symptom and central to children's and parents' experience of the condition.[6 7] High-quality studies have shown that most symptoms of AOM settle within a few days without antibiotics in otherwise healthy children living in high-income countries[8], but it may take eight days for ear pain to fully resolve in 90% of the children.[9] Globally, most AOM clinical practice guidelines therefore recommend oral analgesics in all, and antibiotics in selected, children.[10] However, antibiotics are frequently prescribed in children with AOM[4 5] leading to side effects in at least 1 out of 10 children and emergence of antimicrobial resistance.[11 12] Evidence of effectiveness of management options to relieve the most distressing symptom of AOM and reduce reliance on antibiotics is therefore urgently needed.[13]

Recently, we found that a general practitioner (GP)-targeted educational intervention to improve pain management in children with AOM resulted in an increase in oral analgesic use, in particular ibuprofen, but failed to reduce parent-reported ear pain and antibiotic use.[14] Therefore, alternative interventions need further exploration. Analgesic ear drops, widely available, cheap and safe, may be well-suited to fill this gap. When applied into the external auditory canal, these agents could relieve pain and distress by blocking nerve conduction of the tympanic membrane.[15] So far, evidence on their use in children with AOM is limited.[15 16] Our trial aims to produce this important evidence.

OBJECTIVE

The primary aim of this pragmatic randomised controlled trial is to investigate whether analgesic ear drops added to usual care (oral analgesics with/without antibiotics) provide superior ear pain relief over usual care alone in children presenting to primary care with AOM and ear pain.

For peer review only - http://bmjopen.bmj.com/site/about/guidelines.xhtml

METHODS AND ANALYSIS

Study design and setting

A pragmatic, two arm, individually randomised, open, superiority trial with cost-effectiveness analysis in general practices in the Netherlands with a follow-up of 4 weeks will be performed. Potential trial participants will be recruited from primary care (approximately from 150 GPs) in the region of Utrecht, The Netherlands. A mixed-method process evaluation will be nested within the trial (see Supplemental Material 1).[17-22] This process evaluation will capture data to understand how participating in the trial has an impact on the consultation and behaviour and how AOM interventions (including analgesic ear drops, antibiotics and oral analgesics) are viewed by parents and GPs. The process evaluation consists of a survey with brief statements measuring attitudes of all parents based on the extended Common Sense Model[17] and a three-item Consultation Satisfaction Questionnaire,[18] and a qualitative part (semi-structured interviews capturing parents and GP's views and experiences, respectively).

Participants

Children aged 1-6 years presenting to primary care with AOM[23] and parent-reported ear pain in 24 hours prior to enrolment will be eligible for trial participation. Children with (suspected) tympanic membrane perforation or ventilation tube (as they carry a risk of inner ear damage causing hearing loss or tinnitus after application of lidocaine drops), ear wax obscuring visualisation of the tympanic membrane or those who are very unwell or require hospitalisation will be excluded. Detailed inclusion and exclusion criteria are listed in Box 1.

For peer review only

Box 1. Full list of inclusion and exclusion criteria

Inclusion criteria:
- Age 1 to 6 years
- Parent-reported ear pain in 24 hours prior to enrolment
- GP-diagnosis of (uni- or bilateral) AOM

Exclusion criteria:
Children:
- with (suspected) tympanic membrane perforation or ventilation tubes
- with ear wax obscuring visualisation of the tympanic membrane
- who are systemically very unwell or require hospital admission (e.g. child has signs and symptoms of serious illness and/or complications such as mastoiditis/meningitis)
- who are at high risk of serious complications including children with known immunodeficiency other than partial IgA or IgG2 deficiencies, craniofacial malformation including cleft palate, Down syndrome and previous ear surgery (with the exception of ventilation tubes in the past)
- who have a known allergy or sensitivity to study medication or similar substances (e.g. other amide-type anaesthetics: bupivacaine, mepivacaine, prilocaine, etc)
- who have taken part in any research involving medicines within the last 90 days, or any other AOM-related research within the last 30 days
- who suffer from chronic recurrent pain of another origin than the ear
- who have participated in this trial during prior AOM episode

Study procedures and data collection

Recruitment and inclusion of participants

Figure 1 depicts a flow diagram of the study procedures. During regular primary care consultations, participating GPs will inform parents of potentially eligible children about the trial. Next, GPs will ask parents who are interested in participation for consent to provide their contact details to the study team at the University Medical Center (UMC) Utrecht. Members of the team will contact the parents by phone the same day to provide detailed information about the trial. If parents provisionally agree to participate and if the child fulfils the trial eligibility criteria, the study physician will visit the child at home. At this home visit, written informed consent for participation in the trial and the nested process evaluation will be obtained (Supplemental Material 2), baseline demographic and disease-specific data will be collected and a physical examination including otoscopy will be performed. Parents are also asked to complete a generic and disease-

For peer review only - http://bmjopen.bmj.com/site/about/guidelines.xhtml

specific quality of life questionnaires on behalf of their child. A full description of the nested process evaluation is given in Supplemental Material 1

Study group assignment

At the conclusion of the home visit, a trial randomisation website will be accessed for concealed study-group assignment. An independent data manager generates a computer-generated randomisation sequence stratified for age (<2 years vs 2 years and above), AOM laterality (unilateral vs bilateral), and AOM baseline antibiotic prescribing (yes vs no). Children will be randomly allocated to either 1) lidocaine hydrochloride 5mg/g (Otalgan) ear drops 1-2 drops up to six times daily for a maximum of 7 days[24 25] with usual care (oral analgesics, with/without antibiotics) or 2) usual care. Analgesic eardrops will be provided by the study team.

The study team will notify the GP and local pharmacist about the result of the randomisation. Any treatment decisions other than the use of analgesic ear drops, i.e. antibiotics and oral analgesics, will be left to the GP's discretion in both groups. To those allocated to the intervention group, the study physician will not provide any treatment advice other than instructions about the use of analgesic ear drops.

During follow-up parents and GPs will be encouraged to manage AOM symptoms according to current clinical practice guidance on AOM in children issued by the Dutch College of General Practitioners.[23]

Follow-up data collection

Participants will be followed for 4 weeks. A telephone call will be scheduled at day 3 which will allow us to capture critical data on our primary outcome. Parents will keep a diary of AOM-related symptoms including ear pain scores and fever recordings, treatment adherence, prescribed and over-the-counter (OTC) medication use, AOM-related health care resource use, child care and travel costs, adverse events and complications of AOM for 4 weeks. At the end of follow-up, parents will complete a questionnaire on parental productivity losses and generic and disease-specific quality of life questionnaires. The study team will contact the parents by phone to check if all data is collected. During this phone call, an appointment for the semi-structured interview can be made with parents who are still willing to participate in the nested qualitative investigation.

Validated questionnaires used in the trial

- Child's ear pain intensity will be reported by parents in the symptom diary using a 0-10 numerical rating scale

For peer review only - http://bmjopen.bmj.com/site/about/guidelines.xhtml

which has been validated for measuring pain in children with AOM.[26]

- Child's symptoms of crying/distress, disturbed sleep, interference with normal activity, appetite, fever and hearing problems will be reported by parents in the symptom diary using a 0-6 Likert scale with higher scores indicating greater severity. This scale has been proven valid and was successfully used in our previous trials.[16 26]

- Generic quality of life of the child at baseline and 4 weeks will be assessed using the 47-item short-form of the Infant Toddler Quality of Life Questionnaire (ITQOL-SF47) which is developed for use in infants and toddlers aged 2 months to 6 years and has shown to be a feasible instrument with adequate psychometric properties to discriminates between children with and without doctor-diagnosed respiratory disease.[27]

- Disease-specific quality of life of the child at baseline and 4 weeks will be assessed using the Otitis media-6 (OM-6) questionnaire.[28]

- Productivity losses (e.g. parental absenteeism from work) because of AOM of their child will be assessed using the IMTA Productivity Cost Questionnaire (iPCQ) at 4 weeks.[29]

Outcomes

Primary outcome

Mean parent-reported ear pain score over the first three days. To this end, parents will record their child's ear pain scores during the first three consecutive days using a 0-10 validated numerical rating scale.

Secondary outcomes

- proportion of children consuming antibiotics in the first 7 days;
- oral analgesic use in the first 7 days;
- overall symptom burden (crying/distress, disturbed sleep, interference with normal activity, appetite, fever and hearing problems) using a 0-6 Likert scale in the first 7 days;
- number of days with ear pain during follow up (4 weeks);
- number of GP reconsultations with/without subsequent antibiotic prescribing during follow-up;
- adverse events during follow-up;
- complications of AOM during follow-up;
- costs and cost-effectiveness during follow-up;
- generic quality of life of the child at 4 weeks;

For peer review only - http://bmjopen.bmj.com/site/about/guidelines.xhtml

- disease-specific quality of life of the child at 4 weeks;
- parents' and GPs' views of treatment acceptability, usability and satisfaction.

Sample size calculation

The main aim is to demonstrate that analgesic ear drops as added to usual care provide superior parent-reported ear pain relief over the first three days compared to usual care. In our recent trial, the mean parent-reported ear pain score (0-10 scale) over the first three days was 4.36 (SD 2.12) among those allocated to usual care.[14] Patient and Public Involvement (PPI) input from the UK suggested that a 1-point reduction (on a 0-10 scale) would be a clinically important difference.[16] However, our parent panel felt that even a somewhat smaller difference would be clinically meaningful given the low cost, low risk nature of the intervention. To detect a pain reduction of 20% (reflecting a 0.87 point reduction in pain) compared with usual care with 90% power (beta 0.1) and at a 5% significance level (alpha 0.05), 126 children per group will be needed. To allow for 20% attrition, we aim to include 300 participants.

The estimated sample size will also provide substantial power to address the key secondary outcome of the proportion of children consuming antibiotics. Anticipating 47% of children consuming antibiotics in the usual care group as with our previous trial[14], 252 children will provide 89% power (beta 0.11; alpha 0.05) to detect a 20% difference in the proportion of children consuming antibiotics.

Data management

During study conduct, we will comply with the EU General Data Protection Regulation and the Dutch Act on Implementation of the General Data Protection Regulation. The data management department of the Julius Center, UMC Utrecht will be responsible for handling and storage of the trial data using a secured and coded online database (Research Online 2.0 [RO 2.0]). Data will be either entered directly into RO 2.0 or collected on paper forms first. For data entered directly into RO 2.0, processes to promote data quality are added. Coded trial data will be securely stored for 25 years in the main study folder on a central drive of the Julius Center.

Statistical analysis

All analyses will be performed according to the intention-to-treat (ITT) principle and blinded with respect to study-group assignment. The results will be reported in accordance with the CONSORT guidelines.[30]

For peer review only - http://bmjopen.bmj.com/site/about/guidelines.xhtml

Clinical effectiveness

Descriptive statistics will be used to assess any baseline differences between treatment groups. The primary outcome will be analysed with a linear regression model with a residual covariance (i.e. GEE type) matrix to correct for repeated measurements. In main (adjusted) analysis, treatment group, time, a priori specified prognostic factors (baseline ear pain score, duration of ear pain prior to enrolment) and stratification factors (age (<2 years vs 2 years and above), AOM laterality (unilateral vs bilateral), and baseline antibiotic prescribing (yes vs no)) will be included in the model. The validity of the model, i.e. normality and homoscedasticity, will be evaluated by assessing residuals and the comparison between treatment groups will be expressed as differences in means with 95% confidence intervals (CIs). If more than 5% of baseline and outcome data is missing, a sensitivity analysis will be performed using multiple imputation techniques.[31 32]

Secondary outcomes will be analysed using negative-binomial regression analyses for count variables, log binomial regression analyses for dichotomous variables, and linear regression analyses for continuous variables, where applicable adjusted for repeated measurements. For these analyses, the comparison between treatment groups will be expressed as rate ratios, risk ratios, and mean differences, respectively; all with 95% CIs.

We will also conduct pre-specified, exploratory subgroup analyses to investigate whether age (<2 years vs 2 years and above) or AOM laterality (unilateral vs bilateral) modify the effectiveness of analgesic ear drops. In further sensitivity analysis, we will also explore the added impact of antibiotic consumption on the primary outcome of interest.

Cost effectiveness analysis

Alongside the trial, a cost effectiveness analysis from a societal perspective, i.e. including both medical and non-medical costs, will be performed. The societal perspective is particularly relevant in this case, since non-medical costs are particularly relevant in AOM.[33] Those costs mainly relate to parental productivity losses and costs of additional childcare.

Costs analysis

All costs will be estimated at the patient level. Costs of medication use will be retrieved from the Dutch

For peer review only - http://bmjopen.bmj.com/site/about/guidelines.xhtml

formulary and a pharmacist's fee will be added for every prescription.[24 34] Costs of OTC and complementary medicines will be calculated per day, based on current average retail prices. Costs of consulting a GP or a medical specialist, and costs of hospitalisations will be based on current Dutch guidelines for pharmaco-economic evaluation in health care research. Costs of diagnostic tests will also be derived from these guidelines.[34 35] Costs of surgical procedures will be retrieved from a previous Dutch costing study that calculated costs for the different components of surgical procedures.[36]

Resource use such as doctor's visits, prescribed medication (including antibiotics), specialist referrals, hospital admissions, surgical interventions as well as out-of-pocket expenses such as OTC medication, child care, and travel costs will be collected from study diaries filled out by parents. Cost prices will be estimated according to guidelines for pharmaco-economic evaluation in health care research.[34 35] Costs associated with productivity losses will be retrieved from the iPCQ questionnaire and estimated using the friction cost method.[29 37] The hourly cost estimate for childcare will be derived from the Dutch National Institute for Family Finance Information (NIBUD).[38] Travel expenses will be calculated for healthcare visits following the Dutch guideline for economic evaluation.[35] Mean costs per patient will be compared across the randomisation groups.. As the time horizon is only 4 weeks, discounting does not apply.

<u>Cost-effectiveness results</u>

Costs from a societal perspective will be compared with the parent-reported ear pain scores over the first three days. We will calculate incremental cost-effectiveness ratios by dividing the estimated differences in costs between groups by the differences in effects observed, i.e. the additional cost per additional 1 point reduction in mean ear pain score over the first three days for the analgesic ear drops group versus the usual care group. Uncertainty will be addressed by means of two-stage non-parametric bootstrap sampling. The effect of any difference in baseline characteristics on cost-effectiveness results will be investigated using net benefit regression methods. In case of missing data, we will use multiple imputation techniques. The final results will be presented using incremental cost-effectiveness planes and cost-effectiveness acceptability curves.

Monitoring and safety

The trial will be monitored regularly by an independent research data monitor of the UMC Utrecht to ensure the quality of the trial execution. Since we do not expect any safety issues and the overall risk of this trial was

For peer review only - http://bmjopen.bmj.com/site/about/guidelines.xhtml

judged negligible, we have not established a data safety monitoring board and refrain from conducting a safety interim analysis. However, in accordance to section 10, subsection 4, of the WMO, the sponsor will suspend the study if there is sufficient ground that continuation of the study will jeopardise subject health or safety.

ETHICS AND DISSIMINATION

The study will be conducted according to the principles of the Declaration of Helsinki (10th version, October 2013), the ICH GCP E6(R2) guideline, the Dutch Law on medicinal products ('geneesmiddelenwet') and in accordance with the Medical Research Involving Human Subjects Act (WMO). The Medical Research Ethics Committee Utrecht, the Netherlands, has approved the protocol (21-447/G-D).

Research findings will be disseminated through publications in peer reviewed medical journals targeted towards the wider audience of health professionals involved in the care of children and presented at relevant national and international scientific meetings. We will work with our parent panel to help interpret the findings of the trial and harness their resources for dissemination to the lay public.

PATIENT AND PUBLIC INVOLVEMENT

A panel of eight parents was established for this trial. This panel helped to shape the grant proposal and final study protocol during online focus group meetings, with particular advice given on patient-relevant outcomes including the minimally important clinical differences, recruitment strategy, and the design of data collection. The parent panel will be actively involved throughout all critical stages of the trial through regular parent panel meetings.

CURRENT STUDY STATUS

Study enrolment is ongoing with the first participant being included on 6 October 2021.

For peer review only - http://bmjopen.bmj.com/site/about/guidelines.xhtml

DISCUSSION

Up to now, evidence on the use of analgesic ear drops in children with AOM is limited. A 2011 systematic review included five studies of which two (117 children) compared analgesic ear drops to placebo.[15] The authors concluded that these trials provide limited evidence that a single dose of analgesic ear drops is effective in reducing ear pain 30 minutes after administration in older children (age 3 to 19 years) with AOM.[15] Since then, a UK-based trial compared at-home, repeated dosing, of analgesic ear drops with placebo ear drops and usual care. This trial was terminated prematurely due to operational issues.[16] Initial data of 84 children recruited in primary care indicate that ear pain levels were lower in both ear drops groups than in the usual care group. However, these estimated treatment effects are imprecise because of the low sample size target and therefore require confirmation in future studies.

Our trial aims to do so by assessing the clinical and cost effectiveness of analgesic eardrops in children with AOM, as well as views and experiences of parents and GPs through the nested process evaluation. The pragmatic, open design enhances the applicability of the trial findings to daily practice and is most suited to address the key secondary outcome of the impact of the analgesic ear drops on the proportion of children consuming antibiotics in everyday practice.[39] This would be much more difficult to determine realistically in a placebo-blinded study where children in both groups would receive ear drops (either placebo or analgesic drops); such 'treatment' may in itself lead to altered parental (health care seeking) behaviour. Additionally, the open design, in which the control group receives usual care, is most suited for 'real world' cost-effectiveness analysis. However, the lack of blinding in combination with the subjective, parent-reported outcomes might also lead to bias in the assessment of analgesic effects. To minimise this potential bias, the study physician will emphasize during the baseline visit that it is not known whether the drops have any effect in children with AOM. Furthermore, a recent meta-epidemiological study found no evidence for a difference in estimated treatment effect between blinded and non-blinded trials.[40] Our design does not control for the placebo effects, but will more closely reflect the benefits that would accrue in everyday practice with regard to analgesic effects.

Albeit not recommended in the Dutch AOM clinical practice guideline[23], the OTC availability of lidocaine eardrops may inherently incur the risk of contamination, i.e. parents in the control group buying lidocaine ear drops themselves. We consider this unlikely since only 5% (10/221) of participants did use lidocaine ear drops in our previous trial of a GP-targeted educational intervention to improve pain management children with

For peer review only - http://bmjopen.bmj.com/site/about/guidelines.xhtml

AOM.[14] We will, however, capture data on the use of lidocaine ear drops in all trial participants to monitor potential contamination.

ACKNOWLEDGEMENTS

We gratefully thank our parent panel for their valuable input and all children and parents who participated in our trial thus far.

AUTHOR'S CONTRIBUTIONS

RPV, AGMS and RAMJD designed the trial. JLHdS, SH and RPV drafted the first version of the protocol paper. All other authors (RAMJD, AGMS, EAMS, GAdW, NPAZ, LY, SA, PL and ADH) provided feedback on this version. All authors (JLHdS, SH, RPV, RAMJD, AGMS, EAMS, GAdW, NPAZ, LY, SA, PL and ADH) approved the final version of the manuscript.

FUNDING STATEMENT

The trial is supported by a grant from the Netherlands Organisation for Health Research and Development (ZonMw– grant number 10060011910003). The funding agency had no role in the design, and will not have any role during execution of the trial, data-analyses, interpretation of the data or decision to submit results. LY is an NIHR Senior Investigator and her research programme is partly supported by NIHR Applied Research Collaboration (ARC)-West, NIHR Health Protection Research Unit (HPRU) for Behavioural Science and Evaluation, and the NIHR Southampton Biomedical Research Centre (BRC).

COMPETING INTERESTS

The authors declare that they have no conflict of interest.

For peer review only

REFERENCES

1. Hullegie S, Schilder AGM, Marchisio P, et al. A Strong Decline in the Incidence of Childhood Otitis Media During the COVID-19 Pandemic in the Netherlands. *Front Cell Infect Microbiol* 2021;11:768377. doi: 10.3389/fcimb.2021.768377 [published Online First: 2021/11/19]
2. Kaur R, Schulz S, Fuji N, et al. COVID-19 Pandemic Impact on Respiratory Infectious Diseases in Primary Care Practice in Children. *Front Pediatr* 2021;9:722483. doi: 10.3389/fped.2021.722483 [published Online First: 2021/10/01]
3. McBride JA, Eickhoff J, Wald ER. Impact of COVID-19 Quarantine and School Cancelation on Other Common Infectious Diseases. *Pediatr Infect Dis J* 2020;39(12):e449-e52. doi: 10.1097/inf.0000000000002883 [published Online First: 2020/10/09]
4. Gulliford M, Latinovic R, Charlton J, et al. Selective decrease in consultations and antibiotic prescribing for acute respiratory tract infections in UK primary care up to 2006. *J Public Health (Oxf)* 2009;31(4):512-20. doi: 10.1093/pubmed/fdp081 [published Online First: 2009/09/08]
5. van den Broek d'Obrenan J, Verheij TJ, Numans ME, et al. Antibiotic use in Dutch primary care: relation between diagnosis, consultation and treatment. *J Antimicrob Chemother* 2014;69(6):1701-7. doi: 10.1093/jac/dku005 [published Online First: 2014/02/11]
6. Barber C, Ille S, Vergison A, et al. Acute otitis media in young children - what do parents say? *Int J Pediatr Otorhinolaryngol* 2014;78(2):300-6. doi: 10.1016/j.ijporl.2013.11.030 [published Online First: 2014/01/01]
7. Hansen MP, Howlett J, Del Mar C, et al. Parents' beliefs and knowledge about the management of acute otitis media: a qualitative study. *BMC Fam Pract* 2015;16:82. doi: 10.1186/s12875-015-0297-7 [published Online First: 2015/07/08]
8. Venekamp RP, Sanders SL, Glasziou PP, et al. Antibiotics for acute otitis media in children. *Cochrane Database Syst Rev* 2015(6):CD000219. doi: 10.1002/14651858.CD000219.pub4 [published Online First: 2015/06/24]
9. Thompson M, Vodicka TA, Blair PS, et al. Duration of symptoms of respiratory tract infections in children: systematic review. *BMJ* 2013;347:f7027. doi: 10.1136/bmj.f7027 [published Online First: 2013/12/18]
10. Ovnat Tamir S, Shemesh S, Oron Y, et al. Acute otitis media guidelines in selected developed and developing countries: uniformity and diversity. *Arch Dis Child* 2017;102(5):450-57. doi: 10.1136/archdischild-2016-310729 [published Online First: 2016/09/08]
11. Costelloe C, Metcalfe C, Lovering A, et al. Effect of antibiotic prescribing in primary care on antimicrobial resistance in individual patients: systematic review and meta-analysis. *Bmj* 2010;340:c2096. doi: 10.1136/bmj.c2096 [published Online First: 2010/05/21]
12. Gillies M, Ranakusuma A, Hoffmann T, et al. Common harms from amoxicillin: a systematic review and meta-analysis of randomized placebo-controlled trials for any indication. *Cmaj* 2015;187(1):E21-e31. doi: 10.1503/cmaj.140848 [published Online First: 2014/11/19]
13. Pouwels KB, Dolk FCK, Smith DRM, et al. Actual versus 'ideal' antibiotic prescribing for common conditions in English primary care. *J Antimicrob Chemother* 2018;73(suppl_2):19-26. doi: 10.1093/jac/dkx502 [published Online First: 2018/03/01]
14. van Uum RT, Venekamp RP, Zuithoff NP, et al. Improving pain management in childhood acute otitis media in general practice: a cluster randomised controlled trial of a GP-targeted educational intervention. *Br J Gen Pract* 2020;70(699):e684-e95. doi: 10.3399/bjgp20X712589 [published Online First: 2020/08/26]
15. Foxlee R, Johansson A, Wejfalk J, et al. Topical analgesia for acute otitis media. *Cochrane Database Syst Rev* 2006(3):Cd005657. doi: 10.1002/14651858.CD005657.pub2 [published Online First: 2006/07/21]
16. Hay AD, Downing H, Francis NA, et al. Anaesthetic-analgesic ear drops to reduce antibiotic consumption in children with acute otitis media: the CEDAR RCT. *Health Technol Assess* 2019;23(34):1-48. doi: 10.3310/hta23340 [published Online First: 2019/07/16]
17. Horne R. Treatment perceptions and self-regulation. In: Leventhal H, Cameron L, eds. The self-regulation of health and illness behaviour. London: Routledge 2003:138–53.
18. Yardley L, Douglas E, Anthierens S, et al. Evaluation of a web-based intervention to reduce antibiotic prescribing for LRTI in six European countries: quantitative process analysis of the GRACE/INTRO randomised controlled trial. *Implement Sci* 2013;8:134. doi: 10.1186/1748-5908-8-134 [published Online First: 2013/11/19]
19. van Uum RT, Sjoukes A, Venekamp RP, et al. Pain management in acute otitis media: a qualitative study exploring GPs' views and expectations parallel to a trial of an educational intervention. *BJGP Open*

For peer review only - http://bmjopen.bmj.com/site/about/guidelines.xhtml

2018;2(4):bjgpopen18X101620. doi: 10.3399/bjgpopen18X101620 [published Online First: 2019/02/07]
20. van Uum RT, Venekamp RP, Schilder AGM, et al. Pain management in acute otitis media: a qualitative study of parents' views and expectations. *BMC Fam Pract* 2019;20(1):18. doi: 10.1186/s12875-019-0908-9 [published Online First: 2019/01/25]
21. Braun V, Clarke V. What can "thematic analysis" offer health and wellbeing researchers? *Int J Qual Stud Health Well-being* 2014;9:26152. doi: 10.3402/qhw.v9.26152 [published Online First: 2014/10/19]
22. Gale NK, Heath G, Cameron E, et al. Using the framework method for the analysis of qualitative data in multi-disciplinary health research. *BMC Med Res Methodol* 2013;13:117. doi: 10.1186/1471-2288-13-117 [published Online First: 2013/09/21]
23. Damoiseaux RAMJ, Venekamp RP, Eekhof J. NHG-standaard OMA bij kinderen (derde herziening). *Huisarts Wet* 2014;57:648.
24. Farmacotherapeutisch Kompas [Available from: https://www.farmacotherapeutischkompas.nl/.
25. Dutch Children's Formulary 'Kinderformularium' [Available from: https://www.kinderformularium.nl/.
26. Little P, Gould C, Williamson I, et al. Pragmatic randomised controlled trial of two prescribing strategies for childhood acute otitis media. *Bmj* 2001;322(7282):336-42. doi: 10.1136/bmj.322.7282.336 [published Online First: 2001/02/13]
27. Raat H, Landgraf JM, Oostenbrink R, et al. Reliability and validity of the Infant and Toddler Quality of Life Questionnaire (ITQOL) in a general population and respiratory disease sample. *Qual Life Res* 2007;16(3):445-60. doi: 10.1007/s11136-006-9134-8 [published Online First: 2006/11/18]
28. Rosenfeld RM, Goldsmith AJ, Tetlus L, et al. Quality of life for children with otitis media. *Arch Otolaryngol Head Neck Surg* 1997;123(10):1049-54. doi: 10.1001/archotol.1997.01900100019002 [published Online First: 1997/10/27]
29. Bouwmans C, Krol M, Brouwer W, et al. IMTA Productivity Cost Questionnaire (IPCQ). *Value Health* 2014;17(7):A550. doi: 10.1016/j.jval.2014.08.1791 [published Online First: 2014/11/01]
30. Schulz KF, Altman DG, Moher D. CONSORT 2010 statement: updated guidelines for reporting parallel group randomised trials. *Bmj* 2010;340:c332. doi: 10.1136/bmj.c332 [published Online First: 2010/03/25]
31. Donders AR, van der Heijden GJ, Stijnen T, et al. Review: a gentle introduction to imputation of missing values. *J Clin Epidemiol* 2006;59(10):1087-91. doi: 10.1016/j.jclinepi.2006.01.014 [published Online First: 2006/09/19]
32. Rubin D. Multiple imputation for non-response in surveys. New York: John Wiley 1987.
33. Wolleswinkel-van den Bosch JH, Stolk EA, Francois M, et al. The health care burden and societal impact of acute otitis media in seven European countries: results of an Internet survey. *Vaccine* 2010;28 Suppl 6:G39-52. doi: 10.1016/j.vaccine.2010.06.014 [published Online First: 2010/11/16]
34. Zorginstituut Nederland. Kostenhandleiding: Methodologie van kostenonderzoek en referentieprijzen voor economische evaluaties in de gezondheidszorg. 2016 ed. Diemen: Zorginstituut Nederland 2016.
35. Zorginstituut Nederland. Richtlijn voor het uitvoeren van economische evaluaties in de gezondheidszorg. Diemen: Zorginstituut Nederland 2016.
36. Buskens E, van Staaij B, van den Akker J, et al. Adenotonsillectomy or watchful waiting in patients with mild to moderate symptoms of throat infections or adenotonsillar hypertrophy: a randomized comparison of costs and effects. *Arch Otolaryngol Head Neck Surg* 2007;133(11):1083-8. doi: 10.1001/archotol.133.11.1083 [published Online First: 2007/11/21]
37. Koopmanschap MA, Rutten FF, van Ineveld BM, et al. The friction cost method for measuring indirect costs of disease. *J Health Econ* 1995;14(2):171-89. doi: 10.1016/0167-6296(94)00044-5 [published Online First: 1995/05/08]
38. National Institute for Family Finance Information website [Available from: https://www.nibud.nl/consumenten/kosten-van-kinderopvang/.
39. Treweek S, Zwarenstein M. Making trials matter: pragmatic and explanatory trials and the problem of applicability. *Trials* 2009;10:37. doi: 10.1186/1745-6215-10-37 [published Online First: 2009/06/06]
40. Moustgaard H, Clayton GL, Jones HE, et al. Impact of blinding on estimated treatment effects in randomised clinical trials: meta-epidemiological study. *Bmj* 2020;368:l6802. doi: 10.1136/bmj.l6802 [published Online First: 2020/01/23]

For peer review only - http://bmjopen.bmj.com/site/about/guidelines.xhtml

Figure 1. Flow diagram of study procedures

Legend: *AOM: acute otitis media, GP: general practitioner, OPTIMA: trial acronym*

Supplemental material 1. Methods and analyses mixed-method process evaluation

Supplemental material 2. Informed consent form

For peer review only

For peer review only - http://bmjopen.bmj.com/site/about/guidelines.xhtml

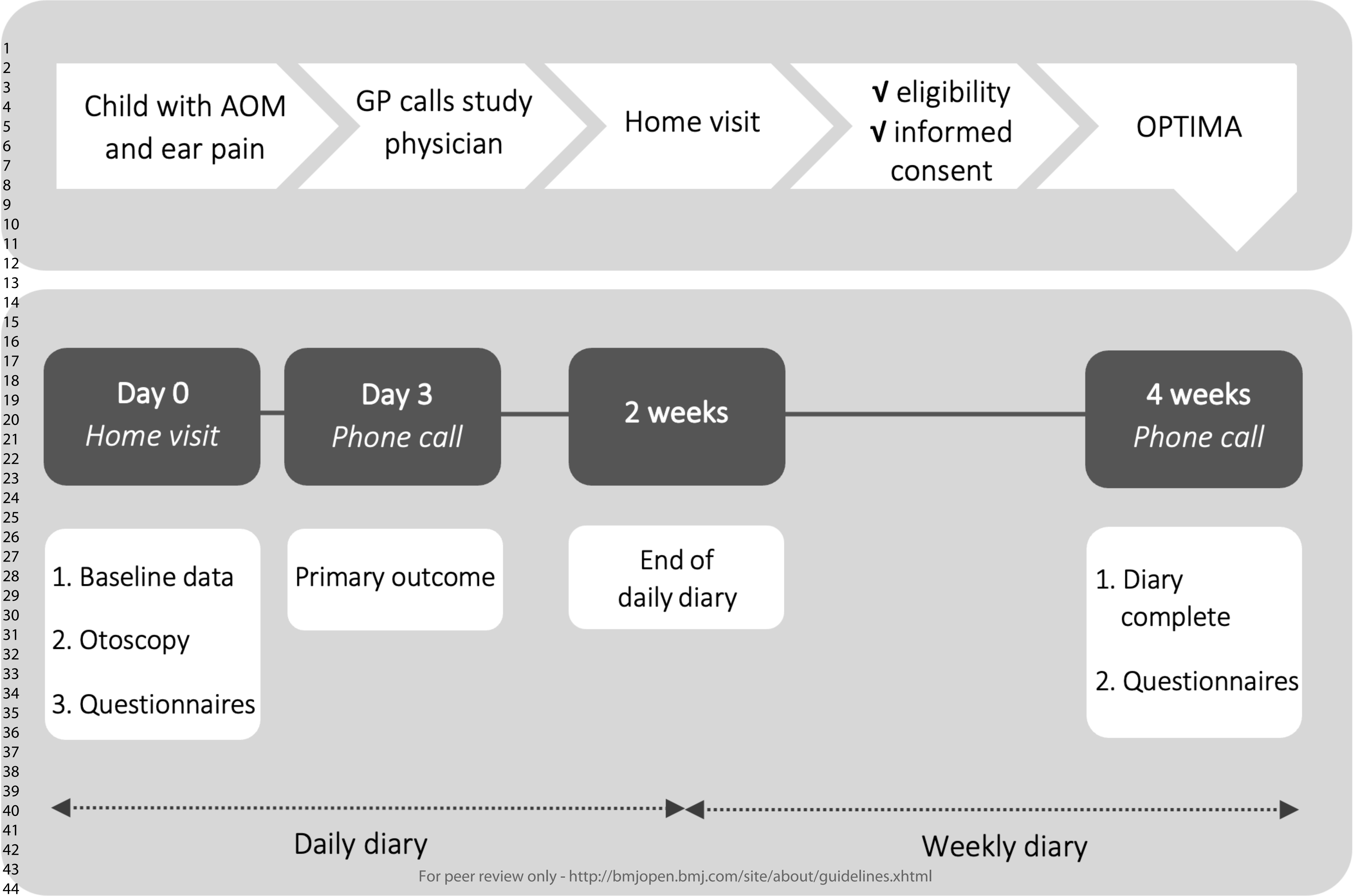
Child with AOM and ear pain
GP calls study physician
Home visit
√ eligibility
√ informed consent
OPTIMA
Day 0
Home visit
Day 3
Phone call
2 weeks
4 weeks
Phone call
1. Baseline data
2. Otoscopy
3. Questionnaires
Primary outcome
End of daily diary
1. Diary complete
2. Questionnaires
Daily diary
Weekly diary

SPIRIT — Standard Protocol Items: Recommendations for Interventional Trials

SPIRIT 2013 Checklist: Recommended items to address in a clinical trial protocol and related documents*

Section/item	Item No	Description	Page number
**Administrative information**			
Title	1	Descriptive title identifying the study design, population, interventions, and, if applicable, trial acronym	1
Trial registration	2a	Trial identifier and registry name. If not yet registered, name of intended registry	2
	2b	All items from the World Health Organization Trial Registration Data Set	2
Protocol version	3	Date and version identifier	n/a
Funding	4	Sources and types of financial, material, and other support	13
Roles and responsibilities	5a	Names, affiliations, and roles of protocol contributors	1
	5b	Name and contact information for the trial sponsor	n/a
	5c	Role of study sponsor and funders, if any, in study design; collection, management, analysis, and interpretation of data; writing of the report; and the decision to submit the report for publication, including whether they will have ultimate authority over any of these activities	13
	5d	Composition, roles, and responsibilities of the coordinating centre, steering committee, endpoint adjudication committee, data management team, and other individuals or groups overseeing the trial, if applicable (see Item 21a for data monitoring committee)	11
**Introduction**			
Background and rationale	6a	Description of research question and justification for undertaking the trial, including summary of relevant studies (published and unpublished) examining benefits and harms for each intervention	4
	6b	Explanation for choice of comparators	4
Objectives	7	Specific objectives or hypotheses	4

For peer review only - http://bmjopen.bmj.com/site/about/guidelines.xhtml

Trial design	8	Description of trial design including type of trial (eg, parallel group, crossover, factorial, single group), allocation ratio, and framework (eg, superiority, equivalence, noninferiority, exploratory)	5

Methods: Participants, interventions, and outcomes

Study setting	9	Description of study settings (eg, community clinic, academic hospital) and list of countries where data will be collected. Reference to where list of study sites can be obtained	5
Eligibility criteria	10	Inclusion and exclusion criteria for participants. If applicable, eligibility criteria for study centres and individuals who will perform the interventions (eg, surgeons, psychotherapists)	5,16 (Box 1)
Interventions	11a	Interventions for each group with sufficient detail to allow replication, including how and when they will be administered	6
	11b	Criteria for discontinuing or modifying allocated interventions for a given trial participant (eg, drug dose change in response to harms, participant request, or improving/worsening disease)	6
	11c	Strategies to improve adherence to intervention protocols, and any procedures for monitoring adherence (eg, drug tablet return, laboratory tests)	6
	11d	Relevant concomitant care and interventions that are permitted or prohibited during the trial	n/a
Outcomes	12	Primary, secondary, and other outcomes, including the specific measurement variable (eg, systolic blood pressure), analysis metric (eg, change from baseline, final value, time to event), method of aggregation (eg, median, proportion), and time point for each outcome. Explanation of the clinical relevance of chosen efficacy and harm outcomes is strongly recommended	7,8
Participant timeline	13	Time schedule of enrolment, interventions (including any run-ins and washouts), assessments, and visits for participants. A schematic diagram is highly recommended (see Figure)	5,6, Figure 1
Sample size	14	Estimated number of participants needed to achieve study objectives and how it was determined, including clinical and statistical assumptions supporting any sample size calculations	8
Recruitment	15	Strategies for achieving adequate participant enrolment to reach target sample size	5

Methods: Assignment of interventions (for controlled trials)

Allocation:

Sequence generation	16a	Method of generating the allocation sequence (eg, computer-generated random numbers), and list of any factors for stratification. To reduce predictability of a random sequence, details of any planned restriction (eg, blocking) should be provided in a separate document that is unavailable to those who enrol participants or assign interventions	6
Allocation concealment mechanism	16b	Mechanism of implementing the allocation sequence (eg, central telephone; sequentially numbered, opaque, sealed envelopes), describing any steps to conceal the sequence until interventions are assigned	6
Implementation	16c	Who will generate the allocation sequence, who will enrol participants, and who will assign participants to interventions	6
Blinding (masking)	17a	Who will be blinded after assignment to interventions (eg, trial participants, care providers, outcome assessors, data analysts), and how	n/a (open trial)
	17b	If blinded, circumstances under which unblinding is permissible, and procedure for revealing a participant's allocated intervention during the trial	n/a
**Methods: Data collection, management, and analysis**			
Data collection methods	18a	Plans for assessment and collection of outcome, baseline, and other trial data, including any related processes to promote data quality (eg, duplicate measurements, training of assessors) and a description of study instruments (eg, questionnaires, laboratory tests) along with their reliability and validity, if known. Reference to where data collection forms can be found, if not in the protocol	5-7
	18b	Plans to promote participant retention and complete follow-up, including list of any outcome data to be collected for participants who discontinue or deviate from intervention protocols	5,6
Data management	19	Plans for data entry, coding, security, and storage, including any related processes to promote data quality (eg, double data entry; range checks for data values). Reference to where details of data management procedures can be found, if not in the protocol	8
Statistical methods	20a	Statistical methods for analysing primary and secondary outcomes. Reference to where other details of the statistical analysis plan can be found, if not in the protocol	9,10
	20b	Methods for any additional analyses (eg, subgroup and adjusted analyses)	9,10

	20c	Definition of analysis population relating to protocol non-adherence (eg, as randomised analysis), and any statistical methods to handle missing data (eg, multiple imputation)	9,10
**Methods: Monitoring**			
Data monitoring	21a	Composition of data monitoring committee (DMC); summary of its role and reporting structure; statement of whether it is independent from the sponsor and competing interests; and reference to where further details about its charter can be found, if not in the protocol. Alternatively, an explanation of why a DMC is not needed	11
	21b	Description of any interim analyses and stopping guidelines, including who will have access to these interim results and make the final decision to terminate the trial	11
Harms	22	Plans for collecting, assessing, reporting, and managing solicited and spontaneously reported adverse events and other unintended effects of trial interventions or trial conduct	6
Auditing	23	Frequency and procedures for auditing trial conduct, if any, and whether the process will be independent from investigators and the sponsor	n/a
**Ethics and dissemination**			
Research ethics approval	24	Plans for seeking research ethics committee/institutional review board (REC/IRB) approval	11
Protocol amendments	25	Plans for communicating important protocol modifications (eg, changes to eligibility criteria, outcomes, analyses) to relevant parties (eg, investigators, REC/IRBs, trial participants, trial registries, journals, regulators)	n/a
Consent or assent	26a	Who will obtain informed consent or assent from potential trial participants or authorised surrogates, and how (see Item 32)	5, supplemental material 2
	26b	Additional consent provisions for collection and use of participant data and biological specimens in ancillary studies, if applicable	supplemental material 2
Confidentiality	27	How personal information about potential and enrolled participants will be collected, shared, and maintained in order to protect confidentiality before, during, and after the trial	8
Declaration of interests	28	Financial and other competing interests for principal investigators for the overall trial and each study site	13
Access to data	29	Statement of who will have access to the final trial dataset, and disclosure of contractual agreements that limit such access for investigators	8

review only

For peer review only - http://bmjopen.bmj.com/site/about/guidelines.xhtml

Ancillary and post-trial care	30	Provisions, if any, for ancillary and post-trial care, and for compensation to those who suffer harm from trial participation	n/a
Dissemination policy	31a	Plans for investigators and sponsor to communicate trial results to participants, healthcare professionals, the public, and other relevant groups (eg, via publication, reporting in results databases, or other data sharing arrangements), including any publication restrictions	11
	31b	Authorship eligibility guidelines and any intended use of professional writers	n/a
	31c	Plans, if any, for granting public access to the full protocol, participant-level dataset, and statistical code	n/a
**Appendices**			
Informed consent materials	32	Model consent form and other related documentation given to participants and authorised surrogates	Supplemental material 2
Biological specimens	33	Plans for collection, laboratory evaluation, and storage of biological specimens for genetic or molecular analysis in the current trial and for future use in ancillary studies, if applicable	n/a

*It is strongly recommended that this checklist be read in conjunction with the SPIRIT 2013 Explanation & Elaboration for important clarification on the items. Amendments to the protocol should be tracked and dated. The SPIRIT checklist is copyrighted by the SPIRIT Group under the Creative Commons "Attribution-NonCommercial-NoDerivs 3.0 Unported" license.

For peer review only

Supplemental material 1. Methods and analyses mixed-method process evaluation

Surveys

Surveys will include brief statements which will be rated by parents on a 5-point scale (strongly disagree/ strongly agree). Parents of all trial participants will complete this survey at baseline and at 4 weeks (end of follow-up). Beliefs about the necessity and harms of antibiotics for AOM, oral analgesics and analgesic eardrops were assessed using questions based on the two key dimensions of treatment perceptions in the extended Common Sense Model: 'It is usually necessary to take antibiotics/oral analgesics/analgesic eardrops for an ear infection' and 'Taking antibiotics/oral analgesics/analgesic eardrops for an ear infection can do more harm than good'.[17] Patient satisfaction with the consultation was assessed by a three-item Consultation Satisfaction Questionnaire that assessed whether they felt they had received all the information and advice they needed and were generally satisfied with the consultation.[18]

Semi-structured interviews

Semi-structured interviews will be carried out with parents and GPs participating in the trial. Interviews with parents will capture experiences of and satisfaction with care and subsequent views and experiences of management of symptoms (including eardrops and antibiotic use). Parents will be recruited from both the intervention and control arm and will be purposively sampled after study participation to obtain variation in age of child, symptom presentation, antibiotic prescribed, eardrops prescribed, gender, number of children, education level and employment status. Interviews will be carried out preferably within 2 weeks after study participation. Interviews with GPs will capture views and experiences of AOM consultations and the use of eardrops in these consultations. GPs will be purposively sampled to obtain variation in practice setting, gender, age and experience. Interviews with GPs will be carried out towards the end of the inclusion period. Data will be collected until saturation is reached; based on previous experience, we expect to conduct approximately 10 to 20 semi-structured interviews with both parents and GPs to reach saturation.[19 20]

Each interview will be audio recorded with the participant's permission. Recordings will allow verbatim transcription of interviews in Microsoft Word. Once transcripts are checked, recordings will be deleted. Transcripts will be labelled with a unique participant number and will omit any identifiable data either identifying the participant or their general practice.

Analysis of qualitative data

Qualitative data collection by means of semi-structured interviews and qualitative data analysis will be done concurrently. During the process, multiple members of the study team will review the transcripts of earlier interviews for accuracy and decide whether the topic lists need adaptation.

Interviews with parents and GPs, will be analysed using thematic and framework analysis taking an inductive approach.[21 22] NVivo software will be used to assist with the organisation of data. A thematic framework will be used to chart data across all interviews and will aid comparisons between participants.

For peer review only

Proefpersoneninformatie en toestemmingsformulier voor deelname aan medisch-wetenschappelijk onderzoek

OPTIMA: pijnstillende oordruppels bij acute middenoorontsteking

Officiële titel: Effectiviteit van pijnstillende oordruppels als toevoeging aan systemische pijnstillers bij kinderen met otitis media acuta: een pragmatische gerandomiseerde gecontroleerde studie

Inleiding

Geachte heer/mevrouw,

Met deze informatiebrief willen we u vragen of u met uw kind wilt meedoen aan medisch-wetenschappelijk onderzoek. Meedoen is vrijwillig. U krijgt deze brief omdat uw kind een acute middenoorontsteking heeft.
U leest hier om wat voor onderzoek het gaat, wat het voor u betekent, en wat de voordelen en nadelen zijn. Het is veel informatie. Wilt u de informatie doorlezen en beslissen of u wilt meedoen? Als u wilt meedoen, kunt u het formulier invullen dat u vindt in bijlage C.

Stel uw vragen

U kunt uw beslissing nemen met de informatie die u in deze informatiebrief vindt. Daarnaast raden we u aan om dit te doen:
- Stel vragen aan de onderzoeker die u deze informatie geeft.
- Praat met uw partner, familie of vrienden over dit onderzoek.
- Stel vragen aan de onafhankelijk deskundige, Dr. P.C.J.L. Bruijning-Verhagen (zie bijlage A voor contactgegevens).
- Lees de informatie op www.rijksoverheid.nl/mensenonderzoek.

1. Algemene informatie

Het Universitair Medisch Centrum (UMC) Utrecht heeft dit onderzoek opgezet. Voor dit onderzoek zijn 300 patiënten nodig. Dit onderzoek wordt gefinancierd door ZonMw. Deze organisatie financiert gezondheidsonderzoek in opdracht van het Ministerie van Volksgezondheid, Welzijn en Sport (VWS), en de Nederlandse Organisatie voor Wetenschappelijk Onderzoek (NWO).
De medisch-ethische toetsingscommissie Utrecht heeft dit onderzoek goedgekeurd.

For peer review only - http://bmjopen.bmj.com/site/about/guidelines.xhtml

Optima
pijnstillende oordruppels bij otitis media acuta

UMC Utrecht

2. Wat is het doel van het onderzoek?

In dit onderzoek bekijken we wat het effect is van pijnstillende oordruppels bij kinderen met een acute middenoorontsteking, een ontsteking van de holte achter het trommelvlies.

3. Wat is de achtergrond van het onderzoek?

Een acute middenoorontsteking komt vaak voor op de kinderleeftijd. Het wordt veroorzaakt door een virus of een bacterie. Standaardzorg bestaat momenteel voornamelijk uit pijnstillende behandeling (paracetamol en zo nodig ibuprofen) en indien nodig antibiotica. Ondanks de inzet van deze standaardzorg, gaat de aandoening gepaard met oorpijn en ongemak bij kinderen en kan het een aanzienlijke impact op het gehele gezin hebben door slapeloze nachten en school- en werkverzuim. Het is daarom belangrijk dat er gezocht blijft worden naar andere behandelingen die de klachten kunnen verlichten. Eerdere studies lieten zien dat pijnstillende oordruppels mogelijk een gunstig effect hebben, maar het huidige bewijs is van onvoldoende kwaliteit om een zekere uitspraak over het effect te kunnen doen. Daarom willen we in deze studie onderzoeken of de pijnstillende oordruppels een betere verlichting van de oorpijn geven dan de gebruikelijke pijnstillers alleen. We gaan dit onderzoeken door 300 kinderen middels loting toe te wijzen aan 1) pijnstillende oordruppels bovenop de standaardzorg of 2) standaardzorg

4. Hoe verloopt het onderzoek?

Hoelang duurt het onderzoek?

Doet uw kind mee met het onderzoek? Dan duurt dat in totaal 4 weken.

Stap 1: is uw kind geschikt om mee te doen?

We willen eerst weten of uw kind geschikt is om mee te doen. Uw huisarts controleert of uw kind in aanmerking komt voor studiedeelname. Vervolgens geeft uw huisarts – met uw goedkeuring – uw gegevens door aan het onderzoeksteam in het UMC Utrecht en geeft u deze informatiefolder mee. Het onderzoeksteam neemt telefonisch contact met u op en één van de onderzoekers bezoekt u thuis, binnen 24 uur na uw bezoek aan de huisarts.. Aanvullend aan de beoordeling van de huisarts, zal de onderzoeker u enkele vragen stellen als extra controle op geschiktheid voor deelname.
Tijdens dit bezoek zal de onderzoeker u verder informeren over de studie en mogelijke vragen die u heeft beantwoorden. Pas daarna besluit u of u met uw kind wilt deelnemen aan het onderzoek. Indien u besluit om mee te doen vragen we aan beide ouders/voogden om een toestemmingsformulier te ondertekenen. De onderzoeker ondertekent dit formulier ook. Indien één van de ouders/voogden tijdens het eerste huisbezoek niet aanwezig is, zal van de betreffende ouder mondeling toestemming worden gevraagd. Alleen indien deze ook (mondelinge) toestemming geeft voor deelname, kan uw kind deelnemen aan het onderzoek. In het betreffende geval zal een extra proefpersoneninformatie en toestemmingsformulier bij u thuis worden achtergelaten om door de andere ouder te laten ondertekenen. Het getekende formulier zal op een ander moment opgehaald worden. In bijlage D staat

For peer review only - http://bmjopen.bmj.com/site/about/guidelines.xhtml

bovenstaande procedure van informeren en toestemming geven (informed consent) op een schematische manier weergegeven.

Stap 2: de behandeling

Voor dit onderzoek verdelen we de deelnemende kinderen gelijk over 2 groepen:

- **Groep 1 (interventie groep)**. De kinderen in deze groep krijgen pijnstillende oordruppels (Otalgan® maximaal 6 keer/dag 1-2 druppels gedurende 7 dagen) bovenop de standaardzorg bestaande uit gebruikelijke pijnstillers en indien nodig naar inschatting van de huisarts antibiotica (50% van de kinderen).
  Let op: indien uw kind een loopoor krijgt dan moet u **direct stoppen** met het gebruik van de pijnstillende oordruppels in dat oor, neem in dat geval contact op met de onderzoeker.
- **Groep 2 (controle groep)**. De kinderen in deze groep krijgen de standaardzorg (50% van de kinderen).

Loting bepaalt in welke groep uw kind terecht zal komen.

Stap 3: verzamelen van gegevens

- Het **huisbezoek** op de dag van studiedeelname duurt circa één uur. Tijdens dit huisbezoek zal, nadat er toestemming gegeven is, een kort lichamelijk onderzoek worden verricht waarbij in de oren van uw kind gekeken wordt. Daarnaast neemt de onderzoeker de eerste vragenlijsten met u door. Aan het einde van het eerste huisbezoek zal de arts-onderzoeker via internet contact opnemen met het onderzoekscentrum in het UMC Utrecht om te zien wat het resultaat is van de loting die bepaalt welke behandeling uw kind krijgt. Dit geven wij door aan de huisarts en apotheek van uw kind. Indien uw kind in groep 1 ingedeeld wordt, ontvangt u aan het einde van het bezoek de oordruppels.
- We vragen u gedurende 4 weken om dagelijks een **klachtendagboekje** bij te houden. Dit kan online of op papier gedaan worden. U krijgt hier uitleg over tijdens het huisbezoek.
- Na **3 dagen** neemt een medewerker van het onderzoek **telefonisch contact** met u op om het dagboekje en eventuele vragen te bespreken. Dit duurt circa 10 minuten.
- Na **4 weken** neemt de onderzoeker nogmaals **telefonisch contact** met u op om de studie af te ronden en de laatste vragenlijsten met u door te nemen. Dit telefoon gesprek duurt maximaal 30 minuten.
- Optioneel kunt u ook nog deelnemen aan een telefonisch interview over uw ervaringen met de behandeling van de acute middenoorontsteking en de studie. Dit gebeurt alleen indien u hier toestemming voor geeft.

Wat is er anders dan bij gewone zorg?

Er is bij dit onderzoek niet zoveel anders dan bij gewone zorg. Indien uw kind in groep 1 geloot wordt, krijgt uw kind pijnstillende oordruppels bovenop de gewone zorg. Indien uw kind in groep 2 geplaatst wordt krijgt uw kind de gewone zorg.

For peer review only - http://bmjopen.bmj.com/site/about/guidelines.xhtml

5. Welke afspraken maken we met u?

We willen graag dat het onderzoek goed verloopt. Daarom maken we de volgende afspraken met u:

- U geeft het medicijn aan uw kind op de manier die de onderzoeker u heeft uitgelegd.
- Uw kind doet tijdens dit onderzoek niet ook nog mee aan een ander medisch-wetenschappelijk onderzoek.
- U vult de vragenlijsten en het dagboekje in zoals met u besproken is.
- U draagt de deelnemerskaart van het onderzoek bij u. Bijvoorbeeld in uw portemonnee. Hierop staat dat uw kind meedoet aan dit onderzoek. En wie men moet waarschuwen bij een noodsituatie. Laat deze kaart zien als uw kind bij een (andere) arts komt.
- Uw huisarts blijft ten alle tijden verantwoordelijk voor de medische behandeling van uw kind. Bij eventuele gezondheidsproblemen van uw kind neemt u contact op met uw huisarts zoals u normaal gesproken ook zou doen.
- U neemt contact op met de onderzoeker in deze specifieke situaties:
    - Uw kind krijgt een **loopoor**. In dit geval moet u **direct stoppen** met het gebruik van de pijnstillende oordruppels in dat oor.
    - Uw kind wordt in een ziekenhuis opgenomen of behandeld.
    - U en/of uw kind wilt niet meer meedoen met het onderzoek.
    - Uw telefoonnummer, adres of e-mailadres verandert.

6. Van welke bijwerkingen, nadelige effecten of ongemakken kunt u last krijgen?

Alle werkzame medicijnen kunnen bijwerkingen hebben. In de bijsluiter van de oordruppels staat dat er een kleine kans is op een overgevoeligheidsreactie. Deze bijwerking komt zelden voor (bij hooguit 1 op de 1.000 personen). We adviseren u dan om direct contact op te nemen met een (huis)arts.
In de bijsluiter van de oordruppels staat verder vermeld dat deze medicatie niet gebruikt dient te worden bij een loopoor of perforatie (gaatje/scheurtje) van het trommelvlies. Uw kind kan niet deelnemen aan het onderzoek als dit het geval is. Zowel de huisarts als de onderzoeker controleert dit aan het begin van het onderzoek door in de oren van uw kind te kijken.

Indien uw kind tijdens het onderzoek een **loopoor** krijgt mag uw kind de oordruppels **niet meer gebruiken**. Neem in dat geval contact op met de onderzoeker.

Doet u mee aan het onderzoek? Dan krijgt u de bijsluiter mee bij het middel.

7. Wat zijn de voordelen en de nadelen als u meedoet aan het onderzoek?

Meedoen aan het onderzoek kan voordelen en nadelen hebben. Hieronder zetten we ze op een rij. Denk hier goed over na, en praat erover met anderen.

For peer review only - http://bmjopen.bmj.com/site/about/guidelines.xhtml

Optima
pijnstillende oordruppels bij otitis media acuta

UMC Utrecht

Meedoen aan het onderzoek kan deze voordelen hebben:
De oordruppels kunnen mogelijk de mate van oorpijn van uw kind en het gebruik van antibiotica verminderen, maar zeker is dit niet.

Meedoen aan het onderzoek kan deze nadelen hebben:

- Uw kind kan last krijgen van bijwerkingen of nadelige effecten van de oordruppels, zoals beschreven in paragraaf 6.
- Meedoen aan het onderzoek kost u extra tijd.
- U moet zich houden aan de afspraken die horen bij het onderzoek.

Wilt u niet meedoen?
U beslist zelf of u met uw kind meedoet aan dit onderzoek. Deelname is geheel vrijwillig. Als u besluit niet mee te doen, hoeft u verder niets te doen. U hoeft niets te tekenen. U hoeft ook niet te zeggen waarom u niet wilt meedoen als u dat niet wilt. Uw kind krijgt dan gewoon de begeleiding en behandeling die hij/zij anders ook zou krijgen van uw huisarts.
Indien u wel meedoet, kunt u, of kan uw kind, zich op ieder moment van de studie bedenken en toch stoppen zonder de reden te melden. Dit zal geen gevolgen hebben voor de behandeling van uw kind.

8. Verzet van uw kind

Het kan gebeuren dat uw kind zich op een bepaald moment tijdens het onderzoek verzet (niet meewerkt). Indien u merkt dat dit het geval is, neem dan direct contact op met de onderzoeker. De onderzoeker moet het onderzoek dan direct stoppen. Het is moeilijk om precies te omschrijven wat verzet is. Voor de start van het onderzoek overleggen we met u wat wij zien als verzet.
De onderzoeker zal zich houden aan de Gedragscode 'Verzet van minderjarigen die deelnemen aan medisch-wetenschappelijk onderzoek' die door het NVK is vastgesteld.

9. Wanneer stopt het onderzoek?

De onderzoeker laat het u weten als er nieuwe informatie over het onderzoek komt die belangrijk voor u is. De onderzoeker vraagt u daarna of u blijft meedoen.

In deze situaties stopt voor u het onderzoek:

- Alle onderzoeken volgens het schema zijn voorbij.
- Het einde van het hele onderzoek is bereikt (na 4 weken)
- U of uw kind wilt zelf stoppen met het onderzoek. Dat mag op ieder moment. Meld dit dan meteen bij de onderzoeker. U hoeft er niet bij te vertellen waarom u stopt. Uw kind krijgt dan weer de gewone behandeling voor de middenoorontsteking. De onderzoeker zal u mogelijk nog wel uitnodigen voor een nagesprek.

For peer review only

For peer review only - http://bmjopen.bmj.com/site/about/guidelines.xhtml

Optima
pijnstillende oordruppels bij otitis media acuta

UMC Utrecht

- De onderzoeker vindt het beter voor u om te stoppen. De onderzoeker zal u mogelijk nog wel uitnodigen voor een nagesprek.
- Een van de volgende instanties besluit dat het onderzoek moet stoppen:
  - UMC Utrecht
  - de overheid, of
  - de medisch-ethische commissie die het onderzoek beoordeelt.

Wat gebeurt er als u stopt met het onderzoek?
De onderzoekers gebruiken de gegevens die tot het moment van stoppen zijn verzameld.

Het hele onderzoek is afgelopen als alle deelnemers klaar zijn.

10. Wat gebeurt er na het onderzoek?

Krijgt u de resultaten van het onderzoek?
Na afloop van de hele studie, als de gegevens van alle 300 kinderen zijn verzameld en verwerkt, ontvangt u van ons bericht over de belangrijkste uitkomsten van het onderzoek.

11. Wat doen we met de gegevens van uw kind?

Doet u mee met het onderzoek? Dan geeft u ook toestemming om de gegevens van uw kind te verzamelen, gebruiken en bewaren.

Welke gegevens bewaren we?
We bewaren deze gegevens:
- naam, geslacht en geboortedatum van uw kind
- adres, telefoonnummer en emailadres
- gegevens over gezondheid van uw kind
- (medische) gegevens die we tijdens het onderzoek verzamelen

Waarom verzamelen, gebruiken en bewaren we de gegevens van uw kind?
We verzamelen, gebruiken en bewaren de gegevens van uw kind om de vragen van dit onderzoek te kunnen beantwoorden. En om de resultaten te kunnen publiceren.

Hoe beschermen we de privacy van u en uw kind?
Om de privacy te beschermen geven wij de gegevens een code. Op de gegevens zetten we alleen deze code. De sleutel van de code bewaren we op een beveiligde plek in het UMC Utrecht. Als we de gegevens van uw kind verwerken, gebruiken we steeds alleen die code. Ook in rapporten en publicaties over het onderzoek kan niemand terughalen dat het over uw kind ging.

Wie kunnen de gegevens van uw kind zien?

For peer review only

For peer review only - http://bmjopen.bmj.com/site/about/guidelines.xhtml

Optima
pijnstillende oordruppels bij otitis media acuta

UMC Utrecht

Sommige personen kunnen wel de naam en andere persoonlijke gegevens van uw kind zonder code inzien. Dit zijn mensen die controleren of de onderzoekers het onderzoek goed en betrouwbaar uitvoeren. Deze personen kunnen bij de gegevens van uw kind komen:

- Een controleur die voor het UMC Utrecht werkt.
- Nationale toezichthoudende autoriteiten. Bijvoorbeeld de Inspectie Gezondheidszorg en Jeugd.

Deze personen houden de gegevens van uw kind geheim. Wij vragen u voor deze inzage toestemming te geven.

Hoelang bewaren we de gegevens van uw kind?
We bewaren de gegevens van uw kind 25 jaar.

Mogen we de gegevens van uw kind gebruiken voor ander onderzoek?
De gegevens van uw kind kunnen na afloop van dit onderzoek ook nog van belang zijn voor ander wetenschappelijk onderzoek op het gebied van acute middenoorontsteking. Daarvoor zullen de gegevens van uw kind 25 jaar worden bewaard in het onderzoekscentrum. In het toestemmingformulier geeft u aan of u dit goed vindt. Geeft u geen toestemming? Dan kunt u nog steeds meedoen met dit onderzoek. Uw kind krijgt dezelfde zorg.

Kunt u uw toestemming voor het gebruik van de gegevens van uw kind weer intrekken?
U kunt uw toestemming voor het gebruik van de gegevens van uw kind op ieder moment intrekken. Dit geldt voor het gebruik in dit onderzoek en voor het gebruik in ander onderzoek. Maar let op: trekt u uw toestemming in, en hebben onderzoekers dan al gegevens verzameld voor een onderzoek? Dan mogen zij deze gegevens nog wel gebruiken.

Mogelijke sturen we de gegevens van uw kind gecodeerd naar landen buiten de Europese Unie
In dit onderzoek kan het zijn dat we de gecodeerde gegevens van uw kind ook naar landen buiten de Europese Unie sturen. In die landen gelden niet de privacyregels van de Europese Unie. Maar de privacy van uw kind zal op een gelijkwaardig niveau worden beschermd.

Wilt u meer weten over de privacy van uw kind?

- Wilt u meer weten over de rechten van uw kind bij de verwerking van persoonsgegevens? Kijk dan op www.autoriteitpersoonsgegevens.nl.
- Heeft u vragen over de rechten van uw kind? Of heeft u een klacht over de verwerking van de persoonsgegevens van uw kind? Neem dan contact op met degene die verantwoordelijk is voor de verwerking van de persoonsgegevens van uw kind. Voor uw onderzoek is dat:
    - Het UMC Utrecht. Zie bijlage A voor contactgegevens, en website.
- Als u klachten heeft over de verwerking van de persoonsgegevens van uw kind, raden we u aan om deze eerst te bespreken met het onderzoeksteam. U kunt ook naar de

For peer review only - http://bmjopen.bmj.com/site/about/guidelines.xhtml

Optima
pijnstillende oordruppels bij otitis media acuta

UMC Utrecht

Functionaris Gegevensbescherming van het UMC Utrecht gaan. Of u dient een klacht in bij de Autoriteit Persoonsgegevens.

Waar vindt u meer informatie over het onderzoek?
Op de volgende website vindt u meer informatie over het onderzoek: https://www.trialregister.nl/trial/9500. Na het onderzoek kan de website een samenvatting van de resultaten van dit onderzoek tonen. U vindt het onderzoek door te zoeken op 'NL9500'.

12. Krijgt u een vergoeding als uw kind meedoet aan het onderzoek?

Aan het onderzoek zijn voor u geen kosten verbonden. Er is geen vergoeding beschikbaar voor deelname, na afloop van het huisbezoek krijgt uw kind wel een klein cadeautje.

13. Bent u verzekerd tijdens het onderzoek?

Voor iedereen die meedoet aan dit onderzoek is een verzekering afgesloten. De verzekering betaalt voor schade door het onderzoek. Maar niet voor alle schade. In **bijlage B** vindt u meer informatie over de verzekering en de uitzonderingen. Daar staat ook aan wie u schade kunt melden.

14. We informeren de huisarts en apotheker van uw kind

De onderzoeker stuurt de huisarts en apotheker van uw kind een bericht om te laten weten dat u meedoet aan het onderzoek. Daarnaast vragen we de apotheek naar bekende allergieën. Dit is voor de eigen veiligheid van uw kind.

15. Heeft u vragen?

Vragen over het onderzoek kunt u stellen aan het onderzoeksteam. Wilt u advies van iemand die er geen belang bij heeft? Dan kunt u terecht bij een onafhankelijke deskundige. Voor ons onderzoek is dat dr. P.C.J.L. Bruijning-Verhagen (zie bijlage A voor contactgegevens). Zij weet veel over het onderzoek, maar werkt niet mee aan dit onderzoek.
Heeft u een klacht? Bespreek dit dan met de onderzoeker of de arts die u behandelt. Wilt u dit liever niet? Ga dan naar Klachtenbemiddeling van het UMC Utrecht. In bijlage A staat waar u die kunt vinden.

16. Hoe geeft u toestemming voor het onderzoek?

U kunt eerst rustig nadenken over dit onderzoek. Daarna vertelt u de onderzoeker of u de informatie begrijpt en of u wel of niet wilt meedoen met uw kind. Wilt u met uw kind meedoen? Dan vult u het toestemmingsformulier in dat u bij deze informatiebrief vindt. U en de onderzoeker krijgen allebei een getekende versie van deze toestemmingsverklaring.

Dank voor uw tijd.

For peer review only - http://bmjopen.bmj.com/site/about/guidelines.xhtml

Met vriendelijke groet,

Drs. Joline de Sévaux, arts-onderzoeker, mede namens OPTIMA onderzoeksteam:

Dr. Roderick P Venekamp, huisarts

Professor dr. Roger AMJ Damoiseaux, huisarts

Professor dr. Anne GM Schilder, KNO-arts

Drs. Saskia Hullegie, arts-onderzoeker

17. Bijlagen bij deze informatie

A. Contactgegevens

B. Informatie over de verzekering

C. Toestemmingsformulier

D. Procedure informeren en toestemming geven (informed consent)

For peer review only

For peer review only - http://bmjopen.bmj.com/site/about/guidelines.xhtml

Bijlage A: contactgegevens voor UMC Utrecht

<u>Contactpersoon onderzoeksteam:</u>
Naam: J.L.H. de Sévaux, arts-onderzoeker
Julius Centrum voor Gezondheidswetenschappen en Eerstelijnsgeneeskunde, Afdeling Huisartsgeneeskunde
Universitair Medisch Centrum Utrecht
e-mail: optima@umcutrecht.nl
tel. nummer: 0625710099
Bereikbaar: dagelijks tijdens kantooruren.

<u>Onafhankelijk deskundige:</u>
Naam: Dr. P.C.J.L. Bruijning-Verhagen, kinderarts
UMC Utrecht
email: p.bruijning@umcutrecht.nl
tel. nummer: 088 75 681 81

<u>Klachten:</u>
Als u klachten heeft kunt u dit melden aan de onderzoeker of aan uw behandelend arts. Mocht u ontevreden zijn over de gang van zaken bij het onderzoek en een klacht willen indienen dan kunt u contact opnemen met de klachtenbemiddelaars. Deze zijn bereikbaar via tel. +31 88 755 62 08. Of digitaal via: https://www.umcutrecht.nl/nl/een-klacht-indienen

Functionaris voor de Gegevensbescherming van de instelling: privacy@umcutrecht.nl

Raadpleeg de website van het UMC Utrecht voor meer informatie over uw rechten: https://www.umcutrecht.nl/nl/ziekenhuis/privacy

For peer review only

For peer review only - http://bmjopen.bmj.com/site/about/guidelines.xhtml

Bijlage B: informatie over de verzekering

UMC Utrecht heeft een verzekering afgesloten voor iedereen die meedoet aan het onderzoek. De verzekering betaalt de schade die u heeft doordat u aan het onderzoek meedeed. Het gaat om schade die u krijgt tijdens het onderzoek, of binnen 4 jaar na het onderzoek. U moet schade binnen 4 jaar melden bij de verzekeraar.

Heeft u schade door het onderzoek? Meld dit dan telefonisch of per post bij deze verzekeraar:

De verzekeraar van het onderzoek is:	
Naam:	CNA Insurance Company Ltd
Adres:	Polarisavenue 140, 2132 JX Hoofddorp
Telefoonnummer:	020 57 37 274
Polisnummer:	10201366
Contactpersoon:	Mw. Esther van Herk

De verzekering betaalt maximaal € 650.000 per persoon en € 5.000.000 voor het hele onderzoek en € 7.500.000 per jaar voor alle onderzoeken van dezelfde opdrachtgever (het UMC Utrecht).

Let op: de verzekering dekt de volgende schade **niet**:

- Schade door een risico waarover we u informatie hebben gegeven in deze brief. Maar dit geldt niet als het risico groter bleek te zijn dan we van tevoren dachten. Of als het risico heel onwaarschijnlijk was.
- Schade aan uw gezondheid die ook zou zijn ontstaan als u niet aan het onderzoek had meegedaan.
- Schade die ontstaat doordat u aanwijzingen of instructies niet of niet goed opvolgde.
- Schade aan de gezondheid van uw kinderen of kleinkinderen.
- Schade door een behandelmethode die al bestaat. Of door onderzoek naar een behandelmethode die al bestaat.

Deze bepalingen staan in het 'Besluit verplichte verzekering bij medisch-wetenschappelijk onderzoek met mensen 2015'. Dit besluit staat in de Wettenbank van de overheid (https://wetten.overheid.nl).

For peer review only - http://bmjopen.bmj.com/site/about/guidelines.xhtml

Optima
pijnstillende oordruppels bij otitis media acuta

UMC Utrecht

Bijlage C: Toestemmingsformulier ouders of voogd

Behorende bij de **OPTIMA-studie (pijnstillende oordruppels bij acute middenoorontsteking)**

Ik ben gevraagd om toestemming te geven voor deelname van mijn kind aan dit medisch-wetenschappelijke onderzoek:

Naam proefpersoon (kind): …………………….. Geboortedatum: ___ / ___ / _____

- Ik heb de informatiebrief voor de ouders/verzorgers gelezen. Ook kon ik vragen stellen. Mijn vragen zijn goed genoeg beantwoord. Ik had genoeg tijd om te beslissen of ik wil dat mijn kind meedoet.
- Ik weet dat meedoen vrijwillig is. Ook weet ik dat ik op ieder moment kan beslissen dat mijn kind toch niet meedoet. Ik hoef dan niet te zeggen waarom ik dat wil.
- Ik geef de onderzoeker toestemming om mijn huisarts en apotheker te laten weten dat mijn kind meedoet aan dit onderzoek.
- Ik geef de onderzoekers toestemming om de gegevens van mijn kind te verzamelen en te gebruiken. De onderzoekers doen dit alleen om de onderzoeksvraag in dit onderzoek te beantwoorden.
- Ik weet dat voor de controle van het onderzoek sommige mensen toegang tot alle gegevens van mijn kind kunnen krijgen. Die mensen staan in deze informatiebrief. Ik geef deze mensen toestemming om de gegevens van mijn kind in te zien voor deze controle.
- Ik weet dat mijn gecodeerde gegevens naar landen buiten de EU kunnen worden gestuurd waar privacyregels van de EU niet gelden.
- Wilt u in de tabel hieronder ja of nee aankruisen?

Ik geef toestemming om mij na dit onderzoek te benaderen voor een interview over uw ervaringen met betrekking tot de studiedeelname en de behandeling.	Ja ☐	Nee☐
Ik geef toestemming om mijn kind na dit onderzoek te vragen of hij/zij wil meedoen met een vervolgonderzoek.	Ja ☐	Nee☐
Ik geef toestemming om de gegevens van mijn kind te bewaren om dit te gebruiken voor ander onderzoek, zoals in de informatiebrief staat.	Ja ☐	Nee☐

- Ik ga ermee akkoord dat mijn kind meedoet aan dit onderzoek.

For peer review only

For peer review only - http://bmjopen.bmj.com/site/about/guidelines.xhtml

Optima
pijnstillende oordruppels bij otitis media acuta

UMC Utrecht

Naam ouder/voogd**: ……………………………….

Handtekening: …………………………………. Datum: ___ / ___ / _____

Naam andere ouder/voogd**: …………………….

Handtekening: ……………………………………… Datum: ___ / ___ / _____

Ik verklaar dat ik de persoon/personen hierboven volledig heb geïnformeerd over het genoemde onderzoek.

Wordt er tijdens het onderzoek informatie bekend die de toestemming van de ouder of voogd kan beïnvloeden? Dan laat ik dit op tijd aan hem/haar weten.

Naam onderzoeker (of diens vertegenwoordiger): …………………………

Handtekening: ……………………… Datum: ___ / ___ / _____

----

* Doorhalen wat niet van toepassing is.

** Als het kind jonger dan 16 jaar is, ondertekenen de ouders die het gezag uitoefenen of de voogd dit formulier. Kinderen van 12 t/m 15 jaar die zelfstandig beslissingen kunnen nemen (wilsbekwaam zijn), moeten daarnaast zelf een formulier ondertekenen

De ouder/voogd krijgt een volledige informatiebrief mee, samen met een getekende versie van het toestemmingsformulier.

For peer review only - http://bmjopen.bmj.com/site/about/guidelines.xhtml

Bijlage D: Procedure informeren en toestemming geven (informed consent)

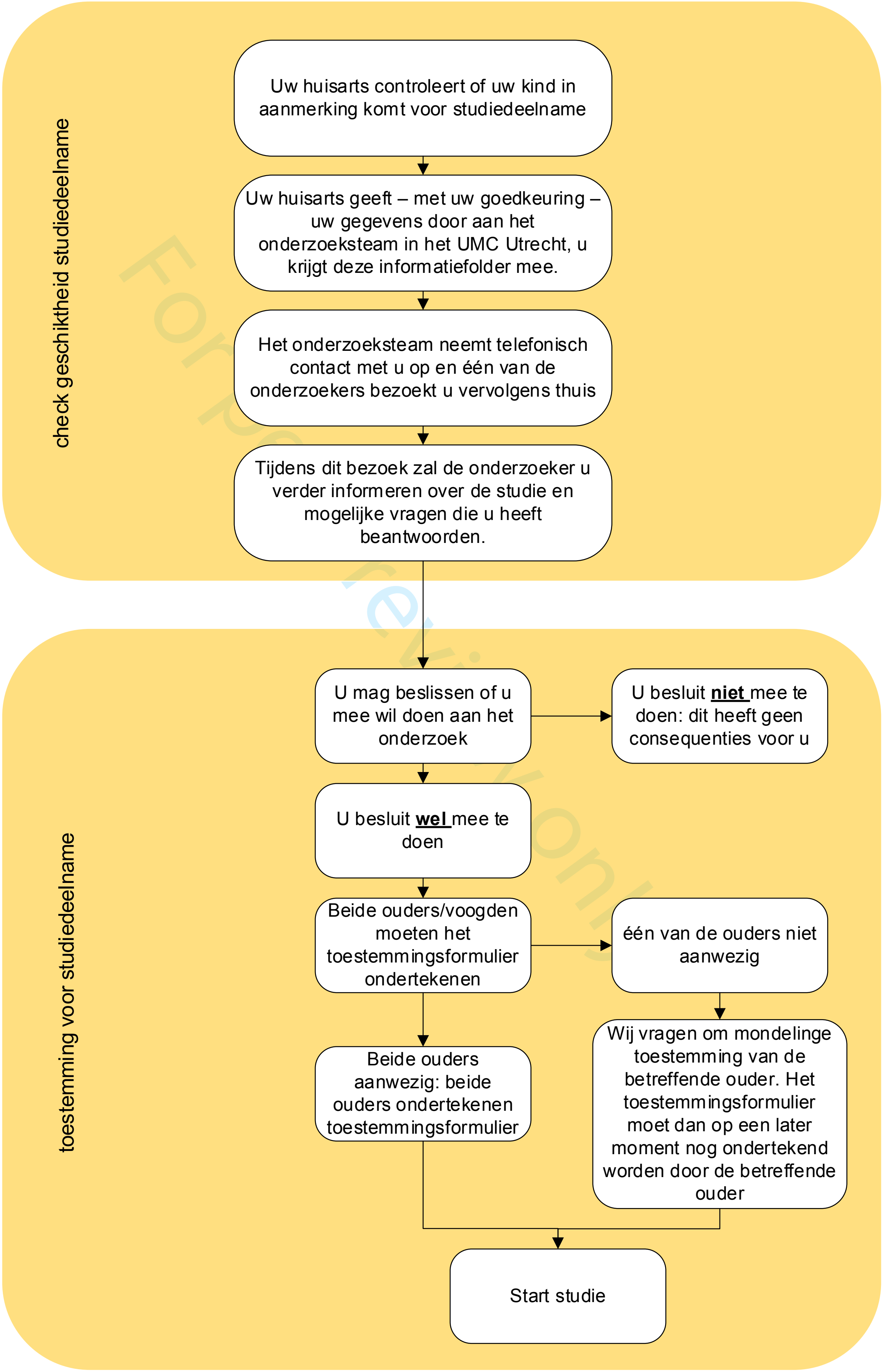

For peer review only - http://bmjopen.bmj.com/site/about/guidelines.xhtml

BMJ Open

Effectiveness of analgesic ear drops as add-on treatment to oral analgesics in children with acute otitis media: study protocol of the OPTIMA pragmatic randomised controlled trial

Journal:	*BMJ Open*
Manuscript ID	bmjopen-2022-062071.R1
Article Type:	Protocol
Date Submitted by the Author:	21-Sep-2022
Complete List of Authors:	de Sévaux, Joline; University Medical Centre Utrecht, Julius Center for Health Sciences and Primary Care<br>Damoiseaux, Roger; University Medical Centre Utrecht, Julius Center for Health Sciences and Primary Care<br>Hullegie, Saskia; University Medical Centre Utrecht, Julius Center for Health Sciences and Primary Care<br>Sanders, Elisabeth ; Wilhelmina Children's Hospital University Medical Centre, Department of Paediatric Immunology and Infectious Diseases; National Institute for Public Health and the Environment, Centre for Infectious Disease Control<br>Wit, Ardine; University Medical Centre Utrecht, Julius Center for Health Sciences and Primary Care; National Institute for Public Health and the Environment, Centre for Nutrition, Prevention and Healthcare<br>Zuithoff, Nicolaas; University Medical Centre Utrecht, Julius Center for Health Sciences and Primary Care<br>Yardley, Lucy; University of Southampton, School of Psychology, Faculty of Environmental and Life Sciences; University of Bristol, School of Psychological Science, Faculty of Life Sciences<br>Anthierens , Sibyl ; University of Antwerp Faculty of Medicine and Health Sciences, Department of Family Medicine and Population Health<br>Little, Paul; University of Southampton, Aldermoor Health Centre, Primary Care Research Centre, Primary Care Population Sciences and Medical Education, Faculty of Medicine<br>Hay, Alastair; University of Bristol, Centre for Academic Primary Care, Bristol medical School: Population Health Sciences<br>Schilder, Anne; University Medical Centre Utrecht, Julius Center for Health Sciences and Primary Care; NIHR University College London Hospitals Biomedical Research Centre<br>Venekamp , Roderick ; University Medical Centre Utrecht, Julius Center for Health Sciences and Primary Care
**Primary Subject Heading**:	General practice / Family practice
Secondary Subject Heading:	Ear, nose and throat/otolaryngology, Epidemiology, Evidence based practice, Infectious diseases, Qualitative research
Keywords:	PRIMARY CARE, Epidemiology < INFECTIOUS DISEASES, Paediatric

	otolaryngology < OTOLARYNGOLOGY, Paediatric infectious disease & immunisation < PAEDIATRICS, PAIN MANAGEMENT, QUALITATIVE RESEARCH

SCHOLARONE™
Manuscripts

Effectiveness of analgesic ear drops as add-on treatment to oral analgesics in children with acute otitis media: study protocol of the OPTIMA pragmatic randomised controlled trial

Joline LH de Sévaux[1], Roger AMJ Damoiseaux[1], Saskia Hullegie[1], Elisabeth AM Sanders[2,3], G Ardine de Wit[1,4], Nicolaas P A Zuithoff[1], Lucy Yardley[5,6], Sibyl Anthierens[7], Paul Little[8], Alastair D Hay[9], Anne GM Schilder[1,10,11], Roderick P Venekamp[1]

[1] Julius Center for Health Sciences and Primary Care, University Medical Center Utrecht, Utrecht University, Utrecht, The Netherlands

[2] Department of Paediatric Immunology and Infectious Diseases, Wilhelmina Children's Hospital, University Medical Center Utrecht, Utrecht University, Utrecht, The Netherlands

[3] Centre for Infectious Disease Control, National Institute of Public Health and the Environment (RIVM), Bilthoven, The Netherlands

[4] Centre for Nutrition, Prevention and Healthcare, National Institute of Public Health and the Environment (RIVM), Bilthoven, the Netherlands

[5]School of Psychological Science, Faculty of Life Sciences, University of Bristol, Bristol, United Kingdom

[6] School of Psychology, Faculty of Environmental and Life Sciences, University of Southampton, Southampton, United Kingdom

[7] Department of Family Medicine and Population Health, Faculty of Medicine and Health Sciences, University of Antwerp, Antwerp, Belgium

[8] Primary Care Research Centre, Primary Care Population Sciences and Medical Education, Faculty of Medicine, University of Southampton, Aldermoor Health CentreSouthampton, United Kingdom

[9] Centre for Academic Primary Care, Bristol medical School: Population Health Sciences, University of Bristol, Bristol, United Kingdom

[10] National Institute for Health Research University College London Hospitals Biomedical Research Centre

[11] evidENT, Ear Institute, University College London, London, United Kingdom

For peer review only

Corresponding author

Joline LH de Sévaux
Julius Center for Health Sciences and Primary Care
University Medical Center Utrecht
Str. 6.131, P.O. Box 85500, 3508 GA Utrecht, the Netherlands
Tel: +31 6 25 71 00 99
Email: j.l.h.desevaux-2@umcutrecht.nl

For peer review only - http://bmjopen.bmj.com/site/about/guidelines.xhtml

ABSTRACT

Introduction

Ear pain is the most prominent symptom of childhood acute otitis media (AOM). To control the pain and reduce reliance on antibiotics, evidence of effectiveness for alternative interventions is urgently needed. This trial aims to investigate whether analgesic ear drops added to usual care provide superior ear pain relief over usual care alone in children presenting to primary care with AOM.

Methods and analysis

This is a pragmatic, two arm, individually randomised, open, superiority trial with cost-effectiveness analysis and nested mixed-methods process evaluation in general practices in the Netherlands. We aim to recruit 300 children aged 1-6 years with a general practitioner (GP)-diagnosis of AOM and ear pain. Children will be randomly allocated (ratio 1:1) to either 1) lidocaine hydrochloride 5mg/g ear drops (Otalgan) 1-2 drops up to six times daily for a maximum of 7 days in addition to usual care (oral analgesics, with/without antibiotics) or 2) usual care. Parents will complete a symptom diary for 4 weeks as well as generic and disease-specific quality of life questionnaires at baseline and 4 weeks. The primary outcome is the parent-reported ear pain score (0-10) over the first 3 days. Secondary outcomes include proportion of children consuming antibiotics, oral analgesic use and overall symptom burden in the first 7 days; number of days with ear pain, number of GP reconsultations and subsequent antibiotic prescribing, adverse events, complications of AOM and cost-effectiveness during 4-week follow-up; generic and disease-specific quality of life at 4 weeks; parents' and GPs' views and experiences with treatment acceptability, usability and satisfaction.

Ethics and dissemination

The Medical Research Ethics Committee Utrecht, the Netherlands, has approved the protocol (21-447/G-D). All parents/guardians of participants will provide written informed consent. Study results will be submitted for publication in peer reviewed medical journals and presented at relevant (inter)national scientific meetings.

Trial registration: The Netherlands Trial Register; NL9500. Date of registration: 28 May 2021.

Word count: 3591 (excluding title page, abstract, references, figures and boxes).

Keywords: acute otitis media; children, primary care; analgesic ear drops; randomised controlled trial.

For peer review only - http://bmjopen.bmj.com/site/about/guidelines.xhtml

ARTICLE SUMMARY

Strengths

- The pragmatic, open design enhances the applicability of the trial findings in daily practice.
- The pragmatic, open design is most suited to address the key secondary outcomes of the impact of the analgesic ear drops on the proportion of children consuming antibiotics and its cost-effectiveness in everyday practice.

Limitations

- The combination of the open design and the subjective, parent-reported outcomes does not control for the placebo effects, but will more closely reflect the benefits that would accrue in everyday practice with regard to analgesic effects.
- Contamination is possible given the over-the-counter availability of analgesic eardrops.

For peer review only

INTRODUCTION

Although the current COVID-19 pandemic has a profound influence on the incidence of acute otitis media (AOM) in children[1-3], it will remain an important cause of primary care consultations.[4 5] Ear pain is the predominant symptom and central to children's and parents' experience of the condition.[6 7] High-quality studies have shown that most symptoms of AOM settle within a few days without antibiotics in otherwise healthy children living in high-income countries[8], but it may take eight days for ear pain to fully resolve in 90% of the children.[9] Globally, most AOM clinical practice guidelines therefore recommend oral analgesics in all, and antibiotics in selected, children.[10] However, antibiotics are frequently prescribed in children with AOM[4 5] leading to side effects in at least 1 out of 10 children and emergence of antimicrobial resistance.[11 12] Evidence of effectiveness of management options to relieve the most distressing symptom of AOM and reduce reliance on antibiotics is therefore urgently needed.[13]

Recently, we found that a general practitioner (GP)-targeted educational intervention to improve pain management in children with AOM resulted in an increase in oral analgesic use, in particular ibuprofen, but failed to reduce parent-reported ear pain and antibiotic use.[14] Therefore, alternative interventions need further exploration. Analgesic ear drops, widely available, cheap and safe, may be well-suited to fill this gap. When applied into the external auditory canal, these agents could relieve pain and distress by blocking nerve conduction of the tympanic membrane.[15] So far, evidence on their use in children with AOM is limited.[15 16] Our trial aims to produce this important evidence.

OBJECTIVE

The primary aim of this pragmatic randomised controlled trial is to investigate whether analgesic ear drops added to usual care (oral analgesics with/without antibiotics) provide superior ear pain relief over usual care alone in children presenting to primary care with AOM and ear pain.

For peer review only - http://bmjopen.bmj.com/site/about/guidelines.xhtml

METHODS AND ANALYSIS

Study design and setting

A pragmatic, two arm, individually randomised, open, superiority trial with cost-effectiveness analysis in general practices in the Netherlands with a follow-up of 4 weeks will be performed. Potential trial participants will be recruited from primary care (approximately from 150 GPs) in the region of Utrecht, The Netherlands. A mixed-method process evaluation will be nested within the trial (see Supplemental Material 1).[17-22] This process evaluation will capture data to understand how participating in the trial has an impact on the consultation and behaviour and how AOM interventions (including analgesic ear drops, antibiotics and oral analgesics) are viewed by parents and GPs. The process evaluation consists of a survey with brief statements measuring attitudes of all parents based on the extended Common Sense Model[17] and a three-item Consultation Satisfaction Questionnaire,[18] and a qualitative part (semi-structured interviews capturing parents and GP's views and experiences, respectively).

Participants

Children aged 1-6 years presenting to primary care with AOM[23] and parent-reported ear pain in 24 hours prior to enrolment will be eligible for trial participation. Children with (suspected) tympanic membrane perforation or ventilation tube (as they carry a risk of inner ear damage causing hearing loss or tinnitus after application of lidocaine drops), ear wax obscuring visualisation of the tympanic membrane or those who are very unwell or require hospitalisation will be excluded. Detailed inclusion and exclusion criteria are listed in Box 1.

For peer review only

Box 1. Full list of inclusion and exclusion criteria

Inclusion criteria:

- Age 1 to 6 years
- Parent-reported ear pain in 24 hours prior to enrolment
- GP-diagnosis of (uni- or bilateral) AOM

Exclusion criteria:

Children:

- with (suspected) tympanic membrane perforation or ventilation tubes
- with ear wax obscuring visualisation of the tympanic membrane
- who are systemically very unwell or require hospital admission (e.g. child has signs and symptoms of serious illness and/or complications such as mastoiditis/meningitis)
- who are at high risk of serious complications including children with known immunodeficiency other than partial IgA or IgG2 deficiencies, craniofacial malformation including cleft palate, Down syndrome and previous ear surgery (with the exception of ventilation tubes in the past)
- who have a known allergy or sensitivity to study medication or similar substances (e.g. other amide-type anaesthetics: bupivacaine, mepivacaine, prilocaine, etc)
- who have taken part in any research involving medicines within the last 90 days, or any other AOM-related research within the last 30 days
- who suffer from chronic recurrent pain of another origin than the ear
- who have participated in this trial during prior AOM episode

Study procedures and data collection

Recruitment and inclusion of participants

Figure 1 depicts a flow diagram of the study procedures. During regular primary care consultations, participating GPs will inform parents of potentially eligible children about the trial. Next, GPs will ask parents who are interested in participation for consent to provide their contact details to the study team at the University Medical Center (UMC) Utrecht. Members of the team will contact the parents by phone the same day to provide detailed information about the trial. If parents provisionally agree to participate and if the child fulfils the trial eligibility criteria, the study physician will visit the child at home. At this home visit, written informed consent for participation in the trial and the semi-structured interview will be obtained (Supplemental Material 2), baseline demographic and disease-specific data will be collected and a physical examination including otoscopy will be performed. Parents are also asked to complete a generic and disease-

For peer review only - http://bmjopen.bmj.com/site/about/guidelines.xhtml

specific quality of life questionnaires on behalf of their child. A full description of the nested process evaluation is given in Supplemental Material 1

Study group assignment

At the conclusion of the home visit, a trial randomisation website will be accessed for concealed study-group assignment. An independent data manager generates a computer-generated randomisation sequence stratified for age (<2 years vs 2 years and above), AOM laterality (unilateral vs bilateral), and AOM baseline antibiotic prescribing (yes vs no). Children will be randomly allocated to either 1) lidocaine hydrochloride 5mg/g (Otalgan) ear drops 1-2 drops up to six times daily for a maximum of 7 days[24 25] with usual care (oral analgesics, with/without antibiotics) or 2) usual care. Analgesic eardrops will be provided by the study team. These drops should not be used in children with a tympanic membrane perforation or ventilation tube as they carry a risk of inner ear damage causing hearing loss or tinnitus. These children will therefore be excluded, as well as those with ear wax obscuring visualisation of the tympanic membrane. Parents of children allocated to the intervention group are expressly instructed to stop applying the drops if their child develops ear discharge. The study team will notify the GP about the result of the randomisation. The local pharmacist will be notified in case the child is allocated to the analgesic ear drops, in order to check any known sensitivities/allergies and any interactions (with current or future drugs). Any treatment decisions other than the use of analgesic ear drops, i.e. antibiotics and oral analgesics, will be left to the GP's discretion in both groups. To those allocated to the intervention group, the study physician will not provide any treatment advice other than instructions about the use of analgesic ear drops. To those allocated to the control group, the study physician will not provide any treatment advice.

During follow-up parents and GPs will be encouraged to manage AOM symptoms according to current clinical practice guidance on AOM in children issued by the Dutch College of General Practitioners. Lidocaine eardrops are currently not recommended in this guideline due to the lack of evidence.[23]

Follow-up data collection

Participants will be followed for 4 weeks. A telephone call will be scheduled at day 3 which will allow us to capture critical data on our primary outcome. Parents will keep a diary of AOM-related symptoms including ear pain scores and fever recordings, treatment adherence, prescribed and over-the-counter (OTC) medication use,

For peer review only - http://bmjopen.bmj.com/site/about/guidelines.xhtml

AOM-related health care resource use, child care and travel costs, adverse events and complications of AOM for 4 weeks. At the end of follow-up, parents will complete a questionnaire on parental productivity losses and generic and disease-specific quality of life questionnaires. The diary and questionnaires can either be completed online or on paper forms (see data management). The study team will contact the parents by phone to check if all data is collected. During this phone call, an appointment for the semi-structured interview can be made with parents who are still willing to participate in the nested qualitative investigation.

Validated questionnaires used in the trial

- Child's ear pain intensity will be reported by parents in the symptom diary using a 0-10 numerical rating scale which has been validated for measuring pain in children with AOM.[26]
- Child's symptoms of crying/distress, disturbed sleep, interference with normal activity, appetite, fever and hearing problems will be reported by parents in the symptom diary using a 0-6 Likert scale with higher scores indicating greater severity. This scale has been proven valid and was successfully used in our previous trials.[16 26]
- Generic quality of life of the child at baseline and 4 weeks will be assessed using the 47-item short-form of the Infant Toddler Quality of Life Questionnaire (ITQOL-SF47) which is developed for use in infants and toddlers aged 2 months to 6 years and has shown to be a feasible instrument with adequate psychometric properties to discriminate between children with and without doctor-diagnosed respiratory disease.[27]
- Disease-specific quality of life of the child at baseline and 4 weeks will be assessed using the Otitis media-6 (OM-6) questionnaire.[28]
- Productivity losses (e.g. parental absenteeism from work) because of AOM of their child will be assessed using the IMTA Productivity Cost Questionnaire (iPCQ) at 4 weeks.[29]

Outcomes

Primary outcome

The parent-reported ear pain score over the first three days. To this end, parents will record their child's ear pain scores during the first three consecutive days using a 0-10 validated numerical rating scale.

Secondary outcomes

- proportion of children consuming antibiotics in the first 7 days;

For peer review only - http://bmjopen.bmj.com/site/about/guidelines.xhtml

- oral analgesic use in the first 7 days;
- overall symptom burden (crying/distress, disturbed sleep, interference with normal activity, appetite, fever and hearing problems) using a 0-6 Likert scale in the first 7 days;
- number of days with ear pain during follow up (4 weeks);
- number of GP reconsultations with/without subsequent antibiotic prescribing during follow-up;
- adverse events during follow-up;
- complications of AOM during follow-up;
- costs and cost-effectiveness during follow-up;
- generic quality of life of the child at 4 weeks;
- disease-specific quality of life of the child at 4 weeks;
- parents' and GPs' views of treatment acceptability, usability and satisfaction.

Sample size calculation

The main aim is to demonstrate that analgesic ear drops as added to usual care provide superior parent-reported ear pain relief over the first three days compared to usual care. In our recent trial, the mean parent-reported ear pain score (0-10 scale) over the first three days was 4.36 (SD 2.12) among those allocated to usual care.[14] Patient and Public Involvement (PPI) input from the UK suggested that a 1-point reduction (on a 0-10 scale) would be a clinically important difference.[16] However, our parent panel felt that even a somewhat smaller difference would be clinically meaningful given the low cost, low risk nature of the intervention. To detect a pain reduction of 20% (reflecting a 0.87 point reduction in pain) compared with usual care with 90% power (beta 0.1) and at a 5% significance level (alpha 0.05), 126 children per group will be needed. To allow for 20% attrition, we aim to include 300 participants.

The estimated sample size will also provide substantial power to address the key secondary outcome of the proportion of children consuming antibiotics. Anticipating 47% of children consuming antibiotics in the usual care group as with our previous trial[14], 252 children will provide 89% power (beta 0.11; alpha 0.05) to detect a 20% difference in the proportion of children consuming antibiotics.

Data management

During study conduct, we will comply with the EU General Data Protection Regulation and the Dutch Act on

For peer review only - http://bmjopen.bmj.com/site/about/guidelines.xhtml

Implementation of the General Data Protection Regulation. The data management department of the Julius Center, UMC Utrecht will be responsible for handling and storage of the trial data using a secured and coded online database (Research Online 2.0 [RO 2.0]). Data will be either entered directly into RO 2.0 or collected on paper forms first. For data entered directly into RO 2.0, processes to promote data quality are added. Coded trial data will be securely stored for 25 years in the main study folder on a central drive of the Julius Center.

Statistical analysis

All analyses will be performed according to the intention-to-treat (ITT) principle and blinded with respect to study-group assignment. The results will be reported in accordance with the CONSORT guidelines.[30]

Clinical effectiveness

Descriptive statistics will be used to assess any baseline differences between treatment groups. The primary outcome will be analysed with a linear regression model with a residual covariance (i.e. GEE type) matrix to correct for repeated measurements. In main (adjusted) analysis, treatment group, time, a priori specified prognostic factors (baseline ear pain score, duration of ear pain prior to enrolment) and stratification factors (age (<2 years vs 2 years and above), AOM laterality (unilateral vs bilateral), and baseline antibiotic prescribing (yes vs no)) will be included in the model. The validity of the model, i.e. normality and homoscedasticity, will be evaluated by assessing residuals and the comparison between treatment groups will be expressed as differences in means with 95% confidence intervals (CIs). If more than 5% of baseline and outcome data is missing, a sensitivity analysis will be performed using multiple imputation techniques.[31 32]

Secondary outcomes will be analysed using negative-binomial regression analyses for count variables, log binomial regression analyses for dichotomous variables, and linear regression analyses for continuous variables, where applicable adjusted for repeated measurements. For these analyses, the comparison between treatment groups will be expressed as rate ratios, risk ratios, and mean differences, respectively; all with 95% CIs.

We will also conduct pre-specified, exploratory subgroup analyses to investigate whether age (<2 years vs 2 years and above) or AOM laterality (unilateral vs bilateral) modify the effectiveness of analgesic ear drops. In further sensitivity analysis, we will also explore the added impact of antibiotic consumption on the primary outcome of interest.

For peer review only - http://bmjopen.bmj.com/site/about/guidelines.xhtml

Cost effectiveness analysis

Alongside the trial, a cost effectiveness analysis from a societal perspective, i.e. including both medical and non-medical costs, will be performed. The societal perspective is particularly relevant in this case, since non-medical costs are particularly relevant in AOM.[33] Those costs mainly relate to parental productivity losses and costs of additional childcare.

Costs analysis

All costs will be estimated at the patient level. Costs of medication use will be retrieved from the Dutch formulary and a pharmacist's fee will be added for every prescription.[24 34] Costs of OTC and complementary medicines will be calculated per day, based on current average retail prices. Costs of consulting a GP or a medical specialist, and costs of hospitalisations will be based on current Dutch guidelines for pharmaco-economic evaluation in health care research. Costs of diagnostic tests will also be derived from these guidelines.[34 35] Costs of surgical procedures will be retrieved from a previous Dutch costing study that calculated costs for the different components of surgical procedures.[36]

Resource use such as doctor's visits, prescribed medication (including antibiotics), specialist referrals, hospital admissions, surgical interventions as well as out-of-pocket expenses such as OTC medication, child care, and travel costs will be collected from study diaries filled out by parents. Cost prices will be estimated according to guidelines for pharmaco-economic evaluation in health care research.[34 35] Costs associated with productivity losses will be retrieved from the iPCQ questionnaire and estimated using the friction cost method.[29 37] The hourly cost estimate for childcare will be derived from the Dutch National Institute for Family Finance Information (NIBUD).[38] Travel expenses will be calculated for healthcare visits following the Dutch guideline for economic evaluation.[35] Mean costs per patient will be compared across the randomisation groups.. As the time horizon is only 4 weeks, discounting does not apply.

Cost-effectiveness results

Costs from a societal perspective will be compared with the parent-reported ear pain scores over the first three days. We will calculate incremental cost-effectiveness ratios by dividing the estimated differences in costs between groups by the differences in effects observed, i.e. the additional cost per additional 1 point reduction

For peer review only - http://bmjopen.bmj.com/site/about/guidelines.xhtml

in mean ear pain score over the first three days for the analgesic ear drops group versus the usual care group. Uncertainty will be addressed by means of two-stage non-parametric bootstrap sampling. The effect of any difference in baseline characteristics on cost-effectiveness results will be investigated using net benefit regression methods. In case of missing data, we will use multiple imputation techniques. The final results will be presented using incremental cost-effectiveness planes and cost-effectiveness acceptability curves.

Monitoring and safety

The trial will be monitored regularly by an independent research data monitor of the UMC Utrecht to ensure the quality of the trial execution. Since we do not expect any safety issues and the overall risk of this trial was judged negligible, we have not established a data safety monitoring board and refrain from conducting a safety interim analysis. However, in accordance to section 10, subsection 4, of the WMO, the sponsor will suspend the study if there is sufficient ground that continuation of the study will jeopardise subject health or safety.

ETHICS AND DISSIMINATION

The study will be conducted according to the principles of the Declaration of Helsinki (10th version, October 2013), the ICH GCP E6(R2) guideline, the Dutch Law on medicinal products (‘geneesmiddelenwet’) and in accordance with the Medical Research Involving Human Subjects Act (WMO). The Medical Research Ethics Committee Utrecht, the Netherlands, has approved the protocol (21-447/G-D).

Research findings will be disseminated through publications in peer reviewed medical journals targeted towards the wider audience of health professionals involved in the care of children and presented at relevant national and international scientific meetings. We will work with our parent panel to help interpret the findings of the trial and harness their resources for dissemination to the lay public.

PATIENT AND PUBLIC INVOLVEMENT

A panel of eight parents was established for this trial. This panel helped to shape the grant proposal and final study protocol during online focus group meetings, with particular advice given on patient-relevant outcomes including the minimally important clinical differences, recruitment strategy, and the design of data collection. The parent panel will be actively involved throughout all critical stages of the trial through regular parent panel meetings.

For peer review only

CURRENT STUDY STATUS

Study enrolment is ongoing with the first participant being included on 6 October 2021.

For peer review only

DISCUSSION

Up to now, evidence on the use of analgesic ear drops in children with AOM is limited. A 2011 systematic review included five studies of which two (117 children) compared analgesic ear drops to placebo.[15] The authors concluded that these trials provide limited evidence that a single dose of analgesic ear drops is effective in reducing ear pain 30 minutes after administration in older children (age 3 to 19 years) with AOM.[15] Since then, a UK-based trial compared at-home, repeated dosing, of analgesic ear drops with placebo ear drops and usual care. This trial was terminated prematurely due to operational issues.[16] Initial data of 84 children recruited in primary care indicate that ear pain levels were lower in both ear drops groups than in the usual care group. However, these estimated treatment effects are imprecise because of the low sample size target and therefore require confirmation in future studies.

Our trial aims to do so by assessing the clinical and cost effectiveness of analgesic eardrops in children with AOM, as well as views and experiences of parents and GPs through the nested process evaluation. The pragmatic, open design enhances the applicability of the trial findings to daily practice and is most suited to address the key secondary outcome of the impact of the analgesic ear drops on the proportion of children consuming antibiotics in everyday practice.[39] This would be much more difficult to determine realistically in a placebo-blinded study where children in both groups would receive ear drops (either placebo or analgesic drops); such ‘treatment’ may in itself lead to altered parental (health care seeking) behaviour. Additionally, the open design, in which the control group receives usual care, is most suited for ‘real world’ cost-effectiveness analysis. However, the lack of blinding in combination with the subjective, parent-reported outcomes might also lead to bias in the assessment of analgesic effects. To minimise this potential bias, the study physician will emphasize during the baseline visit that it is not known whether the drops have any effect in children with AOM. Furthermore, a recent meta-epidemiological study found no evidence for a difference in estimated treatment effect between blinded and non-blinded trials.[40] Although it is still unclear what caused the results of this study, it suggests that blinding is less important than anticipated. Our design does not control for the placebo effects, but will more closely reflect the benefits that would accrue in everyday practice with regard to analgesic effects.

Albeit not recommended in the Dutch AOM clinical practice guideline[23], the OTC availability of lidocaine eardrops may inherently incur the risk of contamination, i.e. parents in the control group buying lidocaine ear

For peer review only - http://bmjopen.bmj.com/site/about/guidelines.xhtml

drops themselves. We consider this unlikely since only 5% (10/221) of participants did use lidocaine ear drops in our previous trial of a GP-targeted educational intervention to improve pain management in children with AOM.[14] We will, however, capture data on the use of lidocaine ear drops in all trial participants to monitor potential contamination.

ACKNOWLEDGEMENTS

We gratefully thank our parent panel for their valuable input and all children and parents who participated in our trial thus far.

AUTHOR'S CONTRIBUTIONS

RPV, AGMS and RAMJD designed the trial. JLHdS, SH and RPV drafted the first version of the protocol paper. All other authors (RAMJD, AGMS, EAMS, GAdW, NPAZ, LY, SA, PL and ADH) provided feedback on this version. All authors (JLHdS, SH, RPV, RAMJD, AGMS, EAMS, GAdW, NPAZ, LY, SA, PL and ADH) approved the final version of the manuscript.

FUNDING STATEMENT

The trial is supported by a grant from the Netherlands Organisation for Health Research and Development (ZonMw– grant number 10060011910003). The funding agency had no role in the design, and will not have any role during execution of the trial, data-analyses, interpretation of the data or decision to submit results. LY is an NIHR Senior Investigator and her research programme is partly supported by NIHR Applied Research Collaboration (ARC)-West, NIHR Health Protection Research Unit (HPRU) for Behavioural Science and Evaluation, and the NIHR Southampton Biomedical Research Centre (BRC).

COMPETING INTERESTS

The authors declare that they have no conflict of interest.

For peer review only

REFERENCES

1. Hullegie S, Schilder AGM, Marchisio P, et al. A Strong Decline in the Incidence of Childhood Otitis Media During the COVID-19 Pandemic in the Netherlands. *Front Cell Infect Microbiol* 2021;11:768377. doi: 10.3389/fcimb.2021.768377 [published Online First: 2021/11/19]
2. Kaur R, Schulz S, Fuji N, et al. COVID-19 Pandemic Impact on Respiratory Infectious Diseases in Primary Care Practice in Children. *Front Pediatr* 2021;9:722483. doi: 10.3389/fped.2021.722483 [published Online First: 2021/10/01]
3. McBride JA, Eickhoff J, Wald ER. Impact of COVID-19 Quarantine and School Cancelation on Other Common Infectious Diseases. *Pediatr Infect Dis J* 2020;39(12):e449-e52. doi: 10.1097/inf.0000000000002883 [published Online First: 2020/10/09]
4. Gulliford M, Latinovic R, Charlton J, et al. Selective decrease in consultations and antibiotic prescribing for acute respiratory tract infections in UK primary care up to 2006. *J Public Health (Oxf)* 2009;31(4):512-20. doi: 10.1093/pubmed/fdp081 [published Online First: 2009/09/08]
5. van den Broek d'Obrenan J, Verheij TJ, Numans ME, et al. Antibiotic use in Dutch primary care: relation between diagnosis, consultation and treatment. *J Antimicrob Chemother* 2014;69(6):1701-7. doi: 10.1093/jac/dku005 [published Online First: 2014/02/11]
6. Barber C, Ille S, Vergison A, et al. Acute otitis media in young children - what do parents say? *Int J Pediatr Otorhinolaryngol* 2014;78(2):300-6. doi: 10.1016/j.ijporl.2013.11.030 [published Online First: 2014/01/01]
7. Hansen MP, Howlett J, Del Mar C, et al. Parents' beliefs and knowledge about the management of acute otitis media: a qualitative study. *BMC Fam Pract* 2015;16:82. doi: 10.1186/s12875-015-0297-7 [published Online First: 2015/07/08]
8. Venekamp RP, Sanders SL, Glasziou PP, et al. Antibiotics for acute otitis media in children. *Cochrane Database Syst Rev* 2015(6):CD000219. doi: 10.1002/14651858.CD000219.pub4 [published Online First: 2015/06/24]
9. Thompson M, Vodicka TA, Blair PS, et al. Duration of symptoms of respiratory tract infections in children: systematic review. *BMJ* 2013;347:f7027. doi: 10.1136/bmj.f7027 [published Online First: 2013/12/18]
10. Ovnat Tamir S, Shemesh S, Oron Y, et al. Acute otitis media guidelines in selected developed and developing countries: uniformity and diversity. *Arch Dis Child* 2017;102(5):450-57. doi: 10.1136/archdischild-2016-310729 [published Online First: 2016/09/08]
11. Costelloe C, Metcalfe C, Lovering A, et al. Effect of antibiotic prescribing in primary care on antimicrobial resistance in individual patients: systematic review and meta-analysis. *Bmj* 2010;340:c2096. doi: 10.1136/bmj.c2096 [published Online First: 2010/05/21]
12. Gillies M, Ranakusuma A, Hoffmann T, et al. Common harms from amoxicillin: a systematic review and meta-analysis of randomized placebo-controlled trials for any indication. *Cmaj* 2015;187(1):E21-e31. doi: 10.1503/cmaj.140848 [published Online First: 2014/11/19]
13. Pouwels KB, Dolk FCK, Smith DRM, et al. Actual versus 'ideal' antibiotic prescribing for common conditions in English primary care. *J Antimicrob Chemother* 2018;73(suppl_2):19-26. doi: 10.1093/jac/dkx502 [published Online First: 2018/03/01]
14. van Uum RT, Venekamp RP, Zuithoff NP, et al. Improving pain management in childhood acute otitis media in general practice: a cluster randomised controlled trial of a GP-targeted educational intervention. *Br J Gen Pract* 2020;70(699):e684-e95. doi: 10.3399/bjgp20X712589 [published Online First: 2020/08/26]
15. Foxlee R, Johansson A, Wejfalk J, et al. Topical analgesia for acute otitis media. *Cochrane Database Syst Rev* 2006(3):Cd005657. doi: 10.1002/14651858.CD005657.pub2 [published Online First: 2006/07/21]
16. Hay AD, Downing H, Francis NA, et al. Anaesthetic-analgesic ear drops to reduce antibiotic consumption in children with acute otitis media: the CEDAR RCT. *Health Technol Assess* 2019;23(34):1-48. doi: 10.3310/hta23340 [published Online First: 2019/07/16]
17. Horne R. Treatment perceptions and self-regulation. In: Leventhal H, Cameron L, eds. The self-regulation of health and illness behaviour. London: Routledge 2003:138–53.
18. Yardley L, Douglas E, Anthierens S, et al. Evaluation of a web-based intervention to reduce antibiotic prescribing for LRTI in six European countries: quantitative process analysis of the GRACE/INTRO randomised controlled trial. *Implement Sci* 2013;8:134. doi: 10.1186/1748-5908-8-134 [published Online First: 2013/11/19]
19. van Uum RT, Sjoukes A, Venekamp RP, et al. Pain management in acute otitis media: a qualitative study exploring GPs' views and expectations parallel to a trial of an educational intervention. *BJGP Open*

For peer review only

2018;2(4):bjgpopen18X101620. doi: 10.3399/bjgpopen18X101620 [published Online First: 2019/02/07]
20. van Uum RT, Venekamp RP, Schilder AGM, et al. Pain management in acute otitis media: a qualitative study of parents' views and expectations. *BMC Fam Pract* 2019;20(1):18. doi: 10.1186/s12875-019-0908-9 [published Online First: 2019/01/25]
21. Braun V, Clarke V. What can "thematic analysis" offer health and wellbeing researchers? *Int J Qual Stud Health Well-being* 2014;9:26152. doi: 10.3402/qhw.v9.26152 [published Online First: 2014/10/19]
22. Gale NK, Heath G, Cameron E, et al. Using the framework method for the analysis of qualitative data in multi-disciplinary health research. *BMC Med Res Methodol* 2013;13:117. doi: 10.1186/1471-2288-13-117 [published Online First: 2013/09/21]
23. Damoiseaux RAMJ, Venekamp RP, Eekhof J. NHG-standaard OMA bij kinderen (derde herziening). *Huisarts Wet* 2014;57:648.
24. Farmacotherapeutisch Kompas [Available from: https://www.farmacotherapeutischkompas.nl/.
25. Dutch Children's Formulary 'Kinderformularium' [Available from: https://www.kinderformularium.nl/.
26. Little P, Gould C, Williamson I, et al. Pragmatic randomised controlled trial of two prescribing strategies for childhood acute otitis media. *Bmj* 2001;322(7282):336-42. doi: 10.1136/bmj.322.7282.336 [published Online First: 2001/02/13]
27. Raat H, Landgraf JM, Oostenbrink R, et al. Reliability and validity of the Infant and Toddler Quality of Life Questionnaire (ITQOL) in a general population and respiratory disease sample. *Qual Life Res* 2007;16(3):445-60. doi: 10.1007/s11136-006-9134-8 [published Online First: 2006/11/18]
28. Rosenfeld RM, Goldsmith AJ, Tetlus L, et al. Quality of life for children with otitis media. *Arch Otolaryngol Head Neck Surg* 1997;123(10):1049-54. doi: 10.1001/archotol.1997.01900100019002 [published Online First: 1997/10/27]
29. Bouwmans C, Krol M, Brouwer W, et al. IMTA Productivity Cost Questionnaire (IPCQ). *Value Health* 2014;17(7):A550. doi: 10.1016/j.jval.2014.08.1791 [published Online First: 2014/11/01]
30. Schulz KF, Altman DG, Moher D. CONSORT 2010 statement: updated guidelines for reporting parallel group randomised trials. *Bmj* 2010;340:c332. doi: 10.1136/bmj.c332 [published Online First: 2010/03/25]
31. Donders AR, van der Heijden GJ, Stijnen T, et al. Review: a gentle introduction to imputation of missing values. *J Clin Epidemiol* 2006;59(10):1087-91. doi: 10.1016/j.jclinepi.2006.01.014 [published Online First: 2006/09/19]
32. Rubin D. Multiple imputation for non-response in surveys. New York: John Wiley 1987.
33. Wolleswinkel-van den Bosch JH, Stolk EA, Francois M, et al. The health care burden and societal impact of acute otitis media in seven European countries: results of an Internet survey. *Vaccine* 2010;28 Suppl 6:G39-52. doi: 10.1016/j.vaccine.2010.06.014 [published Online First: 2010/11/16]
34. Zorginstituut Nederland. Kostenhandleiding: Methodologie van kostenonderzoek en referentieprijzen voor economische evaluaties in de gezondheidszorg. 2016 ed. Diemen: Zorginstituut Nederland 2016.
35. Zorginstituut Nederland. Richtlijn voor het uitvoeren van economische evaluaties in de gezondheidszorg. Diemen: Zorginstituut Nederland 2016.
36. Buskens E, van Staaij B, van den Akker J, et al. Adenotonsillectomy or watchful waiting in patients with mild to moderate symptoms of throat infections or adenotonsillar hypertrophy: a randomized comparison of costs and effects. *Arch Otolaryngol Head Neck Surg* 2007;133(11):1083-8. doi: 10.1001/archotol.133.11.1083 [published Online First: 2007/11/21]
37. Koopmanschap MA, Rutten FF, van Ineveld BM, et al. The friction cost method for measuring indirect costs of disease. *J Health Econ* 1995;14(2):171-89. doi: 10.1016/0167-6296(94)00044-5 [published Online First: 1995/05/08]
38. National Institute for Family Finance Information website [Available from: https://www.nibud.nl/consumenten/kosten-van-kinderopvang/.
39. Treweek S, Zwarenstein M. Making trials matter: pragmatic and explanatory trials and the problem of applicability. *Trials* 2009;10:37. doi: 10.1186/1745-6215-10-37 [published Online First: 2009/06/06]
40. Moustgaard H, Clayton GL, Jones HE, et al. Impact of blinding on estimated treatment effects in randomised clinical trials: meta-epidemiological study. *Bmj* 2020;368:l6802. doi: 10.1136/bmj.l6802 [published Online First: 2020/01/23]

For peer review only - http://bmjopen.bmj.com/site/about/guidelines.xhtml

Figure 1. Flow diagram of study procedures

Legend: *AOM: acute otitis media, GP: general practitioner, OPTIMA: trial acronym*
* Parents who gave consent for the semi-structured interview will be recruited from both the intervention and control arm and will be purposively sampled after follow-up.

Supplemental material 1. Methods and analyses mixed-method process evaluation

Supplemental material 2. Informed consent form

For peer review only

For peer review only - http://bmjopen.bmj.com/site/about/guidelines.xhtml

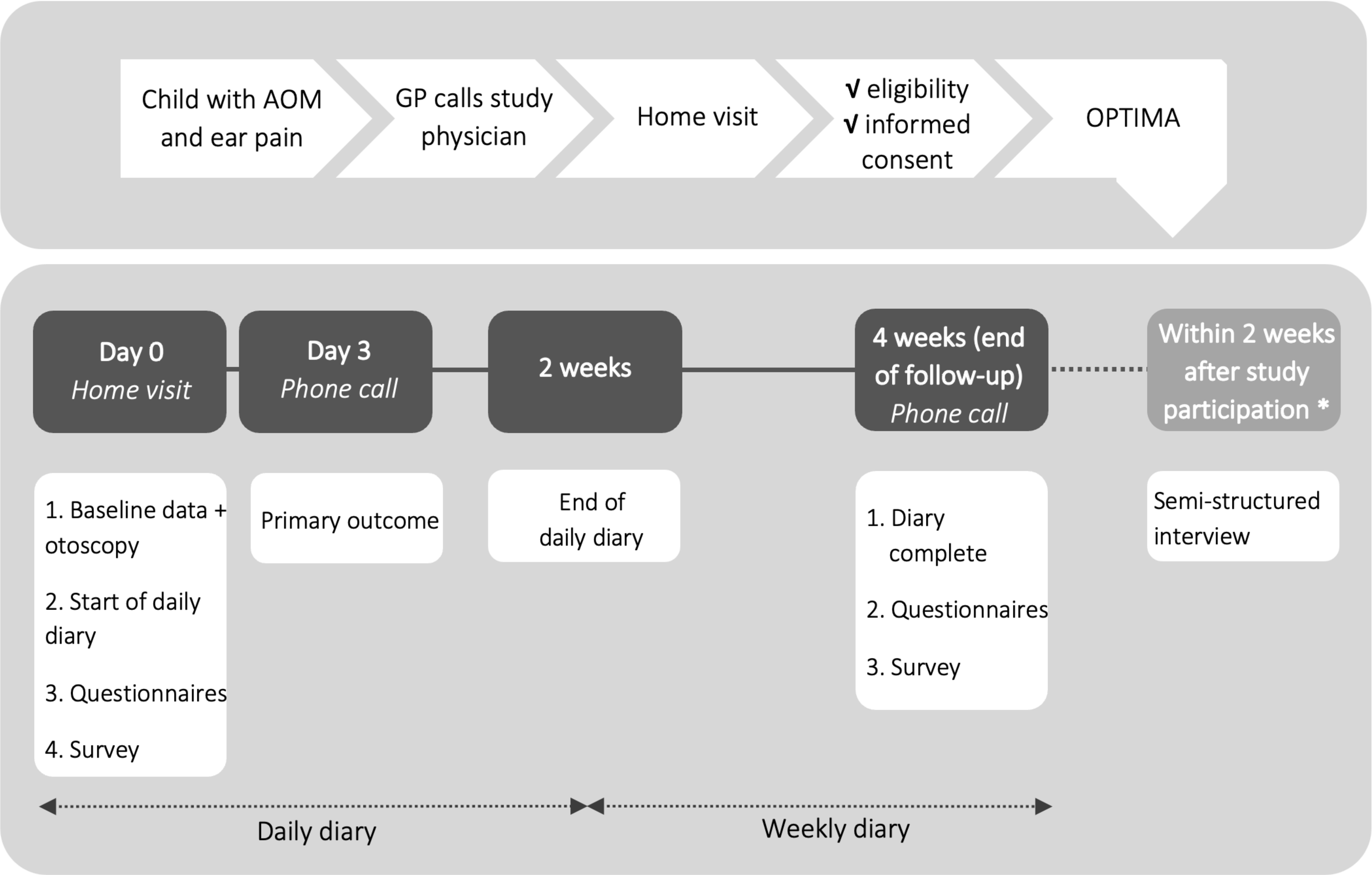
Child with AOM and ear pain
GP calls study physician
Home visit
√ eligibility
√ informed consent
OPTIMA
Day 0
Home visit
Day 3
Phone call
2 weeks
4 weeks (end of follow-up)
Phone call
Within 2 weeks after study participation *
1. Baseline data + otoscopy
2. Start of daily diary
3. Questionnaires
4. Survey
Primary outcome
End of daily diary
1. Diary complete
2. Questionnaires
3. Survey
Semi-structured interview
Daily diary
Weekly diary

Supplemental material 1. Methods and analyses mixed-method process evaluation

Surveys

Surveys will include brief statements which will be rated by parents on a 5-point scale (strongly disagree/ strongly agree). Parents of all trial participants will complete this survey at baseline and at 4 weeks (end of follow-up). Beliefs about the necessity and harms of antibiotics for AOM, oral analgesics and analgesic eardrops were assessed using questions based on the two key dimensions of treatment perceptions in the extended Common Sense Model: 'It is usually necessary to take antibiotics/oral analgesics/analgesic eardrops for an ear infection' and 'Taking antibiotics/oral analgesics/analgesic eardrops for an ear infection can do more harm than good'.[17] Patient satisfaction with the consultation will be assessed by a three-item Consultation Satisfaction Questionnaire that assessed whether they felt they had received all the information and advice they needed and were generally satisfied with the consultation.[18]

Semi-structured interviews

Semi-structured interviews will be carried out with parents and GPs participating in the trial. Interviews with parents will capture experiences of and satisfaction with care and subsequent views and experiences of management of symptoms (including eardrops and antibiotic use). Parents will be recruited from both the intervention and control arm and will be purposively sampled after study participation to obtain variation in age of child, symptom presentation, antibiotic prescribed, eardrops prescribed, gender, number of children, education level and employment status. Interviews will be carried out preferably within 2 weeks after study participation. Interviews with GPs will capture views and experiences of AOM consultations and the use of eardrops in these consultations. GPs will be purposively sampled to obtain variation in practice setting, gender, age and experience. Interviews with GPs will be carried out after the inclusion period. Data will be collected until saturation is reached; based on previous experience, we expect to conduct approximately 10 to 20 semi-structured interviews with both parents and GPs to reach saturation.[19 20]

Each interview will be audio recorded with the participant's permission. Recordings will allow verbatim transcription of interviews in Microsoft Word. Once transcripts are checked, recordings will be deleted. Transcripts will be labelled with a unique participant number and will omit any identifiable data either identifying the participant or their general practice.

Analysis of qualitative data

Qualitative data collection by means of semi-structured interviews and qualitative data analysis will be done concurrently. During the process, multiple members of the study team will review the transcripts of earlier interviews for accuracy and decide whether the topic lists need adaptation.

Interviews with parents and GPs, will be analysed using thematic and framework analysis taking an inductive approach.[21 22] NVivo software will be used to assist with the organisation of data. A thematic framework will be used to chart data across all interviews and will aid comparisons between participants.

For peer review only

Proefpersoneninformatie en toestemmingsformulier voor deelname aan medisch-wetenschappelijk onderzoek

OPTIMA: pijnstillende oordruppels bij acute middenoorontsteking

Officiële titel: Effectiviteit van pijnstillende oordruppels als toevoeging aan systemische pijnstillers bij kinderen met otitis media acuta: een pragmatische gerandomiseerde gecontroleerde studie

Inleiding

Geachte heer/mevrouw,

Met deze informatiebrief willen we u vragen of u met uw kind wilt meedoen aan medisch-wetenschappelijk onderzoek. Meedoen is vrijwillig. U krijgt deze brief omdat uw kind een acute middenoorontsteking heeft.
U leest hier om wat voor onderzoek het gaat, wat het voor u betekent, en wat de voordelen en nadelen zijn. Het is veel informatie. Wilt u de informatie doorlezen en beslissen of u wilt meedoen? Als u wilt meedoen, kunt u het formulier invullen dat u vindt in bijlage C.

Stel uw vragen

U kunt uw beslissing nemen met de informatie die u in deze informatiebrief vindt. Daarnaast raden we u aan om dit te doen:
- Stel vragen aan de onderzoeker die u deze informatie geeft.
- Praat met uw partner, familie of vrienden over dit onderzoek.
- Stel vragen aan de onafhankelijk deskundige, Dr. P.C.J.L. Bruijning-Verhagen (zie bijlage A voor contactgegevens).
- Lees de informatie op www.rijksoverheid.nl/mensenonderzoek.

1. Algemene informatie

Het Universitair Medisch Centrum (UMC) Utrecht heeft dit onderzoek opgezet. Voor dit onderzoek zijn 300 patiënten nodig. Dit onderzoek wordt gefinancierd door ZonMw. Deze organisatie financiert gezondheidsonderzoek in opdracht van het Ministerie van Volksgezondheid, Welzijn en Sport (VWS), en de Nederlandse Organisatie voor Wetenschappelijk Onderzoek (NWO).
De medisch-ethische toetsingscommissie Utrecht heeft dit onderzoek goedgekeurd.

For peer review only - http://bmjopen.bmj.com/site/about/guidelines.xhtml

Optima
pijnstillende oordruppels bij otitis media acuta

UMC Utrecht

2. Wat is het doel van het onderzoek?

In dit onderzoek bekijken we wat het effect is van pijnstillende oordruppels bij kinderen met een acute middenoorontsteking, een ontsteking van de holte achter het trommelvlies.

3. Wat is de achtergrond van het onderzoek?

Een acute middenoorontsteking komt vaak voor op de kinderleeftijd. Het wordt veroorzaakt door een virus of een bacterie. Standaardzorg bestaat momenteel voornamelijk uit pijnstillende behandeling (paracetamol en zo nodig ibuprofen) en indien nodig antibiotica. Ondanks de inzet van deze standaardzorg, gaat de aandoening gepaard met oorpijn en ongemak bij kinderen en kan het een aanzienlijke impact op het gehele gezin hebben door slapeloze nachten en school- en werkverzuim. Het is daarom belangrijk dat er gezocht blijft worden naar andere behandelingen die de klachten kunnen verlichten. Eerdere studies lieten zien dat pijnstillende oordruppels mogelijk een gunstig effect hebben, maar het huidige bewijs is van onvoldoende kwaliteit om een zekere uitspraak over het effect te kunnen doen. Daarom willen we in deze studie onderzoeken of de pijnstillende oordruppels een betere verlichting van de oorpijn geven dan de gebruikelijke pijnstillers alleen. We gaan dit onderzoeken door 300 kinderen middels loting toe te wijzen aan 1) pijnstillende oordruppels bovenop de standaardzorg of 2) standaardzorg

4. Hoe verloopt het onderzoek?

Hoelang duurt het onderzoek?

Doet uw kind mee met het onderzoek? Dan duurt dat in totaal 4 weken.

Stap 1: is uw kind geschikt om mee te doen?

We willen eerst weten of uw kind geschikt is om mee te doen. Uw huisarts controleert of uw kind in aanmerking komt voor studiedeelname. Vervolgens geeft uw huisarts – met uw goedkeuring – uw gegevens door aan het onderzoeksteam in het UMC Utrecht en geeft u deze informatiefolder mee. Het onderzoeksteam neemt telefonisch contact met u op en één van de onderzoekers bezoekt u thuis, binnen 24 uur na uw bezoek aan de huisarts.. Aanvullend aan de beoordeling van de huisarts, zal de onderzoeker u enkele vragen stellen als extra controle op geschiktheid voor deelname.
Tijdens dit bezoek zal de onderzoeker u verder informeren over de studie en mogelijke vragen die u heeft beantwoorden. Pas daarna besluit u of u met uw kind wilt deelnemen aan het onderzoek. Indien u besluit om mee te doen vragen we aan beide ouders/voogden om een toestemmingsformulier te ondertekenen. De onderzoeker ondertekent dit formulier ook. Indien één van de ouders/voogden tijdens het eerste huisbezoek niet aanwezig is, zal van de betreffende ouder mondeling toestemming worden gevraagd. Alleen indien deze ook (mondelinge) toestemming geeft voor deelname, kan uw kind deelnemen aan het onderzoek. In het betreffende geval zal een extra proefpersoneninformatie en toestemmingsformulier bij u thuis worden achtergelaten om door de andere ouder te laten ondertekenen. Het getekende formulier zal op een ander moment opgehaald worden. In bijlage D staat

For peer review only - http://bmjopen.bmj.com/site/about/guidelines.xhtml

bovenstaande procedure van informeren en toestemming geven (informed consent) op een schematische manier weergegeven.

Stap 2: de behandeling

Voor dit onderzoek verdelen we de deelnemende kinderen gelijk over 2 groepen:

- **Groep 1 (interventie groep)**. De kinderen in deze groep krijgen pijnstillende oordruppels (Otalgan® maximaal 6 keer/dag 1-2 druppels gedurende 7 dagen) bovenop de standaardzorg bestaande uit gebruikelijke pijnstillers en indien nodig naar inschatting van de huisarts antibiotica (50% van de kinderen).
  Let op: indien uw kind een loopoor krijgt dan moet u **direct stoppen** met het gebruik van de pijnstillende oordruppels in dat oor, neem in dat geval contact op met de onderzoeker.
- **Groep 2 (controle groep)**. De kinderen in deze groep krijgen de standaardzorg (50% van de kinderen).

Loting bepaalt in welke groep uw kind terecht zal komen.

Stap 3: verzamelen van gegevens

- Het **huisbezoek** op de dag van studiedeelname duurt circa één uur. Tijdens dit huisbezoek zal, nadat er toestemming gegeven is, een kort lichamelijk onderzoek worden verricht waarbij in de oren van uw kind gekeken wordt. Daarnaast neemt de onderzoeker de eerste vragenlijsten met u door. Aan het einde van het eerste huisbezoek zal de arts-onderzoeker via internet contact opnemen met het onderzoekscentrum in het UMC Utrecht om te zien wat het resultaat is van de loting die bepaalt welke behandeling uw kind krijgt. Dit geven wij door aan de huisarts en apotheek van uw kind. Indien uw kind in groep 1 ingedeeld wordt, ontvangt u aan het einde van het bezoek de oordruppels.
- We vragen u gedurende 4 weken om dagelijks een **klachtendagboekje** bij te houden. Dit kan online of op papier gedaan worden. U krijgt hier uitleg over tijdens het huisbezoek.
- Na **3 dagen** neemt een medewerker van het onderzoek **telefonisch contact** met u op om het dagboekje en eventuele vragen te bespreken. Dit duurt circa 10 minuten.
- Na **4 weken** neemt de onderzoeker nogmaals **telefonisch contact** met u op om de studie af te ronden en de laatste vragenlijsten met u door te nemen. Dit telefoon gesprek duurt maximaal 30 minuten.
- Optioneel kunt u ook nog deelnemen aan een telefonisch interview over uw ervaringen met de behandeling van de acute middenoorontsteking en de studie. Dit gebeurt alleen indien u hier toestemming voor geeft.

Wat is er anders dan bij gewone zorg?

Er is bij dit onderzoek niet zoveel anders dan bij gewone zorg. Indien uw kind in groep 1 geloot wordt, krijgt uw kind pijnstillende oordruppels bovenop de gewone zorg. Indien uw kind in groep 2 geplaatst wordt krijgt uw kind de gewone zorg.

For peer review only - http://bmjopen.bmj.com/site/about/guidelines.xhtml

5. Welke afspraken maken we met u?

We willen graag dat het onderzoek goed verloopt. Daarom maken we de volgende afspraken met u:

- U geeft het medicijn aan uw kind op de manier die de onderzoeker u heeft uitgelegd.
- Uw kind doet tijdens dit onderzoek niet ook nog mee aan een ander medisch-wetenschappelijk onderzoek.
- U vult de vragenlijsten en het dagboekje in zoals met u besproken is.
- U draagt de deelnemerskaart van het onderzoek bij u. Bijvoorbeeld in uw portemonnee. Hierop staat dat uw kind meedoet aan dit onderzoek. En wie men moet waarschuwen bij een noodsituatie. Laat deze kaart zien als uw kind bij een (andere) arts komt.
- Uw huisarts blijft ten alle tijden verantwoordelijk voor de medische behandeling van uw kind. Bij eventuele gezondheidsproblemen van uw kind neemt u contact op met uw huisarts zoals u normaal gesproken ook zou doen.
- U neemt contact op met de onderzoeker in deze specifieke situaties:
  - Uw kind krijgt een **loopoor**. In dit geval moet u **direct stoppen** met het gebruik van de pijnstillende oordruppels in dat oor.
  - Uw kind wordt in een ziekenhuis opgenomen of behandeld.
  - U en/of uw kind wilt niet meer meedoen met het onderzoek.
  - Uw telefoonnummer, adres of e-mailadres verandert.

6. Van welke bijwerkingen, nadelige effecten of ongemakken kunt u last krijgen?

Alle werkzame medicijnen kunnen bijwerkingen hebben. In de bijsluiter van de oordruppels staat dat er een kleine kans is op een overgevoeligheidsreactie. Deze bijwerking komt zelden voor (bij hooguit 1 op de 1.000 personen). We adviseren u dan om direct contact op te nemen met een (huis)arts.
In de bijsluiter van de oordruppels staat verder vermeld dat deze medicatie niet gebruikt dient te worden bij een loopoor of perforatie (gaatje/scheurtje) van het trommelvlies. Uw kind kan niet deelnemen aan het onderzoek als dit het geval is. Zowel de huisarts als de onderzoeker controleert dit aan het begin van het onderzoek door in de oren van uw kind te kijken.

Indien uw kind tijdens het onderzoek een **loopoor** krijgt mag uw kind de oordruppels **niet meer gebruiken**. Neem in dat geval contact op met de onderzoeker.

Doet u mee aan het onderzoek? Dan krijgt u de bijsluiter mee bij het middel.

7. Wat zijn de voordelen en de nadelen als u meedoet aan het onderzoek?

Meedoen aan het onderzoek kan voordelen en nadelen hebben. Hieronder zetten we ze op een rij. Denk hier goed over na, en praat erover met anderen.

For peer review only - http://bmjopen.bmj.com/site/about/guidelines.xhtml

Meedoen aan het onderzoek kan deze voordelen hebben:
De oordruppels kunnen mogelijk de mate van oorpijn van uw kind en het gebruik van antibiotica verminderen, maar zeker is dit niet.

Meedoen aan het onderzoek kan deze nadelen hebben:

- Uw kind kan last krijgen van bijwerkingen of nadelige effecten van de oordruppels, zoals beschreven in paragraaf 6.
- Meedoen aan het onderzoek kost u extra tijd.
- U moet zich houden aan de afspraken die horen bij het onderzoek.

Wilt u niet meedoen?
U beslist zelf of u met uw kind meedoet aan dit onderzoek. Deelname is geheel vrijwillig. Als u besluit niet mee te doen, hoeft u verder niets te doen. U hoeft niets te tekenen. U hoeft ook niet te zeggen waarom u niet wilt meedoen als u dat niet wilt. Uw kind krijgt dan gewoon de begeleiding en behandeling die hij/zij anders ook zou krijgen van uw huisarts.
Indien u wel meedoet, kunt u, of kan uw kind, zich op ieder moment van de studie bedenken en toch stoppen zonder de reden te melden. Dit zal geen gevolgen hebben voor de behandeling van uw kind.

8. Verzet van uw kind

Het kan gebeuren dat uw kind zich op een bepaald moment tijdens het onderzoek verzet (niet meewerkt). Indien u merkt dat dit het geval is, neem dan direct contact op met de onderzoeker. De onderzoeker moet het onderzoek dan direct stoppen. Het is moeilijk om precies te omschrijven wat verzet is. Voor de start van het onderzoek overleggen we met u wat wij zien als verzet.
De onderzoeker zal zich houden aan de Gedragscode 'Verzet van minderjarigen die deelnemen aan medisch-wetenschappelijk onderzoek' die door het NVK is vastgesteld.

9. Wanneer stopt het onderzoek?

De onderzoeker laat het u weten als er nieuwe informatie over het onderzoek komt die belangrijk voor u is. De onderzoeker vraagt u daarna of u blijft meedoen.

In deze situaties stopt voor u het onderzoek:

- Alle onderzoeken volgens het schema zijn voorbij.
- Het einde van het hele onderzoek is bereikt (na 4 weken)
- U of uw kind wilt zelf stoppen met het onderzoek. Dat mag op ieder moment. Meld dit dan meteen bij de onderzoeker. U hoeft er niet bij te vertellen waarom u stopt. Uw kind krijgt dan weer de gewone behandeling voor de middenoorontsteking. De onderzoeker zal u mogelijk nog wel uitnodigen voor een nagesprek.

For peer review only - http://bmjopen.bmj.com/site/about/guidelines.xhtml

- De onderzoeker vindt het beter voor u om te stoppen. De onderzoeker zal u mogelijk nog wel uitnodigen voor een nagesprek.
- Een van de volgende instanties besluit dat het onderzoek moet stoppen:
  - UMC Utrecht
  - de overheid, of
  - de medisch-ethische commissie die het onderzoek beoordeelt.

Wat gebeurt er als u stopt met het onderzoek?
De onderzoekers gebruiken de gegevens die tot het moment van stoppen zijn verzameld.

Het hele onderzoek is afgelopen als alle deelnemers klaar zijn.

10. Wat gebeurt er na het onderzoek?

Krijgt u de resultaten van het onderzoek?
Na afloop van de hele studie, als de gegevens van alle 300 kinderen zijn verzameld en verwerkt, ontvangt u van ons bericht over de belangrijkste uitkomsten van het onderzoek.

11. Wat doen we met de gegevens van uw kind?

Doet u mee met het onderzoek? Dan geeft u ook toestemming om de gegevens van uw kind te verzamelen, gebruiken en bewaren.

Welke gegevens bewaren we?
We bewaren deze gegevens:
- naam, geslacht en geboortedatum van uw kind
- adres, telefoonnummer en emailadres
- gegevens over gezondheid van uw kind
- (medische) gegevens die we tijdens het onderzoek verzamelen

Waarom verzamelen, gebruiken en bewaren we de gegevens van uw kind?
We verzamelen, gebruiken en bewaren de gegevens van uw kind om de vragen van dit onderzoek te kunnen beantwoorden. En om de resultaten te kunnen publiceren.

Hoe beschermen we de privacy van u en uw kind?
Om de privacy te beschermen geven wij de gegevens een code. Op de gegevens zetten we alleen deze code. De sleutel van de code bewaren we op een beveiligde plek in het UMC Utrecht. Als we de gegevens van uw kind verwerken, gebruiken we steeds alleen die code. Ook in rapporten en publicaties over het onderzoek kan niemand terughalen dat het over uw kind ging.

Wie kunnen de gegevens van uw kind zien?

For peer review only - http://bmjopen.bmj.com/site/about/guidelines.xhtml

Optima
pijnstillende oordruppels bij otitis media acuta

UMC Utrecht

Sommige personen kunnen wel de naam en andere persoonlijke gegevens van uw kind zonder code inzien. Dit zijn mensen die controleren of de onderzoekers het onderzoek goed en betrouwbaar uitvoeren. Deze personen kunnen bij de gegevens van uw kind komen:

- Een controleur die voor het UMC Utrecht werkt.
- Nationale toezichthoudende autoriteiten. Bijvoorbeeld de Inspectie Gezondheidszorg en Jeugd.

Deze personen houden de gegevens van uw kind geheim. Wij vragen u voor deze inzage toestemming te geven.

Hoelang bewaren we de gegevens van uw kind?
We bewaren de gegevens van uw kind 25 jaar.

Mogen we de gegevens van uw kind gebruiken voor ander onderzoek?
De gegevens van uw kind kunnen na afloop van dit onderzoek ook nog van belang zijn voor ander wetenschappelijk onderzoek op het gebied van acute middenoorontsteking. Daarvoor zullen de gegevens van uw kind 25 jaar worden bewaard in het onderzoekscentrum. In het toestemmingformulier geeft u aan of u dit goed vindt. Geeft u geen toestemming? Dan kunt u nog steeds meedoen met dit onderzoek. Uw kind krijgt dezelfde zorg.

Kunt u uw toestemming voor het gebruik van de gegevens van uw kind weer intrekken?
U kunt uw toestemming voor het gebruik van de gegevens van uw kind op ieder moment intrekken. Dit geldt voor het gebruik in dit onderzoek en voor het gebruik in ander onderzoek. Maar let op: trekt u uw toestemming in, en hebben onderzoekers dan al gegevens verzameld voor een onderzoek? Dan mogen zij deze gegevens nog wel gebruiken.

Mogelijke sturen we de gegevens van uw kind gecodeerd naar landen buiten de Europese Unie
In dit onderzoek kan het zijn dat we de gecodeerde gegevens van uw kind ook naar landen buiten de Europese Unie sturen. In die landen gelden niet de privacyregels van de Europese Unie. Maar de privacy van uw kind zal op een gelijkwaardig niveau worden beschermd.

Wilt u meer weten over de privacy van uw kind?

- Wilt u meer weten over de rechten van uw kind bij de verwerking van persoonsgegevens? Kijk dan op www.autoriteitpersoonsgegevens.nl.
- Heeft u vragen over de rechten van uw kind? Of heeft u een klacht over de verwerking van de persoonsgegevens van uw kind? Neem dan contact op met degene die verantwoordelijk is voor de verwerking van de persoonsgegevens van uw kind. Voor uw onderzoek is dat:
    - Het UMC Utrecht. Zie bijlage A voor contactgegevens, en website.
- Als u klachten heeft over de verwerking van de persoonsgegevens van uw kind, raden we u aan om deze eerst te bespreken met het onderzoeksteam. U kunt ook naar de

For peer review only - http://bmjopen.bmj.com/site/about/guidelines.xhtml
For peer review only

Functionaris Gegevensbescherming van het UMC Utrecht gaan. Of u dient een klacht in bij de Autoriteit Persoonsgegevens.

Waar vindt u meer informatie over het onderzoek?
Op de volgende website vindt u meer informatie over het onderzoek: https://www.trialregister.nl/trial/9500. Na het onderzoek kan de website een samenvatting van de resultaten van dit onderzoek tonen. U vindt het onderzoek door te zoeken op ‘NL9500’.

12. Krijgt u een vergoeding als uw kind meedoet aan het onderzoek?

Aan het onderzoek zijn voor u geen kosten verbonden. Er is geen vergoeding beschikbaar voor deelname, na afloop van het huisbezoek krijgt uw kind wel een klein cadeautje.

13. Bent u verzekerd tijdens het onderzoek?

Voor iedereen die meedoet aan dit onderzoek is een verzekering afgesloten. De verzekering betaalt voor schade door het onderzoek. Maar niet voor alle schade. In **bijlage B** vindt u meer informatie over de verzekering en de uitzonderingen. Daar staat ook aan wie u schade kunt melden.

14. We informeren de huisarts en apotheker van uw kind

De onderzoeker stuurt de huisarts en apotheker van uw kind een bericht om te laten weten dat u meedoet aan het onderzoek. Daarnaast vragen we de apotheek naar bekende allergieën. Dit is voor de eigen veiligheid van uw kind.

15. Heeft u vragen?

Vragen over het onderzoek kunt u stellen aan het onderzoeksteam. Wilt u advies van iemand die er geen belang bij heeft? Dan kunt u terecht bij een onafhankelijke deskundige. Voor ons onderzoek is dat dr. P.C.J.L. Bruijning-Verhagen (zie bijlage A voor contactgegevens). Zij weet veel over het onderzoek, maar werkt niet mee aan dit onderzoek.
Heeft u een klacht? Bespreek dit dan met de onderzoeker of de arts die u behandelt. Wilt u dit liever niet? Ga dan naar Klachtenbemiddeling van het UMC Utrecht. In bijlage A staat waar u die kunt vinden.

16. Hoe geeft u toestemming voor het onderzoek?

U kunt eerst rustig nadenken over dit onderzoek. Daarna vertelt u de onderzoeker of u de informatie begrijpt en of u wel of niet wilt meedoen met uw kind. Wilt u met uw kind meedoen? Dan vult u het toestemmingsformulier in dat u bij deze informatiebrief vindt. U en de onderzoeker krijgen allebei een getekende versie van deze toestemmingsverklaring.

Dank voor uw tijd.

For peer review only - http://bmjopen.bmj.com/site/about/guidelines.xhtml

Met vriendelijke groet,

Drs. Joline de Sévaux, arts-onderzoeker, mede namens OPTIMA onderzoeksteam:

Dr. Roderick P Venekamp, huisarts

Professor dr. Roger AMJ Damoiseaux, huisarts

Professor dr. Anne GM Schilder, KNO-arts

Drs. Saskia Hullegie, arts-onderzoeker

17. Bijlagen bij deze informatie

A. Contactgegevens
B. Informatie over de verzekering
C. Toestemmingsformulier
D. Procedure informeren en toestemming geven (informed consent)

For peer review only

For peer review only - http://bmjopen.bmj.com/site/about/guidelines.xhtml

Bijlage A: contactgegevens voor UMC Utrecht

Contactpersoon onderzoeksteam:
Naam: J.L.H. de Sévaux, arts-onderzoeker
Julius Centrum voor Gezondheidswetenschappen en Eerstelijnsgeneeskunde, Afdeling Huisartsgeneeskunde
Universitair Medisch Centrum Utrecht
e-mail: optima@umcutrecht.nl
tel. nummer: 0625710099
Bereikbaar: dagelijks tijdens kantooruren.

Onafhankelijk deskundige:
Naam: Dr. P.C.J.L. Bruijning-Verhagen, kinderarts
UMC Utrecht
email: p.bruijning@umcutrecht.nl
tel. nummer: 088 75 681 81

Klachten:
Als u klachten heeft kunt u dit melden aan de onderzoeker of aan uw behandelend arts. Mocht u ontevreden zijn over de gang van zaken bij het onderzoek en een klacht willen indienen dan kunt u contact opnemen met de klachtenbemiddelaars. Deze zijn bereikbaar via tel. +31 88 755 62 08. Of digitaal via: https://www.umcutrecht.nl/nl/een-klacht-indienen

Functionaris voor de Gegevensbescherming van de instelling: privacy@umcutrecht.nl

Raadpleeg de website van het UMC Utrecht voor meer informatie over uw rechten:
https://www.umcutrecht.nl/nl/ziekenhuis/privacy

For peer review only

For peer review only - http://bmjopen.bmj.com/site/about/guidelines.xhtml

Bijlage B: informatie over de verzekering

UMC Utrecht heeft een verzekering afgesloten voor iedereen die meedoet aan het onderzoek. De verzekering betaalt de schade die u heeft doordat u aan het onderzoek meedeed. Het gaat om schade die u krijgt tijdens het onderzoek, of binnen 4 jaar na het onderzoek. U moet schade binnen 4 jaar melden bij de verzekeraar.

Heeft u schade door het onderzoek? Meld dit dan telefonisch of per post bij deze verzekeraar:

De verzekeraar van het onderzoek is:	
Naam:	CNA Insurance Company Ltd
Adres:	Polarisavenue 140, 2132 JX Hoofddorp
Telefoonnummer:	020 57 37 274
Polisnummer:	10201366
Contactpersoon:	Mw. Esther van Herk

De verzekering betaalt maximaal € 650.000 per persoon en € 5.000.000 voor het hele onderzoek en € 7.500.000 per jaar voor alle onderzoeken van dezelfde opdrachtgever (het UMC Utrecht).

Let op: de verzekering dekt de volgende schade **niet**:

- Schade door een risico waarover we u informatie hebben gegeven in deze brief. Maar dit geldt niet als het risico groter bleek te zijn dan we van tevoren dachten. Of als het risico heel onwaarschijnlijk was.
- Schade aan uw gezondheid die ook zou zijn ontstaan als u niet aan het onderzoek had meegedaan.
- Schade die ontstaat doordat u aanwijzingen of instructies niet of niet goed opvolgde.
- Schade aan de gezondheid van uw kinderen of kleinkinderen.
- Schade door een behandelmethode die al bestaat. Of door onderzoek naar een behandelmethode die al bestaat.

Deze bepalingen staan in het 'Besluit verplichte verzekering bij medisch-wetenschappelijk onderzoek met mensen 2015'. Dit besluit staat in de Wettenbank van de overheid (https://wetten.overheid.nl).

For peer review only

For peer review only - http://bmjopen.bmj.com/site/about/guidelines.xhtml

Bijlage C: Toestemmingsformulier ouders of voogd

Behorende bij de **OPTIMA-studie (pijnstillende oordruppels bij acute middenoorontsteking)**

Ik ben gevraagd om toestemming te geven voor deelname van mijn kind aan dit medisch-wetenschappelijke onderzoek:

Naam proefpersoon (kind): …………………….. Geboortedatum: ___ / ___ / _____

- Ik heb de informatiebrief voor de ouders/verzorgers gelezen. Ook kon ik vragen stellen. Mijn vragen zijn goed genoeg beantwoord. Ik had genoeg tijd om te beslissen of ik wil dat mijn kind meedoet.
- Ik weet dat meedoen vrijwillig is. Ook weet ik dat ik op ieder moment kan beslissen dat mijn kind toch niet meedoet. Ik hoef dan niet te zeggen waarom ik dat wil.
- Ik geef de onderzoeker toestemming om mijn huisarts en apotheker te laten weten dat mijn kind meedoet aan dit onderzoek.
- Ik geef de onderzoekers toestemming om de gegevens van mijn kind te verzamelen en te gebruiken. De onderzoekers doen dit alleen om de onderzoeksvraag in dit onderzoek te beantwoorden.
- Ik weet dat voor de controle van het onderzoek sommige mensen toegang tot alle gegevens van mijn kind kunnen krijgen. Die mensen staan in deze informatiebrief. Ik geef deze mensen toestemming om de gegevens van mijn kind in te zien voor deze controle.
- Ik weet dat mijn gecodeerde gegevens naar landen buiten de EU kunnen worden gestuurd waar privacyregels van de EU niet gelden.
- Wilt u in de tabel hieronder ja of nee aankruisen?

Ik geef toestemming om mij na dit onderzoek te benaderen voor een interview over uw ervaringen met betrekking tot de studiedeelname en de behandeling.	Ja ☐	Nee☐
Ik geef toestemming om mijn kind na dit onderzoek te vragen of hij/zij wil meedoen met een vervolgonderzoek.	Ja ☐	Nee☐
Ik geef toestemming om de gegevens van mijn kind te bewaren om dit te gebruiken voor ander onderzoek, zoals in de informatiebrief staat.	Ja ☐	Nee☐

- Ik ga ermee akkoord dat mijn kind meedoet aan dit onderzoek.

For peer review only

For peer review only - http://bmjopen.bmj.com/site/about/guidelines.xhtml

Optima
pijnstillende oordruppels bij otitis media acuta

UMC Utrecht

Naam ouder/voogd**: ………………………………

Handtekening: ………………………………… Datum: ___ / ___ / _____

Naam andere ouder/voogd**: ……………………

Handtekening: ……………………………………… Datum: ___ / ___ / _____

Ik verklaar dat ik de persoon/personen hierboven volledig heb geïnformeerd over het genoemde onderzoek.

Wordt er tijdens het onderzoek informatie bekend die de toestemming van de ouder of voogd kan beïnvloeden? Dan laat ik dit op tijd aan hem/haar weten.

Naam onderzoeker (of diens vertegenwoordiger): …………………………

Handtekening: ……………………… Datum: ___ / ___ / _____

---

* Doorhalen wat niet van toepassing is.
** Als het kind jonger dan 16 jaar is, ondertekenen de ouders die het gezag uitoefenen of de voogd dit formulier. Kinderen van 12 t/m 15 jaar die zelfstandig beslissingen kunnen nemen (wilsbekwaam zijn), moeten daarnaast zelf een formulier ondertekenen

De ouder/voogd krijgt een volledige informatiebrief mee, samen met een getekende versie van het toestemmingsformulier.

For peer review only

For peer review only - http://bmjopen.bmj.com/site/about/guidelines.xhtml

Bijlage D: Procedure informeren en toestemming geven (informed consent)

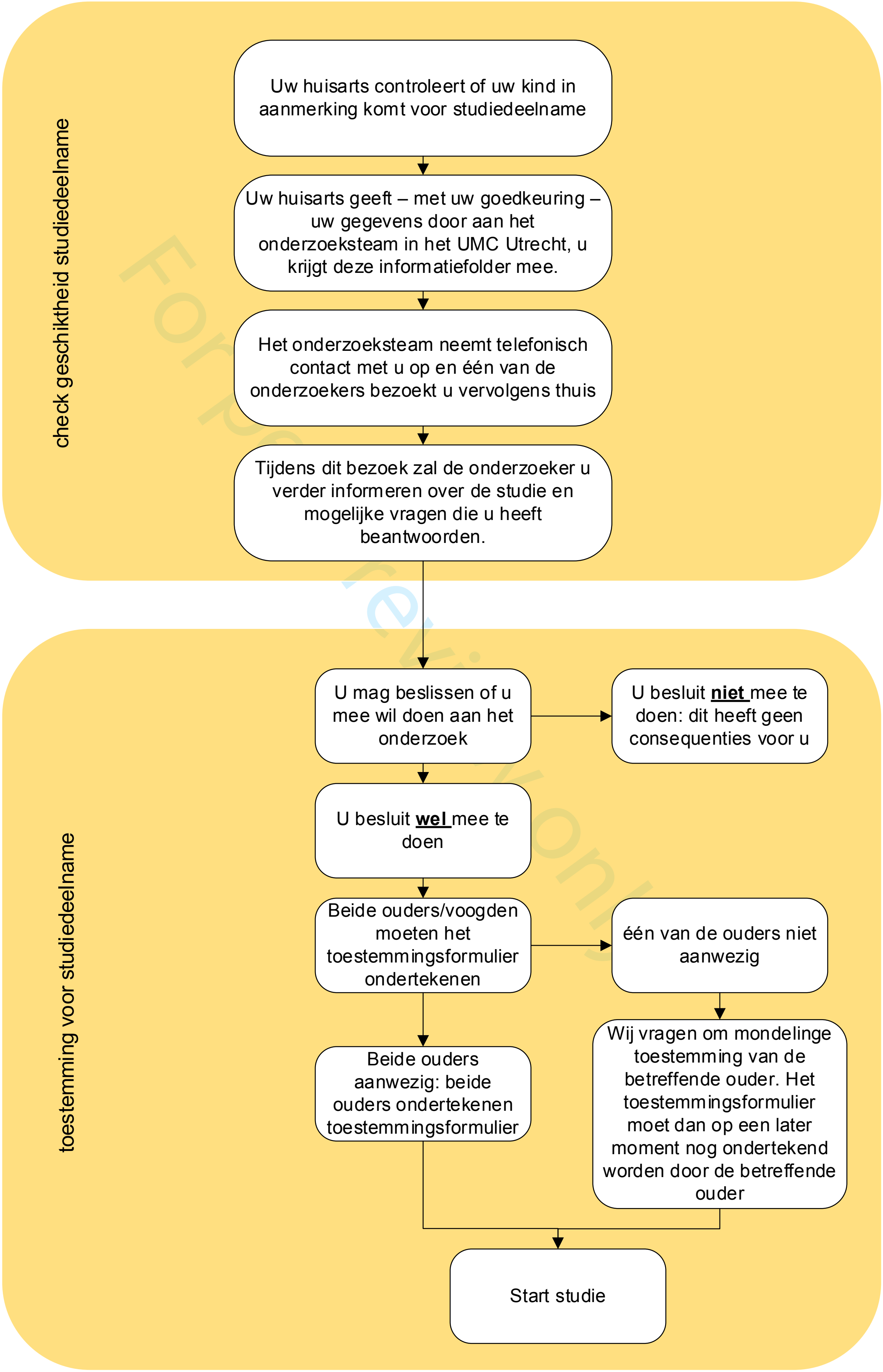

For peer review only - http://bmjopen.bmj.com/site/about/guidelines.xhtml

SPIRIT

STANDARD PROTOCOL ITEMS: RECOMMENDATIONS FOR INTERVENTIONAL TRIALS

SPIRIT 2013 Checklist: Recommended items to address in a clinical trial protocol and related documents*

Section/item	Item No	Description	Page number
**Administrative information**			
Title	1	Descriptive title identifying the study design, population, interventions, and, if applicable, trial acronym	1
Trial registration	2a	Trial identifier and registry name. If not yet registered, name of intended registry	2
	2b	All items from the World Health Organization Trial Registration Data Set	2
Protocol version	3	Date and version identifier	n/a
Funding	4	Sources and types of financial, material, and other support	13
Roles and responsibilities	5a	Names, affiliations, and roles of protocol contributors	1
	5b	Name and contact information for the trial sponsor	n/a
	5c	Role of study sponsor and funders, if any, in study design; collection, management, analysis, and interpretation of data; writing of the report; and the decision to submit the report for publication, including whether they will have ultimate authority over any of these activities	13
	5d	Composition, roles, and responsibilities of the coordinating centre, steering committee, endpoint adjudication committee, data management team, and other individuals or groups overseeing the trial, if applicable (see Item 21a for data monitoring committee)	11
**Introduction**			
Background and rationale	6a	Description of research question and justification for undertaking the trial, including summary of relevant studies (published and unpublished) examining benefits and harms for each intervention	4
	6b	Explanation for choice of comparators	4
Objectives	7	Specific objectives or hypotheses	4

For peer review only - http://bmjopen.bmj.com/site/about/guidelines.xhtml

Trial design	8	Description of trial design including type of trial (eg, parallel group, crossover, factorial, single group), allocation ratio, and framework (eg, superiority, equivalence, noninferiority, exploratory)	5

Methods: Participants, interventions, and outcomes

Study setting	9	Description of study settings (eg, community clinic, academic hospital) and list of countries where data will be collected. Reference to where list of study sites can be obtained	5
Eligibility criteria	10	Inclusion and exclusion criteria for participants. If applicable, eligibility criteria for study centres and individuals who will perform the interventions (eg, surgeons, psychotherapists)	5,16 (Box 1)
Interventions	11a	Interventions for each group with sufficient detail to allow replication, including how and when they will be administered	6
	11b	Criteria for discontinuing or modifying allocated interventions for a given trial participant (eg, drug dose change in response to harms, participant request, or improving/worsening disease)	6
	11c	Strategies to improve adherence to intervention protocols, and any procedures for monitoring adherence (eg, drug tablet return, laboratory tests)	6
	11d	Relevant concomitant care and interventions that are permitted or prohibited during the trial	n/a
Outcomes	12	Primary, secondary, and other outcomes, including the specific measurement variable (eg, systolic blood pressure), analysis metric (eg, change from baseline, final value, time to event), method of aggregation (eg, median, proportion), and time point for each outcome. Explanation of the clinical relevance of chosen efficacy and harm outcomes is strongly recommended	7,8
Participant timeline	13	Time schedule of enrolment, interventions (including any run-ins and washouts), assessments, and visits for participants. A schematic diagram is highly recommended (see Figure)	5,6, Figure 1
Sample size	14	Estimated number of participants needed to achieve study objectives and how it was determined, including clinical and statistical assumptions supporting any sample size calculations	8
Recruitment	15	Strategies for achieving adequate participant enrolment to reach target sample size	5

Methods: Assignment of interventions (for controlled trials)

Allocation:

For peer review only - http://bmjopen.bmj.com/site/about/guidelines.xhtml

Sequence generation	16a	Method of generating the allocation sequence (eg, computer-generated random numbers), and list of any factors for stratification. To reduce predictability of a random sequence, details of any planned restriction (eg, blocking) should be provided in a separate document that is unavailable to those who enrol participants or assign interventions	6
Allocation concealment mechanism	16b	Mechanism of implementing the allocation sequence (eg, central telephone; sequentially numbered, opaque, sealed envelopes), describing any steps to conceal the sequence until interventions are assigned	6
Implementation	16c	Who will generate the allocation sequence, who will enrol participants, and who will assign participants to interventions	6
Blinding (masking)	17a	Who will be blinded after assignment to interventions (eg, trial participants, care providers, outcome assessors, data analysts), and how	n/a (open trial)
	17b	If blinded, circumstances under which unblinding is permissible, and procedure for revealing a participant's allocated intervention during the trial	n/a
**Methods: Data collection, management, and analysis**			
Data collection methods	18a	Plans for assessment and collection of outcome, baseline, and other trial data, including any related processes to promote data quality (eg, duplicate measurements, training of assessors) and a description of study instruments (eg, questionnaires, laboratory tests) along with their reliability and validity, if known. Reference to where data collection forms can be found, if not in the protocol	5-7
	18b	Plans to promote participant retention and complete follow-up, including list of any outcome data to be collected for participants who discontinue or deviate from intervention protocols	5,6
Data management	19	Plans for data entry, coding, security, and storage, including any related processes to promote data quality (eg, double data entry; range checks for data values). Reference to where details of data management procedures can be found, if not in the protocol	8
Statistical methods	20a	Statistical methods for analysing primary and secondary outcomes. Reference to where other details of the statistical analysis plan can be found, if not in the protocol	9,10
	20b	Methods for any additional analyses (eg, subgroup and adjusted analyses)	9,10

	20c	Definition of analysis population relating to protocol non-adherence (eg, as randomised analysis), and any statistical methods to handle missing data (eg, multiple imputation)	9,10
**Methods: Monitoring**			
Data monitoring	21a	Composition of data monitoring committee (DMC); summary of its role and reporting structure; statement of whether it is independent from the sponsor and competing interests; and reference to where further details about its charter can be found, if not in the protocol. Alternatively, an explanation of why a DMC is not needed	11
	21b	Description of any interim analyses and stopping guidelines, including who will have access to these interim results and make the final decision to terminate the trial	11
Harms	22	Plans for collecting, assessing, reporting, and managing solicited and spontaneously reported adverse events and other unintended effects of trial interventions or trial conduct	6
Auditing	23	Frequency and procedures for auditing trial conduct, if any, and whether the process will be independent from investigators and the sponsor	n/a
**Ethics and dissemination**			
Research ethics approval	24	Plans for seeking research ethics committee/institutional review board (REC/IRB) approval	11
Protocol amendments	25	Plans for communicating important protocol modifications (eg, changes to eligibility criteria, outcomes, analyses) to relevant parties (eg, investigators, REC/IRBs, trial participants, trial registries, journals, regulators)	n/a
Consent or assent	26a	Who will obtain informed consent or assent from potential trial participants or authorised surrogates, and how (see Item 32)	5, supplemental material 2
	26b	Additional consent provisions for collection and use of participant data and biological specimens in ancillary studies, if applicable	supplemental material 2
Confidentiality	27	How personal information about potential and enrolled participants will be collected, shared, and maintained in order to protect confidentiality before, during, and after the trial	8
Declaration of interests	28	Financial and other competing interests for principal investigators for the overall trial and each study site	13
Access to data	29	Statement of who will have access to the final trial dataset, and disclosure of contractual agreements that limit such access for investigators	8

review only

For peer review only - http://bmjopen.bmj.com/site/about/guidelines.xhtml

Ancillary and post-trial care	30	Provisions, if any, for ancillary and post-trial care, and for compensation to those who suffer harm from trial participation	n/a
Dissemination policy	31a	Plans for investigators and sponsor to communicate trial results to participants, healthcare professionals, the public, and other relevant groups (eg, via publication, reporting in results databases, or other data sharing arrangements), including any publication restrictions	11
	31b	Authorship eligibility guidelines and any intended use of professional writers	n/a
	31c	Plans, if any, for granting public access to the full protocol, participant-level dataset, and statistical code	n/a
**Appendices**			
Informed consent materials	32	Model consent form and other related documentation given to participants and authorised surrogates	Supplemental material 2
Biological specimens	33	Plans for collection, laboratory evaluation, and storage of biological specimens for genetic or molecular analysis in the current trial and for future use in ancillary studies, if applicable	n/a

*It is strongly recommended that this checklist be read in conjunction with the SPIRIT 2013 Explanation & Elaboration for important clarification on the items. Amendments to the protocol should be tracked and dated. The SPIRIT checklist is copyrighted by the SPIRIT Group under the Creative Commons "Attribution-NonCommercial-NoDerivs 3.0 Unported" license.

For peer review only

Effectiveness of analgesic ear drops as add-on treatment to oral analgesics in children with acute otitis media: study protocol of the OPTIMA pragmatic randomised controlled trial

Journal:	*BMJ Open*
Manuscript ID	bmjopen-2022-062071.R2
Article Type:	Protocol
Date Submitted by the Author:	03-Jan-2023

Complete List of Authors:	de Sévaux, Joline; University Medical Centre Utrecht, Julius Center for Health Sciences and Primary Care<br>Damoiseaux, Roger; University Medical Centre Utrecht, Julius Center for Health Sciences and Primary Care<br>Hullegie, Saskia; University Medical Centre Utrecht, Julius Center for Health Sciences and Primary Care<br>Sanders, Elisabeth ; Wilhelmina Children's Hospital University Medical Centre, Department of Paediatric Immunology and Infectious Diseases; National Institute for Public Health and the Environment, Centre for Infectious Disease Control<br>Wit, Ardine; University Medical Centre Utrecht, Julius Center for Health Sciences and Primary Care; National Institute for Public Health and the Environment, Centre for Nutrition, Prevention and Healthcare<br>Zuithoff, Nicolaas; University Medical Centre Utrecht, Julius Center for Health Sciences and Primary Care<br>Yardley, Lucy; University of Southampton, School of Psychology, Faculty of Environmental and Life Sciences; University of Bristol, School of Psychological Science, Faculty of Life Sciences<br>Anthierens , Sibyl ; University of Antwerp Faculty of Medicine and Health Sciences, Department of Family Medicine and Population Health<br>Little, Paul; University of Southampton, Aldermoor Health Centre, Primary Care Research Centre, Primary Care Population Sciences and Medical Education, Faculty of Medicine<br>Hay, Alastair; University of Bristol, Centre for Academic Primary Care, Bristol medical School: Population Health Sciences<br>Schilder, Anne; University Medical Centre Utrecht, Julius Center for Health Sciences and Primary Care; NIHR University College London Hospitals Biomedical Research Centre<br>Venekamp , Roderick ; University Medical Centre Utrecht, Julius Center for Health Sciences and Primary Care

**Primary Subject Heading**:	General practice / Family practice
Secondary Subject Heading:	Ear, nose and throat/otolaryngology, Epidemiology, Evidence based practice, Infectious diseases, Qualitative research
Keywords:	PRIMARY CARE, Epidemiology < INFECTIOUS DISEASES, Paediatric

	otolaryngology < OTOLARYNGOLOGY, Paediatric infectious disease & immunisation < PAEDIATRICS, PAIN MANAGEMENT, QUALITATIVE RESEARCH

SCHOLARONE™
Manuscripts

Effectiveness of analgesic ear drops as add-on treatment to oral analgesics in children with acute otitis media: study protocol of the OPTIMA pragmatic randomised controlled trial

Joline LH de Sévaux[1], Roger AMJ Damoiseaux[1], Saskia Hullegie[1], Elisabeth AM Sanders[2,3], G Ardine de Wit[1,4], Nicolaas P A Zuithoff[1], Lucy Yardley[5,6], Sibyl Anthierens[7], Paul Little[8], Alastair D Hay[9], Anne GM Schilder[1,10,11], Roderick P Venekamp[1]

[1] Julius Center for Health Sciences and Primary Care, University Medical Center Utrecht, Utrecht University, Utrecht, The Netherlands

[2] Department of Paediatric Immunology and Infectious Diseases, Wilhelmina Children's Hospital, University Medical Center Utrecht, Utrecht University, Utrecht, The Netherlands

[3] Centre for Infectious Disease Control, National Institute of Public Health and the Environment (RIVM), Bilthoven, The Netherlands

[4] Centre for Nutrition, Prevention and Healthcare, National Institute of Public Health and the Environment (RIVM), Bilthoven, the Netherlands

[5]School of Psychological Science, Faculty of Life Sciences, University of Bristol, Bristol, United Kingdom

[6] School of Psychology, Faculty of Environmental and Life Sciences, University of Southampton, Southampton, United Kingdom

[7] Department of Family Medicine and Population Health, Faculty of Medicine and Health Sciences, University of Antwerp, Antwerp, Belgium

[8] Primary Care Research Centre, Primary Care Population Sciences and Medical Education, Faculty of Medicine, University of Southampton, Aldermoor Health CentreSouthampton, United Kingdom

[9] Centre for Academic Primary Care, Bristol medical School: Population Health Sciences, University of Bristol, Bristol, United Kingdom

[10] National Institute for Health Research University College London Hospitals Biomedical Research Centre

[11] evidENT, Ear Institute, University College London, London, United Kingdom

For peer review only

For peer review only - http://bmjopen.bmj.com/site/about/guidelines.xhtml

Corresponding author

Joline LH de Sévaux

Julius Center for Health Sciences and Primary Care

University Medical Center Utrecht

Str. 6.131, P.O. Box 85500, 3508 GA Utrecht, the Netherlands

Tel: +31 6 25 71 00 99

Email: j.l.h.desevaux-2@umcutrecht.nl

For peer review only

For peer review only - http://bmjopen.bmj.com/site/about/guidelines.xhtml

ABSTRACT

Introduction

Ear pain is the most prominent symptom of childhood acute otitis media (AOM). To control the pain and reduce reliance on antibiotics, evidence of effectiveness for alternative interventions is urgently needed. This trial aims to investigate whether analgesic ear drops added to usual care provide superior ear pain relief over usual care alone in children presenting to primary care with AOM.

Methods and analysis

This is a pragmatic, two arm, individually randomised, open, superiority trial with cost-effectiveness analysis and nested mixed-methods process evaluation in general practices in the Netherlands. We aim to recruit 300 children aged 1-6 years with a general practitioner (GP)-diagnosis of AOM and ear pain. Children will be randomly allocated (ratio 1:1) to either 1) lidocaine hydrochloride 5mg/g ear drops (Otalgan) 1-2 drops up to six times daily for a maximum of 7 days in addition to usual care (oral analgesics, with/without antibiotics) or 2) usual care. Parents will complete a symptom diary for 4 weeks as well as generic and disease-specific quality of life questionnaires at baseline and 4 weeks. The primary outcome is the parent-reported ear pain score (0-10) over the first 3 days. Secondary outcomes include proportion of children consuming antibiotics, oral analgesic use and overall symptom burden in the first 7 days; number of days with ear pain, number of GP reconsultations and subsequent antibiotic prescribing, adverse events, complications of AOM and cost-effectiveness during 4-week follow-up; generic and disease-specific quality of life at 4 weeks; parents' and GPs' views and experiences with treatment acceptability, usability and satisfaction.

For peer review only - http://bmjopen.bmj.com/site/about/guidelines.xhtml

For peer review only

Ethics and dissemination

The Medical Research Ethics Committee Utrecht, the Netherlands, has approved the protocol (21-447/G-D). All parents/guardians of participants will provide written informed consent. Study results will be submitted for publication in peer reviewed medical journals and presented at relevant (inter)national scientific meetings.

Trial registration: The Netherlands Trial Register; NL9500. Date of registration: 28 May 2021. At the time of publication of the study protocol paper, we were unable to make any amendments to the trial registration record in the Netherlands Trial Register. The addition of a data sharing plan was required to adhere to the International Committee of Medical Journal Editors (ICMJE) guidelines. The trial was therefore re-registered in ClinicalTrials.gov (NCT05651633; date of registration: 15 December 2022). This second registration is for modification purposes only and the NTR record (NL9500) should be regarded as the primary trial registration.

Word count: 3586 (excluding title page, abstract, references, figures and boxes).

Keywords: acute otitis media; children, primary care; analgesic ear drops; randomised controlled trial.

For peer review only

ARTICLE SUMMARY

Strengths

- The pragmatic, open design enhances the applicability of the trial findings in daily practice.
- The pragmatic, open design is most suited to address the key secondary outcomes of the impact of the analgesic ear drops on the proportion of children consuming antibiotics and its cost-effectiveness in everyday practice.

Limitations

- The combination of the open design and the subjective, parent-reported outcomes does not control for the placebo effects, but will more closely reflect the benefits that would accrue in everyday practice with regard to analgesic effects.
- Contamination is possible given the over-the-counter availability of analgesic eardrops.

For peer review only

For peer review only - http://bmjopen.bmj.com/site/about/guidelines.xhtml

INTRODUCTION

Although the current COVID-19 pandemic has a profound influence on the incidence of acute otitis media (AOM) in children[1-3], it will remain an important cause of primary care consultations.[4 5] Ear pain is the predominant symptom and central to children's and parents' experience of the condition.[6 7] High-quality studies have shown that most symptoms of AOM settle within a few days without antibiotics in otherwise healthy children living in high-income countries[8], but it may take eight days for ear pain to fully resolve in 90% of the children.[9] Globally, most AOM clinical practice guidelines therefore recommend oral analgesics in all, and antibiotics in selected, children.[10] However, antibiotics are frequently prescribed in children with AOM[4 5] leading to side effects in at least 1 out of 10 children and emergence of antimicrobial resistance.[11 12] Evidence of effectiveness of management options to relieve the most distressing symptom of AOM and reduce reliance on antibiotics is therefore urgently needed.[13]

Recently, we found that a general practitioner (GP)-targeted educational intervention to improve pain management in children with AOM resulted in an increase in oral analgesic use, in particular ibuprofen, but failed to reduce parent-reported ear pain and antibiotic use.[14] Therefore, alternative interventions need further exploration. Analgesic ear drops, widely available, cheap and safe, may be well-suited to fill this gap. When applied into the external auditory canal, these agents could relieve pain and distress by blocking nerve conduction of the tympanic membrane.[15] So far, evidence on their use in children with AOM is limited.[15 16] Our trial aims to produce this important evidence.

For peer review only - http://bmjopen.bmj.com/site/about/guidelines.xhtml

OBJECTIVE

The primary aim of this pragmatic randomised controlled trial is to investigate whether analgesic ear drops added to usual care (oral analgesics with/without antibiotics) provide superior ear pain relief over usual care alone in children presenting to primary care with AOM and ear pain.

For peer review only

For peer review only - http://bmjopen.bmj.com/site/about/guidelines.xhtml

METHODS AND ANALYSIS

Study design and setting

A pragmatic, two arm, individually randomised, open, superiority trial with cost-effectiveness analysis in general practices in the Netherlands with a follow-up of 4 weeks will be performed. Potential trial participants will be recruited from primary care (approximately from 150 GPs) in the region of Utrecht, The Netherlands.

A mixed-method process evaluation will be nested within the trial (see Supplemental Material 1).[17-22] This process evaluation will capture data to understand how participating in the trial has an impact on the consultation and behaviour and how AOM interventions (including analgesic ear drops, antibiotics and oral analgesics) are viewed by parents and GPs. The process evaluation consists of a survey with brief statements measuring attitudes of all parents based on the extended Common Sense Model[17] and a three-item Consultation Satisfaction Questionnaire,[18] and a qualitative part (semi-structured interviews capturing parents and GP's views and experiences, respectively).

Participants

Children aged 1-6 years presenting to primary care with AOM[23] and parent-reported ear pain in 24 hours prior to enrolment will be eligible for trial participation. Children with (suspected) tympanic membrane perforation or ventilation tube (as they carry a risk of inner ear damage causing hearing loss or tinnitus after application of lidocaine drops), ear wax obscuring visualisation of the tympanic membrane or those who are very unwell or require hospitalisation will be excluded. Detailed inclusion

For peer review only - http://bmjopen.bmj.com/site/about/guidelines.xhtml

and exclusion criteria are listed in Box 1.

Inclusion criteria:

- Age 1 to 6 years
- Parent-reported ear pain in 24 hours prior to enrolment
- GP-diagnosis of (uni- or bilateral) AOM

Exclusion criteria:

Children:

- with (suspected) tympanic membrane perforation or ventilation tubes
- with ear wax obscuring visualisation of the tympanic membrane
- who are systemically very unwell or require hospital admission (e.g. child has signs

and symptoms of serious illness and/or complications such as mastoiditis/meningitis)

- who are at high risk of serious complications including children with known immunodeficiency other than partial IgA or IgG2 deficiencies, craniofacial malformation including cleft palate, Down syndrome and previous ear surgery (with the exception of ventilation tubes in the past)

Box 1. Full list of inclusion and exclusion criteria

view only

Study procedures and data collection

Recruitment and inclusion of participants

Figure 1 depicts a flow diagram of the study procedures. During regular primary care consultations, participating GPs will inform parents of potentially eligible children about the trial. Next, GPs will ask parents who are interested in participation for consent to provide their contact details to the study team at the University Medical Center (UMC) Utrecht. Members of the team will contact the parents by phone the same day to provide detailed information about the trial. If parents provisionally agree to participate and if the child fulfils the trial eligibility criteria, the study physician will visit the child at home. At this home visit, written informed consent for participation in the trial and the semi-structured interview will be obtained (Supplemental Material 2), baseline demographic and disease-specific data will be collected and a physical examination including otoscopy will be performed. Parents are also asked to complete a generic and disease-specific quality of life questionnaires on behalf of their child.

For peer review only - http://bmjopen.bmj.com/site/about/guidelines.xhtml

A full description of the nested process evaluation is given in Supplemental Material 1

Study group assignment

At the conclusion of the home visit, a trial randomisation website will be accessed for concealed study-group assignment. An independent data manager generates a computer-generated randomisation sequence stratified for age (<2 years vs 2 years and above), AOM laterality (unilateral vs bilateral), and AOM baseline antibiotic prescribing (yes vs no). Children will be randomly allocated to either 1) lidocaine hydrochloride 5mg/g (Otalgan) ear drops 1-2 drops up to six times daily for a maximum of 7 days[24 25] with usual care (oral analgesics, with/without antibiotics) or 2) usual care. Analgesic eardrops will be provided by the study team. These drops should not be used in children with a tympanic membrane perforation or ventilation tube as they carry a risk of inner ear damage causing hearing loss or tinnitus. These children will therefore be excluded, as well as those with ear wax obscuring visualisation of the tympanic membrane. Parents of children allocated to the intervention group are expressly instructed to stop applying the drops if their child develops ear discharge.

The study team will notify the GP about the result of the randomisation. The local pharmacist will be notified in case the child is allocated to the analgesic ear drops, in order to check any known sensitivities/allergies and any interactions (with current or future drugs). Any treatment decisions other than the use of analgesic ear drops, i.e. antibiotics and oral analgesics, will be left to the GP's discretion in both groups. To those allocated to the intervention group, the study physician will not

For peer review only - http://bmjopen.bmj.com/site/about/guidelines.xhtml

provide any treatment advice other than instructions about the use of analgesic ear drops. To those allocated to the control group, the study physician will not provide any treatment advice.

During follow-up parents and GPs will be encouraged to manage AOM symptoms according to current clinical practice guidance on AOM in children issued by the Dutch College of General Practitioners. Lidocaine eardrops are currently not recommended in this guideline due to the lack of evidence.[23]

Follow-up data collection

Participants will be followed for 4 weeks. A telephone call will be scheduled at day 3 which will allow us to capture critical data on our primary outcome. Parents will keep a diary of AOM-related symptoms including ear pain scores and fever recordings, treatment adherence, prescribed and over-the-counter (OTC) medication use, AOM-related health care resource use, child care and travel costs, adverse events and complications of AOM for 4 weeks. At the end of follow-up, parents will complete a questionnaire on parental productivity losses and generic and disease-specific quality of life questionnaires. The diary and questionnaires can either be completed online or on paper forms (see data management). The study team will contact the parents by phone to check if all data is collected. During this phone call, an appointment for the semi-structured interview can be made with parents who are still willing to participate in the nested qualitative investigation.

Validated questionnaires used in the trial

- Child's ear pain intensity will be reported by parents in the symptom diary using a 0-10 numerical

For peer review only - http://bmjopen.bmj.com/site/about/guidelines.xhtml

rating scale which has been validated for measuring pain in children with AOM.[26]

- Child's symptoms of crying/distress, disturbed sleep, interference with normal activity, appetite, fever and hearing problems will be reported by parents in the symptom diary using a 0-6 Likert scale with higher scores indicating greater severity. This scale has been proven valid and was successfully used in our previous trials.[16 26]

- Generic quality of life of the child at baseline and 4 weeks will be assessed using the 47-item short-form of the Infant Toddler Quality of Life Questionnaire (ITQOL-SF47) which is developed for use in infants and toddlers aged 2 months to 6 years and has shown to be a feasible instrument with adequate psychometric properties to discriminate between children with and without doctor-diagnosed respiratory disease.[27]

- Disease-specific quality of life of the child at baseline and 4 weeks will be assessed using the Otitis media-6 (OM-6) questionnaire.[28]

- Productivity losses (e.g. parental absenteeism from work) because of AOM of their child will be assessed using the IMTA Productivity Cost Questionnaire (iPCQ) at 4 weeks.[29]

Outcomes

Primary outcome

The parent-reported ear pain score over the first three days. To this end, parents will record their child's ear pain scores during the first three consecutive days using a 0-10 validated numerical rating scale.

For peer review only - http://bmjopen.bmj.com/site/about/guidelines.xhtml

For peer review only

Secondary outcomes

- proportion of children consuming antibiotics in the first 7 days;
- oral analgesic use in the first 7 days;
- overall symptom burden (crying/distress, disturbed sleep, interference with normal activity, appetite, fever and hearing problems) using a 0-6 Likert scale in the first 7 days;
- number of days with ear pain during follow up (4 weeks);
- number of GP reconsultations with/without subsequent antibiotic prescribing during follow-up;
- adverse events during follow-up;
- complications of AOM during follow-up;
- costs and cost-effectiveness during follow-up;
- generic quality of life of the child at 4 weeks;
- disease-specific quality of life of the child at 4 weeks;
- parents' and GPs' views of treatment acceptability, usability and satisfaction.

Sample size calculation

The main aim is to demonstrate that analgesic ear drops as added to usual care provide superior parent-reported ear pain relief over the first three days compared to usual care. In our recent trial, the mean parent-reported ear pain score (0-10 scale) over the first three days was 4.36 (SD 2.12) among

For peer review only - http://bmjopen.bmj.com/site/about/guidelines.xhtml

those allocated to usual care.[14] Patient and Public Involvement (PPI) input from the UK suggested that a 1-point reduction (on a 0-10 scale) would be a clinically important difference.[16] However, our parent panel felt that even a somewhat smaller difference would be clinically meaningful given the low cost, low risk nature of the intervention. To detect a pain reduction of 20% (reflecting a 0.87 point reduction in pain) compared with usual care with 90% power (beta 0.1) and at a 5% significance level (alpha 0.05), 126 children per group will be needed. To allow for 20% attrition, we aim to include 300 participants.

The estimated sample size will also provide substantial power to address the key secondary outcome of the proportion of children consuming antibiotics. Anticipating 47% of children consuming antibiotics in the usual care group as with our previous trial[14], 252 children will provide 89% power (beta 0.11; alpha 0.05) to detect a 20% difference in the proportion of children consuming antibiotics.

Data management

During study conduct, we will comply with the EU General Data Protection Regulation and the Dutch Act on Implementation of the General Data Protection Regulation. The data management department of the Julius Center, UMC Utrecht will be responsible for handling and storage of the trial data using a secured and coded online database (Research Online 2.0 [RO 2.0]). Data will be either entered directly into RO 2.0 or collected on paper forms first. For data entered directly into RO 2.0, processes to promote data quality are added. Coded trial data will be securely stored for 25 years in the main

For peer review only

study folder on a central drive of the Julius Center.

Patient and public involvement

A panel of eight parents was established for this trial. This panel helped to shape the grant proposal and final study protocol during online focus group meetings, with particular advice given on patient-relevant outcomes including the minimally important clinical differences, recruitment strategy, and the design of data collection. The parent panel will be actively involved throughout all critical stages of the trial through regular parent panel meetings.

Statistical analysis

All analyses will be performed according to the intention-to-treat (ITT) principle and blinded with respect to study-group assignment. The results will be reported in accordance with the CONSORT guidelines.[30]

Clinical effectiveness

Descriptive statistics will be used to assess any baseline differences between treatment groups. The primary outcome will be analysed with a linear regression model with a residual covariance (i.e. GEE type) matrix to correct for repeated measurements. In main (adjusted) analysis, treatment group, time, a priori specified prognostic factors (baseline ear pain score, duration of ear pain prior to enrolment) and stratification factors (age (<2 years vs 2 years and above), AOM laterality (unilateral vs bilateral),

For peer review only - http://bmjopen.bmj.com/site/about/guidelines.xhtml

and baseline antibiotic prescribing (yes vs no)) will be included in the model. The validity of the model, i.e. normality and homoscedasticity, will be evaluated by assessing residuals and the comparison between treatment groups will be expressed as differences in means with 95% confidence intervals (CIs). If more than 5% of baseline and outcome data is missing, a sensitivity analysis will be performed using multiple imputation techniques.[31 32]

Secondary outcomes will be analysed using negative-binomial regression analyses for count variables, log binomial regression analyses for dichotomous variables, and linear regression analyses for continuous variables, where applicable adjusted for repeated measurements. For these analyses, the comparison between treatment groups will be expressed as rate ratios, risk ratios, and mean differences, respectively; all with 95% CIs.

We will also conduct pre-specified, exploratory subgroup analyses to investigate whether age (<2 years vs 2 years and above) or AOM laterality (unilateral vs bilateral) modify the effectiveness of analgesic ear drops. In further sensitivity analysis, we will also explore the added impact of antibiotic consumption on the primary outcome of interest.

Cost effectiveness analysis

Alongside the trial, a cost effectiveness analysis from a societal perspective, i.e. including both medical and non-medical costs, will be performed. The societal perspective is particularly relevant in this case, since non-medical costs are particularly relevant in AOM.[33] Those costs mainly relate to parental productivity losses and costs of additional childcare.

For peer review only

Costs analysis

All costs will be estimated at the patient level. Costs of medication use will be retrieved from the Dutch formulary and a pharmacist's fee will be added for every prescription.[24 34] Costs of OTC and complementary medicines will be calculated per day, based on current average retail prices. Costs of consulting a GP or a medical specialist, and costs of hospitalisations will be based on current Dutch guidelines for pharmaco-economic evaluation in health care research. Costs of diagnostic tests will also be derived from these guidelines.[34 35] Costs of surgical procedures will be retrieved from a previous Dutch costing study that calculated costs for the different components of surgical procedures.[36]

Resource use such as doctor's visits, prescribed medication (including antibiotics), specialist referrals, hospital admissions, surgical interventions as well as out-of-pocket expenses such as OTC medication, child care, and travel costs will be collected from study diaries filled out by parents. Cost prices will be estimated according to guidelines for pharmaco-economic evaluation in health care research.[34 35] Costs associated with productivity losses will be retrieved from the iPCQ questionnaire and estimated using the friction cost method.[29 37] The hourly cost estimate for childcare will be derived from the Dutch National Institute for Family Finance Information (NIBUD).[38] Travel expenses will be calculated for healthcare visits following the Dutch guideline for economic evaluation.[35] Mean costs per patient will be compared across the randomisation groups.. As the time horizon is only 4 weeks, discounting does not apply.

For peer review only - http://bmjopen.bmj.com/site/about/guidelines.xhtml

Cost-effectiveness results

Costs from a societal perspective will be compared with the parent-reported ear pain scores over the first three days. We will calculate incremental cost-effectiveness ratios by dividing the estimated differences in costs between groups by the differences in effects observed, i.e. the additional cost per additional 1 point reduction in mean ear pain score over the first three days for the analgesic ear drops group versus the usual care group. Uncertainty will be addressed by means of two-stage non-parametric bootstrap sampling. The effect of any difference in baseline characteristics on cost-effectiveness results will be investigated using net benefit regression methods. In case of missing data, we will use multiple imputation techniques. The final results will be presented using incremental cost-effectiveness planes and cost-effectiveness acceptability curves.

Monitoring and safety

The trial will be monitored regularly by an independent research data monitor of the UMC Utrecht to ensure the quality of the trial execution. Since we do not expect any safety issues and the overall risk of this trial was judged negligible, we have not established a data safety monitoring board and refrain from conducting a safety interim analysis. However, in accordance to section 10, subsection 4, of the WMO, the sponsor will suspend the study if there is sufficient ground that continuation of the study will jeopardise subject health or safety.

For peer review only

ETHICS AND DISSIMINATION

The study will be conducted according to the principles of the Declaration of Helsinki (10th version, October 2013), the ICH GCP E6(R2) guideline, the Dutch Law on medicinal products ('geneesmiddelenwet') and in accordance with the Medical Research Involving Human Subjects Act (WMO). The Medical Research Ethics Committee Utrecht, the Netherlands, has approved the protocol (21-447/G-D).

Research findings will be disseminated through publications in peer reviewed medical journals targeted towards the wider audience of health professionals involved in the care of children and presented at relevant national and international scientific meetings. We will work with our parent panel to help interpret the findings of the trial and harness their resources for dissemination to the lay public. On completion of the trial, data will be stored for a minimum of 25 years at a central data drive at the Julius Center. We will publish the metadata in the DataverseNL repository and will provide a persistent identifier. The data will be available to researchers who provide a methodologically sound proposal to achieve the aims in the approved proposal. Proposals to gain access to the data should be directed to the principle investigator (RPV). Data requestors will need to sign a data sharing agreement.

CURRENT STUDY STATUS

Study enrolment is ongoing with the first participant being included on 6 October 2021.

DISCUSSION

For peer review only - http://bmjopen.bmj.com/site/about/guidelines.xhtml

Up to now, evidence on the use of analgesic ear drops in children with AOM is limited. A 2011 systematic review included five studies of which two (117 children) compared analgesic ear drops to placebo.[15] The authors concluded that these trials provide limited evidence that a single dose of analgesic ear drops is effective in reducing ear pain 30 minutes after administration in older children (age 3 to 19 years) with AOM.[15] Since then, a UK-based trial compared at-home, repeated dosing, of analgesic ear drops with placebo ear drops and usual care. This trial was terminated prematurely due to operational issues.[16] Initial data of 84 children recruited in primary care indicate that ear pain levels were lower in both ear drops groups than in the usual care group. However, these estimated treatment effects are imprecise because of the low sample size target and therefore require confirmation in future studies.

Our trial aims to do so by assessing the clinical and cost effectiveness of analgesic eardrops in children with AOM, as well as views and experiences of parents and GPs through the nested process evaluation. The pragmatic, open design enhances the applicability of the trial findings to daily practice and is most suited to address the key secondary outcome of the impact of the analgesic ear drops on the proportion of children consuming antibiotics in everyday practice.[39] This would be much more difficult to determine realistically in a placebo-blinded study where children in both groups would receive ear drops (either placebo or analgesic drops); such ‘treatment’ may in itself lead to altered parental (health care seeking) behaviour. Additionally, the open design, in which the control group receives usual care, is most suited for ‘real world’ cost-effectiveness analysis. However, the lack of blinding in combination with the subjective, parent-reported outcomes might also lead to bias in the

For peer review only - http://bmjopen.bmj.com/site/about/guidelines.xhtml

assessment of analgesic effects. To minimise this potential bias, the study physician will emphasize during the baseline visit that it is not known whether the drops have any effect in children with AOM. Furthermore, a recent meta-epidemiological study found no evidence for a difference in estimated treatment effect between blinded and non-blinded trials.[40] Although it is still unclear what caused the results of this study, it suggests that blinding is less important than anticipated. Our design does not control for the placebo effects, but will more closely reflect the benefits that would accrue in everyday practice with regard to analgesic effects.

Albeit not recommended in the Dutch AOM clinical practice guideline[23], the OTC availability of lidocaine eardrops may inherently incur the risk of contamination, i.e. parents in the control group buying lidocaine ear drops themselves. We consider this unlikely since only 5% (10/221) of participants did use lidocaine ear drops in our previous trial of a GP-targeted educational intervention to improve pain management in

children with AOM.[14] We will, however, capture data on the use of lidocaine ear drops in all trial participants to monitor potential contamination.

For peer review only

ACKNOWLEDGEMENTS

We gratefully thank our parent panel for their valuable input and all children and parents who participated in our trial thus far.

AUTHOR'S CONTRIBUTIONS

For peer review only - http://bmjopen.bmj.com/site/about/guidelines.xhtml

RPV, AGMS and RAMJD designed the trial. JLHdS, SH and RPV drafted the first version of the protocol paper. All other authors (RAMJD, AGMS, EAMS, GAdW, NPAZ, LY, SA, PL and ADH) provided feedback on this version. All authors (JLHdS, SH, RPV, RAMJD, AGMS, EAMS, GAdW, NPAZ, LY, SA, PL and ADH) approved the final version of the manuscript.

FUNDING STATEMENT

The trial is supported by a grant from the Netherlands Organisation for Health Research and Development (ZonMw– grant number 10060011910003). The funding agency had no role in the design, and will not have any role during execution of the trial, data-analyses, interpretation of the data or decision to submit results. LY is an NIHR Senior Investigator and her research programme is partly supported by NIHR Applied Research Collaboration (ARC)-West, NIHR Health Protection Research Unit (HPRU) for Behavioural Science and Evaluation, and the NIHR Southampton Biomedical Research Centre (BRC).

COMPETING INTERESTS

The authors declare that they have no conflict of interest.

For peer review only

REFERENCES

1. Hullegie S, Schilder AGM, Marchisio P, et al. A Strong Decline in the Incidence of Childhood Otitis Media During the COVID-19 Pandemic in the Netherlands. *Front Cell Infect Microbiol* 2021;11:768377. doi: 10.3389/fcimb.2021.768377 [published Online First: 2021/11/19]
2. Kaur R, Schulz S, Fuji N, et al. COVID-19 Pandemic Impact on Respiratory Infectious Diseases in Primary Care Practice in Children. *Front Pediatr* 2021;9:722483. doi: 10.3389/fped.2021.722483 [published Online First: 2021/10/01]
3. McBride JA, Eickhoff J, Wald ER. Impact of COVID-19 Quarantine and School Cancelation on Other Common Infectious Diseases. *Pediatr Infect Dis J* 2020;39(12):e449-e52. doi: 10.1097/inf.0000000000002883 [published Online First: 2020/10/09]
4. Gulliford M, Latinovic R, Charlton J, et al. Selective decrease in consultations and antibiotic prescribing for acute respiratory tract infections in UK primary care up to 2006. *J Public Health (Oxf)* 2009;31(4):512-20. doi: 10.1093/pubmed/fdp081 [published Online First: 2009/09/08]
5. van den Broek d'Obrenan J, Verheij TJ, Numans ME, et al. Antibiotic use in Dutch primary care: relation between diagnosis, consultation and treatment. *J Antimicrob Chemother* 2014;69(6):1701-7. doi: 10.1093/jac/dku005 [published Online First: 2014/02/11]
6. Barber C, Ille S, Vergison A, et al. Acute otitis media in young children - what do parents say? *Int J Pediatr Otorhinolaryngol* 2014;78(2):300-6. doi: 10.1016/j.ijporl.2013.11.030 [published Online First: 2014/01/01]
7. Hansen MP, Howlett J, Del Mar C, et al. Parents' beliefs and knowledge about the management of acute otitis media: a qualitative study. *BMC Fam Pract* 2015;16:82. doi: 10.1186/s12875-015-0297-7 [published Online First: 2015/07/08]
8. Venekamp RP, Sanders SL, Glasziou PP, et al. Antibiotics for acute otitis media in children. *Cochrane Database Syst Rev* 2015(6):CD000219. doi: 10.1002/14651858.CD000219.pub4 [published Online First: 2015/06/24]
9. Thompson M, Vodicka TA, Blair PS, et al. Duration of symptoms of respiratory tract infections in children: systematic review. *BMJ* 2013;347:f7027. doi: 10.1136/bmj.f7027 [published Online First: 2013/12/18]
10. Ovnat Tamir S, Shemesh S, Oron Y, et al. Acute otitis media guidelines in selected developed and developing countries: uniformity and diversity. *Arch Dis Child* 2017;102(5):450-57. doi: 10.1136/archdischild-2016-310729 [published Online First: 2016/09/08]
11. Costelloe C, Metcalfe C, Lovering A, et al. Effect of antibiotic prescribing in primary care on antimicrobial resistance in individual patients: systematic review and meta-analysis. *Bmj* 2010;340:c2096. doi: 10.1136/bmj.c2096 [published Online First: 2010/05/21]
12. Gillies M, Ranakusuma A, Hoffmann T, et al. Common harms from amoxicillin: a systematic review and meta-analysis of randomized placebo-controlled trials for any indication. *Cmaj* 2015;187(1):E21-e31. doi: 10.1503/cmaj.140848 [published Online First: 2014/11/19]

For peer review only - http://bmjopen.bmj.com/site/about/guidelines.xhtml

13. Pouwels KB, Dolk FCK, Smith DRM, et al. Actual versus 'ideal' antibiotic prescribing for common conditions in English primary care. *J Antimicrob Chemother* 2018;73(suppl_2):19-26. doi: 10.1093/jac/dkx502 [published Online First: 2018/03/01]
14. van Uum RT, Venekamp RP, Zuithoff NP, et al. Improving pain management in childhood acute otitis media in general practice: a cluster randomised controlled trial of a GP-targeted educational intervention. *Br J Gen Pract* 2020;70(699):e684-e95. doi: 10.3399/bjgp20X712589 [published Online First: 2020/08/26]
15. Foxlee R, Johansson A, Wejfalk J, et al. Topical analgesia for acute otitis media. *Cochrane Database Syst Rev* 2006(3):Cd005657. doi: 10.1002/14651858.CD005657.pub2 [published Online First: 2006/07/21]
16. Hay AD, Downing H, Francis NA, et al. Anaesthetic-analgesic ear drops to reduce antibiotic consumption in children with acute otitis media: the CEDAR RCT. *Health Technol Assess* 2019;23(34):1-48. doi: 10.3310/hta23340 [published Online First: 2019/07/16]
17. Horne R. Treatment perceptions and self-regulation. In: Leventhal H, Cameron L, eds. The self-regulation of health and illness behaviour. London: Routledge 2003:138–53.
18. Yardley L, Douglas E, Anthierens S, et al. Evaluation of a web-based intervention to reduce antibiotic prescribing for LRTI in six European countries: quantitative process analysis of the GRACE/INTRO randomised controlled trial. *Implement Sci* 2013;8:134. doi: 10.1186/1748-5908-8-134 [published Online First: 2013/11/19]
19. van Uum RT, Sjoukes A, Venekamp RP, et al. Pain management in acute otitis media: a qualitative study exploring GPs' views and expectations parallel to a trial of an educational intervention. *BJGP Open* 2018;2(4):bjgpopen18X101620. doi: 10.3399/bjgpopen18X101620 [published Online First: 2019/02/07]
20. van Uum RT, Venekamp RP, Schilder AGM, et al. Pain management in acute otitis media: a qualitative study of parents' views and expectations. *BMC Fam Pract* 2019;20(1):18. doi: 10.1186/s12875-019-0908-9 [published Online First: 2019/01/25]
21. Braun V, Clarke V. What can "thematic analysis" offer health and wellbeing researchers? *Int J Qual Stud Health Well-being* 2014;9:26152. doi: 10.3402/qhw.v9.26152 [published Online First: 2014/10/19]
22. Gale NK, Heath G, Cameron E, et al. Using the framework method for the analysis of qualitative data in multi-disciplinary health research. *BMC Med Res Methodol* 2013;13:117. doi: 10.1186/1471-2288-13-117 [published Online First: 2013/09/21]
23. Damoiseaux RAMJ, Venekamp RP, Eekhof J. NHG-standaard OMA bij kinderen (derde herziening). *Huisarts Wet* 2014;57:648.
24. Farmacotherapeutisch Kompas [Available from: https://www.farmacotherapeutischkompas.nl/.
25. Dutch Children's Formulary 'Kinderformularium' [Available from: https://www.kinderformularium.nl/.
26. Little P, Gould C, Williamson I, et al. Pragmatic randomised controlled trial of two prescribing strategies for childhood acute otitis media. *Bmj* 2001;322(7282):336-42. doi: 10.1136/bmj.322.7282.336 [published Online First: 2001/02/13]

For peer review only

27. Raat H, Landgraf JM, Oostenbrink R, et al. Reliability and validity of the Infant and Toddler Quality of Life Questionnaire (ITQOL) in a general population and respiratory disease sample. *Qual Life Res* 2007;16(3):445-60. doi: 10.1007/s11136-006-9134-8 [published Online First: 2006/11/18]

28. Rosenfeld RM, Goldsmith AJ, Tetlus L, et al. Quality of life for children with otitis media. *Arch Otolaryngol Head Neck Surg* 1997;123(10):1049-54. doi: 10.1001/archotol.1997.01900100019002 [published Online First: 1997/10/27]

29. Bouwmans C, Krol M, Brouwer W, et al. IMTA Productivity Cost Questionnaire (IPCQ). *Value Health* 2014;17(7):A550. doi: 10.1016/j.jval.2014.08.1791 [published Online First: 2014/11/01]

30. Schulz KF, Altman DG, Moher D. CONSORT 2010 statement: updated guidelines for reporting parallel group randomised trials. *Bmj* 2010;340:c332. doi: 10.1136/bmj.c332 [published Online First: 2010/03/25]

31. Donders AR, van der Heijden GJ, Stijnen T, et al. Review: a gentle introduction to imputation of missing values. *J Clin Epidemiol* 2006;59(10):1087-91. doi: 10.1016/j.jclinepi.2006.01.014 [published Online First: 2006/09/19]

32. Rubin D. Multiple imputation for non-response in surveys. New York: John Wiley 1987.

33. Wolleswinkel-van den Bosch JH, Stolk EA, Francois M, et al. The health care burden and societal impact of acute otitis media in seven European countries: results of an Internet survey. *Vaccine* 2010;28 Suppl 6:G39-52. doi: 10.1016/j.vaccine.2010.06.014 [published Online First: 2010/11/16]

34. Zorginstituut Nederland. Kostenhandleiding: Methodologie van kostenonderzoek en referentieprijzen voor economische evaluaties in de gezondheidszorg. 2016 ed. Diemen: Zorginstituut Nederland 2016.

35. Zorginstituut Nederland. Richtlijn voor het uitvoeren van economische evaluaties in de gezondheidszorg. Diemen: Zorginstituut Nederland 2016.

36. Buskens E, van Staaij B, van den Akker J, et al. Adenotonsillectomy or watchful waiting in patients with mild to moderate symptoms of throat infections or adenotonsillar hypertrophy: a randomized comparison of costs and effects. *Arch Otolaryngol Head Neck Surg* 2007;133(11):1083-8. doi: 10.1001/archotol.133.11.1083 [published Online First: 2007/11/21]

37. Koopmanschap MA, Rutten FF, van Ineveld BM, et al. The friction cost method for measuring indirect costs of disease. *J Health Econ* 1995;14(2):171-89. doi: 10.1016/0167-6296(94)00044-5 [published Online First: 1995/05/08]

38. National Institute for Family Finance Information website [Available from: https://www.nibud.nl/consumenten/kosten-van-kinderopvang/.

39. Treweek S, Zwarenstein M. Making trials matter: pragmatic and explanatory trials and the problem of applicability. *Trials* 2009;10:37. doi: 10.1186/1745-6215-10-37 [published Online First: 2009/06/06]

40. Moustgaard H, Clayton GL, Jones HE, et al. Impact of blinding on estimated treatment effects in randomised clinical trials: meta-epidemiological study. *Bmj* 2020;368:l6802. doi: 10.1136/bmj.l6802 [published Online First: 2020/01/23]

For peer review only - http://bmjopen.bmj.com/site/about/guidelines.xhtml

For peer review only

For peer review only - http://bmjopen.bmj.com/site/about/guidelines.xhtml

Figure 1. Flow diagram of study procedures

Legend: *AOM: acute otitis media, GP: general practitioner, OPTIMA: trial acronym*

*Parents who gave consent for the semi-structured interview will be recruited from both the intervention and control arm and will be purposively sampled after follow-up.

Supplemental material 1. Methods and analyses mixed-method process evaluation

Supplemental material 2. Informed consent form

For peer review only

For peer review only - http://bmjopen.bmj.com/site/about/guidelines.xhtml

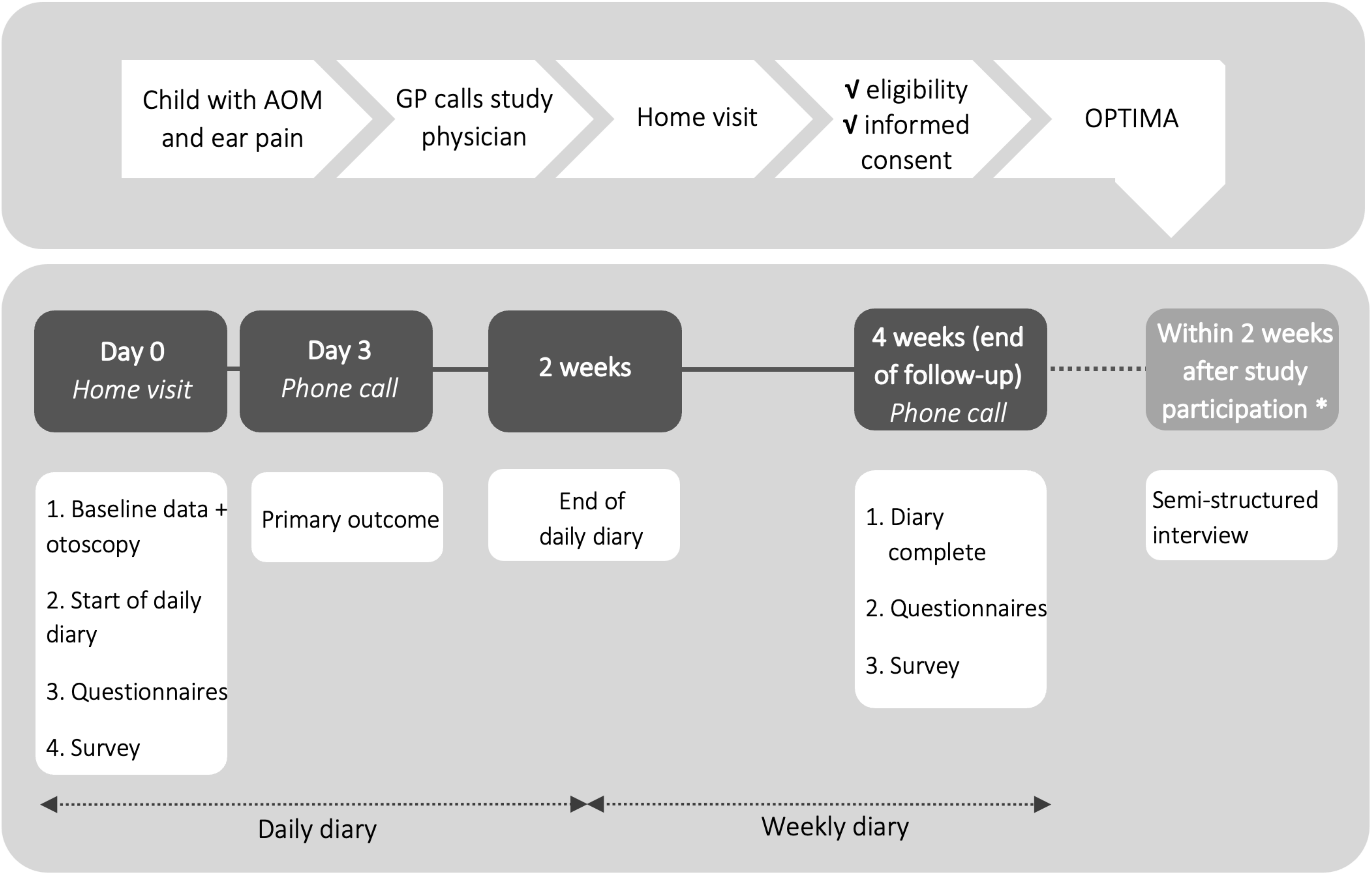
Child with AOM and ear pain
GP calls study physician
Home visit
√ eligibility
√ informed consent
OPTIMA
Day 0
Home visit
Day 3
Phone call
2 weeks
4 weeks (end of follow-up)
Phone call
Within 2 weeks after study participation *
1. Baseline data + otoscopy
2. Start of daily diary
3. Questionnaires
4. Survey
Primary outcome
End of daily diary
1. Diary complete
2. Questionnaires
3. Survey
Semi-structured interview
Daily diary
Weekly diary

Supplemental material 1. Methods and analyses mixed-method process evaluation

Surveys

Surveys will include brief statements which will be rated by parents on a 5-point scale (strongly disagree/ strongly agree). Parents of all trial participants will complete this survey at baseline and at 4 weeks (end of follow-up). Beliefs about the necessity and harms of antibiotics for AOM, oral analgesics and analgesic eardrops were assessed using questions based on the two key dimensions of treatment perceptions in the extended Common Sense Model: 'It is usually necessary to take antibiotics/oral analgesics/analgesic eardrops for an ear infection' and 'Taking antibiotics/oral analgesics/analgesic eardrops for an ear infection can do more harm than good'.[17] Patient satisfaction with the consultation will be assessed by a three-item Consultation Satisfaction Questionnaire that assessed whether they felt they had received all the information and advice they needed and were generally satisfied with the consultation.[18]

Semi-structured interviews

Semi-structured interviews will be carried out with parents and GPs participating in the trial. Interviews with parents will capture experiences of and satisfaction with care and subsequent views and experiences of management of symptoms (including eardrops and antibiotic use). Parents will be recruited from both the intervention and control arm and will be purposively sampled after study participation to obtain variation in age of child, symptom presentation, antibiotic prescribed, eardrops prescribed, gender, number of children, education level and employment status. Interviews will be carried out preferably within 2 weeks after study participation. Interviews with GPs will capture views and experiences of AOM consultations and the use of eardrops in these consultations. GPs will be purposively sampled to obtain variation in practice setting, gender, age and experience. Interviews with GPs will be carried out after the inclusion period. Data will be collected until saturation is reached; based on previous experience, we expect to conduct approximately 10 to 20 semi-structured interviews with both parents and GPs to reach saturation.[19 20]

Each interview will be audio recorded with the participant's permission. Recordings will allow verbatim transcription of interviews in Microsoft Word. Once transcripts are checked, recordings will be deleted. Transcripts will be labelled with a unique participant number and will omit any identifiable data either identifying the participant or their general practice.

Analysis of qualitative data

Qualitative data collection by means of semi-structured interviews and qualitative data analysis will be done concurrently. During the process, multiple members of the study team will review the transcripts of earlier interviews for accuracy and decide whether the topic lists need adaptation.

Interviews with parents and GPs, will be analysed using thematic and framework analysis taking an inductive approach.[21 22] NVivo software will be used to assist with the organisation of data. A thematic framework will be used to chart data across all interviews and will aid comparisons between participants.

For peer review only

Proefpersoneninformatie en toestemmingsformulier voor deelname aan medisch-wetenschappelijk onderzoek

OPTIMA: pijnstillende oordruppels bij acute middenoorontsteking

Officiële titel: Effectiviteit van pijnstillende oordruppels als toevoeging aan systemische pijnstillers bij kinderen met otitis media acuta: een pragmatische gerandomiseerde gecontroleerde studie

For peer review only

Inleiding

Geachte heer/mevrouw,

Met deze informatiebrief willen we u vragen of u met uw kind wilt meedoen aan medisch-wetenschappelijk onderzoek. Meedoen is vrijwillig. U krijgt deze brief omdat uw kind een acute middenoorontsteking heeft.

U leest hier om wat voor onderzoek het gaat, wat het voor u betekent, en wat de voordelen en nadelen zijn. Het is veel informatie. Wilt u de informatie doorlezen en beslissen of u wilt meedoen? Als u wilt meedoen, kunt u het formulier invullen dat u vindt in bijlage C.

Stel uw vragen

U kunt uw beslissing nemen met de informatie die u in deze informatiebrief vindt. Daarnaast raden we u aan om dit te doen:

- Stel vragen aan de onderzoeker die u deze informatie geeft.
- Praat met uw partner, familie of vrienden over dit onderzoek.
- Stel vragen aan de onafhankelijk deskundige, Dr. P.C.J.L. Bruijning-Verhagen (zie bijlage A voor contactgegevens).
- Lees de informatie op www.rijksoverheid.nl/mensenonderzoek.

1. Algemene informatie

Het Universitair Medisch Centrum (UMC) Utrecht heeft dit onderzoek opgezet. Voor dit onderzoek zijn 300 patiënten nodig. Dit onderzoek wordt gefinancierd door ZonMw. Deze organisatie financiert gezondheidsonderzoek in opdracht van het Ministerie van Volksgezondheid, Welzijn en Sport (VWS), en de Nederlandse Organisatie voor Wetenschappelijk Onderzoek (NWO).

De medisch-ethische toetsingscommissie Utrecht heeft dit onderzoek goedgekeurd.

For peer review only - http://bmjopen.bmj.com/site/about/guidelines.xhtml

Optima
pijnstillende oordruppels bij otitis media acuta

UMC Utrecht

2. Wat is het doel van het onderzoek?

In dit onderzoek bekijken we wat het effect is van pijnstillende oordruppels bij kinderen met een acute middenoorontsteking, een ontsteking van de holte achter het trommelvlies.

3. Wat is de achtergrond van het onderzoek?

Een acute middenoorontsteking komt vaak voor op de kinderleeftijd. Het wordt veroorzaakt door een virus of een bacterie. Standaardzorg bestaat momenteel voornamelijk uit pijnstillende behandeling (paracetamol en zo nodig ibuprofen) en indien nodig antibiotica. Ondanks de inzet van deze standaardzorg, gaat de aandoening gepaard met oorpijn en ongemak bij kinderen en kan het een aanzienlijke impact op het gehele gezin hebben door slapeloze nachten en school- en werkverzuim. Het is daarom belangrijk dat er gezocht blijft worden naar andere behandelingen die de klachten kunnen verlichten. Eerdere studies lieten zien dat pijnstillende oordruppels mogelijk een gunstig effect hebben, maar het huidige bewijs is van onvoldoende kwaliteit om een zekere uitspraak over het effect te kunnen doen. Daarom willen we in deze studie onderzoeken of de pijnstillende oordruppels een betere verlichting van de oorpijn geven dan de gebruikelijke pijnstillers alleen. We gaan dit onderzoeken door 300 kinderen middels loting toe te wijzen aan 1) pijnstillende oordruppels bovenop de standaardzorg of 2) standaardzorg

4. Hoe verloopt het onderzoek?

Hoelang duurt het onderzoek?

Doet uw kind mee met het onderzoek? Dan duurt dat in totaal 4 weken.

Stap 1: is uw kind geschikt om mee te doen?

We willen eerst weten of uw kind geschikt is om mee te doen. Uw huisarts controleert of uw kind in aanmerking komt voor studiedeelname. Vervolgens geeft uw huisarts – met uw goedkeuring – uw gegevens door aan het onderzoeksteam in het UMC Utrecht en geeft u deze informatiefolder mee. Het onderzoeksteam neemt telefonisch contact met u op en één van de onderzoekers bezoekt u thuis, binnen 24 uur na uw bezoek aan de huisarts.. Aanvullend aan de beoordeling van de huisarts, zal de onderzoeker u enkele vragen stellen als extra controle op geschiktheid voor deelname.

Tijdens dit bezoek zal de onderzoeker u verder informeren over de studie en mogelijke vragen die u heeft beantwoorden. Pas daarna besluit u of u met uw kind wilt deelnemen aan het onderzoek. Indien u besluit om mee te doen vragen we aan beide ouders/voogden om een toestemmingsformulier te ondertekenen. De onderzoeker ondertekent dit formulier ook. Indien één van de ouders/voogden tijdens het eerste huisbezoek niet aanwezig is, zal van de betreffende ouder mondeling toestemming worden gevraagd. Alleen indien deze ook (mondelinge) toestemming geeft voor deelname, kan uw kind deelnemen aan het onderzoek. In het betreffende geval zal een extra proefpersoneninformatie en toestemmingsformulier bij u thuis worden achtergelaten om door de andere ouder te laten ondertekenen. Het getekende formulier zal op een ander moment opgehaald worden. In bijlage D staat

For peer review only - http://bmjopen.bmj.com/site/about/guidelines.xhtml

bovenstaande procedure van informeren en toestemming geven (informed consent) op een schematische manier weergegeven.

Stap 2: de behandeling

Voor dit onderzoek verdelen we de deelnemende kinderen gelijk over 2 groepen:

- **Groep 1 (interventie groep)**. De kinderen in deze groep krijgen pijnstillende oordruppels (Otalgan® maximaal 6 keer/dag 1-2 druppels gedurende 7 dagen) bovenop de standaardzorg bestaande uit gebruikelijke pijnstillers en indien nodig naar inschatting van de huisarts antibiotica (50% van de kinderen).
  Let op: indien uw kind een loopoor krijgt dan moet u **direct stoppen** met het gebruik van de pijnstillende oordruppels in dat oor, neem in dat geval contact op met de onderzoeker.
- **Groep 2 (controle groep)**. De kinderen in deze groep krijgen de standaardzorg (50% van de kinderen).

Loting bepaalt in welke groep uw kind terecht zal komen.

Stap 3: verzamelen van gegevens

- Het **huisbezoek** op de dag van studiedeelname duurt circa één uur. Tijdens dit huisbezoek zal, nadat er toestemming gegeven is, een kort lichamelijk onderzoek worden verricht waarbij in de oren van uw kind gekeken wordt. Daarnaast neemt de onderzoeker de eerste vragenlijsten met u door. Aan het einde van het eerste huisbezoek zal de arts-onderzoeker via internet contact opnemen met het onderzoekscentrum in het UMC Utrecht om te zien wat het resultaat is van de loting die bepaalt welke behandeling uw kind krijgt. Dit geven wij door aan de huisarts en apotheek van uw kind. Indien uw kind in groep 1 ingedeeld wordt, ontvangt u aan het einde van het bezoek de oordruppels.
- We vragen u gedurende 4 weken om dagelijks een **klachtendagboekje** bij te houden. Dit kan online of op papier gedaan worden. U krijgt hier uitleg over tijdens het huisbezoek.
- Na **3 dagen** neemt een medewerker van het onderzoek **telefonisch contact** met u op om het dagboekje en eventuele vragen te bespreken. Dit duurt circa 10 minuten.
- Na **4 weken** neemt de onderzoeker nogmaals **telefonisch contact** met u op om de studie af te ronden en de laatste vragenlijsten met u door te nemen. Dit telefoon gesprek duurt maximaal 30 minuten.
- Optioneel kunt u ook nog deelnemen aan een telefonisch interview over uw ervaringen met de behandeling van de acute middenoorontsteking en de studie. Dit gebeurt alleen indien u hier toestemming voor geeft.

Wat is er anders dan bij gewone zorg?

Er is bij dit onderzoek niet zoveel anders dan bij gewone zorg. Indien uw kind in groep 1 geloot wordt, krijgt uw kind pijnstillende oordruppels bovenop de gewone zorg. Indien uw kind in groep 2 geplaatst wordt krijgt uw kind de gewone zorg.

For peer review only - http://bmjopen.bmj.com/site/about/guidelines.xhtml

Optima
pijnstillende oordruppels bij otitis media acuta

UMC Utrecht

5. Welke afspraken maken we met u?

We willen graag dat het onderzoek goed verloopt. Daarom maken we de volgende afspraken met u:

- U geeft het medicijn aan uw kind op de manier die de onderzoeker u heeft uitgelegd.
- Uw kind doet tijdens dit onderzoek niet ook nog mee aan een ander medisch-wetenschappelijk onderzoek.
- U vult de vragenlijsten en het dagboekje in zoals met u besproken is.
- U draagt de deelnemerskaart van het onderzoek bij u. Bijvoorbeeld in uw portemonnee. Hierop staat dat uw kind meedoet aan dit onderzoek. En wie men moet waarschuwen bij een noodsituatie. Laat deze kaart zien als uw kind bij een (andere) arts komt.
- Uw huisarts blijft ten alle tijden verantwoordelijk voor de medische behandeling van uw kind. Bij eventuele gezondheidsproblemen van uw kind neemt u contact op met uw huisarts zoals u normaal gesproken ook zou doen.
- U neemt contact op met de onderzoeker in deze specifieke situaties:
    - Uw kind krijgt een **loopoor**. In dit geval moet u **direct stoppen** met het gebruik van de pijnstillende oordruppels in dat oor.
    - Uw kind wordt in een ziekenhuis opgenomen of behandeld.
    - U en/of uw kind wilt niet meer meedoen met het onderzoek.
    - Uw telefoonnummer, adres of e-mailadres verandert.

6. Van welke bijwerkingen, nadelige effecten of ongemakken kunt u last krijgen?

Alle werkzame medicijnen kunnen bijwerkingen hebben. In de bijsluiter van de oordruppels staat dat er een kleine kans is op een overgevoeligheidsreactie. Deze bijwerking komt zelden voor (bij hooguit 1 op de 1.000 personen). We adviseren u dan om direct contact op te nemen met een (huis)arts.
In de bijsluiter van de oordruppels staat verder vermeld dat deze medicatie niet gebruikt dient te worden bij een loopoor of perforatie (gaatje/scheurtje) van het trommelvlies. Uw kind kan niet deelnemen aan het onderzoek als dit het geval is. Zowel de huisarts als de onderzoeker controleert dit aan het begin van het onderzoek door in de oren van uw kind te kijken.

Indien uw kind tijdens het onderzoek een **loopoor** krijgt mag uw kind de oordruppels **niet meer gebruiken**. Neem in dat geval contact op met de onderzoeker.

Doet u mee aan het onderzoek? Dan krijgt u de bijsluiter mee bij het middel.

7. Wat zijn de voordelen en de nadelen als u meedoet aan het onderzoek?

Meedoen aan het onderzoek kan voordelen en nadelen hebben. Hieronder zetten we ze op een rij. Denk hier goed over na, en praat erover met anderen.

For peer review only - http://bmjopen.bmj.com/site/about/guidelines.xhtml

Optima
pijnstillende oordruppels bij otitis media acuta

UMC Utrecht

Meedoen aan het onderzoek kan deze voordelen hebben:
De oordruppels kunnen mogelijk de mate van oorpijn van uw kind en het gebruik van antibiotica verminderen, maar zeker is dit niet.

Meedoen aan het onderzoek kan deze nadelen hebben:

- Uw kind kan last krijgen van bijwerkingen of nadelige effecten van de oordruppels, zoals beschreven in paragraaf 6.
- Meedoen aan het onderzoek kost u extra tijd.
- U moet zich houden aan de afspraken die horen bij het onderzoek.

Wilt u niet meedoen?
U beslist zelf of u met uw kind meedoet aan dit onderzoek. Deelname is geheel vrijwillig. Als u besluit niet mee te doen, hoeft u verder niets te doen. U hoeft niets te tekenen. U hoeft ook niet te zeggen waarom u niet wilt meedoen als u dat niet wilt. Uw kind krijgt dan gewoon de begeleiding en behandeling die hij/zij anders ook zou krijgen van uw huisarts.
Indien u wel meedoet, kunt u, of kan uw kind, zich op ieder moment van de studie bedenken en toch stoppen zonder de reden te melden. Dit zal geen gevolgen hebben voor de behandeling van uw kind.

8. Verzet van uw kind

Het kan gebeuren dat uw kind zich op een bepaald moment tijdens het onderzoek verzet (niet meewerkt). Indien u merkt dat dit het geval is, neem dan direct contact op met de onderzoeker. De onderzoeker moet het onderzoek dan direct stoppen. Het is moeilijk om precies te omschrijven wat verzet is. Voor de start van het onderzoek overleggen we met u wat wij zien als verzet.
De onderzoeker zal zich houden aan de Gedragscode 'Verzet van minderjarigen die deelnemen aan medisch-wetenschappelijk onderzoek' die door het NVK is vastgesteld.

9. Wanneer stopt het onderzoek?

De onderzoeker laat het u weten als er nieuwe informatie over het onderzoek komt die belangrijk voor u is. De onderzoeker vraagt u daarna of u blijft meedoen.

In deze situaties stopt voor u het onderzoek:

- Alle onderzoeken volgens het schema zijn voorbij.
- Het einde van het hele onderzoek is bereikt (na 4 weken)
- U of uw kind wilt zelf stoppen met het onderzoek. Dat mag op ieder moment. Meld dit dan meteen bij de onderzoeker. U hoeft er niet bij te vertellen waarom u stopt. Uw kind krijgt dan weer de gewone behandeling voor de middenoorontsteking. De onderzoeker zal u mogelijk nog wel uitnodigen voor een nagesprek.

For peer review only - http://bmjopen.bmj.com/site/about/guidelines.xhtml

- De onderzoeker vindt het beter voor u om te stoppen. De onderzoeker zal u mogelijk nog wel uitnodigen voor een nagesprek.
- Een van de volgende instanties besluit dat het onderzoek moet stoppen:
    - UMC Utrecht
    - de overheid, of
    - de medisch-ethische commissie die het onderzoek beoordeelt.

Wat gebeurt er als u stopt met het onderzoek?
De onderzoekers gebruiken de gegevens die tot het moment van stoppen zijn verzameld.

Het hele onderzoek is afgelopen als alle deelnemers klaar zijn.

10. Wat gebeurt er na het onderzoek?

Krijgt u de resultaten van het onderzoek?
Na afloop van de hele studie, als de gegevens van alle 300 kinderen zijn verzameld en verwerkt, ontvangt u van ons bericht over de belangrijkste uitkomsten van het onderzoek.

11. Wat doen we met de gegevens van uw kind?

Doet u mee met het onderzoek? Dan geeft u ook toestemming om de gegevens van uw kind te verzamelen, gebruiken en bewaren.

Welke gegevens bewaren we?
We bewaren deze gegevens:
- naam, geslacht en geboortedatum van uw kind
- adres, telefoonnummer en emailadres
- gegevens over gezondheid van uw kind
- (medische) gegevens die we tijdens het onderzoek verzamelen

Waarom verzamelen, gebruiken en bewaren we de gegevens van uw kind?
We verzamelen, gebruiken en bewaren de gegevens van uw kind om de vragen van dit onderzoek te kunnen beantwoorden. En om de resultaten te kunnen publiceren.

Hoe beschermen we de privacy van u en uw kind?
Om de privacy te beschermen geven wij de gegevens een code. Op de gegevens zetten we alleen deze code. De sleutel van de code bewaren we op een beveiligde plek in het UMC Utrecht. Als we de gegevens van uw kind verwerken, gebruiken we steeds alleen die code. Ook in rapporten en publicaties over het onderzoek kan niemand terughalen dat het over uw kind ging.

Wie kunnen de gegevens van uw kind zien?

For peer review only - http://bmjopen.bmj.com/site/about/guidelines.xhtml

Sommige personen kunnen wel de naam en andere persoonlijke gegevens van uw kind zonder code inzien. Dit zijn mensen die controleren of de onderzoekers het onderzoek goed en betrouwbaar uitvoeren. Deze personen kunnen bij de gegevens van uw kind komen:

- Een controleur die voor het UMC Utrecht werkt.
- Nationale toezichthoudende autoriteiten. Bijvoorbeeld de Inspectie Gezondheidszorg en Jeugd.

Deze personen houden de gegevens van uw kind geheim. Wij vragen u voor deze inzage toestemming te geven.

Hoelang bewaren we de gegevens van uw kind?
We bewaren de gegevens van uw kind 25 jaar.

Mogen we de gegevens van uw kind gebruiken voor ander onderzoek?
De gegevens van uw kind kunnen na afloop van dit onderzoek ook nog van belang zijn voor ander wetenschappelijk onderzoek op het gebied van acute middenoorontsteking. Daarvoor zullen de gegevens van uw kind 25 jaar worden bewaard in het onderzoekscentrum. In het toestemmingformulier geeft u aan of u dit goed vindt. Geeft u geen toestemming? Dan kunt u nog steeds meedoen met dit onderzoek. Uw kind krijgt dezelfde zorg.

Kunt u uw toestemming voor het gebruik van de gegevens van uw kind weer intrekken?
U kunt uw toestemming voor het gebruik van de gegevens van uw kind op ieder moment intrekken. Dit geldt voor het gebruik in dit onderzoek en voor het gebruik in ander onderzoek. Maar let op: trekt u uw toestemming in, en hebben onderzoekers dan al gegevens verzameld voor een onderzoek? Dan mogen zij deze gegevens nog wel gebruiken.

Mogelijke sturen we de gegevens van uw kind gecodeerd naar landen buiten de Europese Unie
In dit onderzoek kan het zijn dat we de gecodeerde gegevens van uw kind ook naar landen buiten de Europese Unie sturen. In die landen gelden niet de privacyregels van de Europese Unie. Maar de privacy van uw kind zal op een gelijkwaardig niveau worden beschermd.

Wilt u meer weten over de privacy van uw kind?

- Wilt u meer weten over de rechten van uw kind bij de verwerking van persoonsgegevens? Kijk dan op www.autoriteitpersoonsgegevens.nl.
- Heeft u vragen over de rechten van uw kind? Of heeft u een klacht over de verwerking van de persoonsgegevens van uw kind? Neem dan contact op met degene die verantwoordelijk is voor de verwerking van de persoonsgegevens van uw kind. Voor uw onderzoek is dat:
    - Het UMC Utrecht. Zie bijlage A voor contactgegevens, en website.
- Als u klachten heeft over de verwerking van de persoonsgegevens van uw kind, raden we u aan om deze eerst te bespreken met het onderzoeksteam. U kunt ook naar de

For peer review only - http://bmjopen.bmj.com/site/about/guidelines.xhtml

Functionaris Gegevensbescherming van het UMC Utrecht gaan. Of u dient een klacht in bij de Autoriteit Persoonsgegevens.

Waar vindt u meer informatie over het onderzoek?
Op de volgende website vindt u meer informatie over het onderzoek: https://www.trialregister.nl/trial/9500. Na het onderzoek kan de website een samenvatting van de resultaten van dit onderzoek tonen. U vindt het onderzoek door te zoeken op 'NL9500'.

12. Krijgt u een vergoeding als uw kind meedoet aan het onderzoek?

Aan het onderzoek zijn voor u geen kosten verbonden. Er is geen vergoeding beschikbaar voor deelname, na afloop van het huisbezoek krijgt uw kind wel een klein cadeautje.

13. Bent u verzekerd tijdens het onderzoek?

Voor iedereen die meedoet aan dit onderzoek is een verzekering afgesloten. De verzekering betaalt voor schade door het onderzoek. Maar niet voor alle schade. In **bijlage B** vindt u meer informatie over de verzekering en de uitzonderingen. Daar staat ook aan wie u schade kunt melden.

14. We informeren de huisarts en apotheker van uw kind

De onderzoeker stuurt de huisarts en apotheker van uw kind een bericht om te laten weten dat u meedoet aan het onderzoek. Daarnaast vragen we de apotheek naar bekende allergieën. Dit is voor de eigen veiligheid van uw kind.

15. Heeft u vragen?

Vragen over het onderzoek kunt u stellen aan het onderzoeksteam. Wilt u advies van iemand die er geen belang bij heeft? Dan kunt u terecht bij een onafhankelijke deskundige. Voor ons onderzoek is dat dr. P.C.J.L. Bruijning-Verhagen (zie bijlage A voor contactgegevens). Zij weet veel over het onderzoek, maar werkt niet mee aan dit onderzoek.
Heeft u een klacht? Bespreek dit dan met de onderzoeker of de arts die u behandelt. Wilt u dit liever niet? Ga dan naar Klachtenbemiddeling van het UMC Utrecht. In bijlage A staat waar u die kunt vinden.

16. Hoe geeft u toestemming voor het onderzoek?

U kunt eerst rustig nadenken over dit onderzoek. Daarna vertelt u de onderzoeker of u de informatie begrijpt en of u wel of niet wilt meedoen met uw kind. Wilt u met uw kind meedoen? Dan vult u het toestemmingsformulier in dat u bij deze informatiebrief vindt. U en de onderzoeker krijgen allebei een getekende versie van deze toestemmingsverklaring.

Dank voor uw tijd.

For peer review only - http://bmjopen.bmj.com/site/about/guidelines.xhtml

Met vriendelijke groet,

Drs. Joline de Sévaux, arts-onderzoeker, mede namens OPTIMA onderzoeksteam:

Dr. Roderick P Venekamp, huisarts

Professor dr. Roger AMJ Damoiseaux, huisarts

Professor dr. Anne GM Schilder, KNO-arts

Drs. Saskia Hullegie, arts-onderzoeker

17. Bijlagen bij deze informatie

A. Contactgegevens

B. Informatie over de verzekering

C. Toestemmingsformulier

D. Procedure informeren en toestemming geven (informed consent)

For peer review only

For peer review only - http://bmjopen.bmj.com/site/about/guidelines.xhtml

Bijlage A: contactgegevens voor UMC Utrecht

Contactpersoon onderzoeksteam:
Naam: J.L.H. de Sévaux, arts-onderzoeker
Julius Centrum voor Gezondheidswetenschappen en Eerstelijnsgeneeskunde, Afdeling Huisartsgeneeskunde
Universitair Medisch Centrum Utrecht
e-mail: optima@umcutrecht.nl
tel. nummer: 0625710099
Bereikbaar: dagelijks tijdens kantooruren.

Onafhankelijk deskundige:
Naam: Dr. P.C.J.L. Bruijning-Verhagen, kinderarts
UMC Utrecht
email: p.bruijning@umcutrecht.nl
tel. nummer: 088 75 681 81

Klachten:
Als u klachten heeft kunt u dit melden aan de onderzoeker of aan uw behandelend arts. Mocht u ontevreden zijn over de gang van zaken bij het onderzoek en een klacht willen indienen dan kunt u contact opnemen met de klachtenbemiddelaars. Deze zijn bereikbaar via tel. +31 88 755 62 08. Of digitaal via: https://www.umcutrecht.nl/nl/een-klacht-indienen

Functionaris voor de Gegevensbescherming van de instelling: privacy@umcutrecht.nl

Raadpleeg de website van het UMC Utrecht voor meer informatie over uw rechten: https://www.umcutrecht.nl/nl/ziekenhuis/privacy

For peer review only

For peer review only - http://bmjopen.bmj.com/site/about/guidelines.xhtml

pijnstillende oordruppels bij otitis media acuta

UMC Utrecht

Bijlage B: informatie over de verzekering

UMC Utrecht heeft een verzekering afgesloten voor iedereen die meedoet aan het onderzoek. De verzekering betaalt de schade die u heeft doordat u aan het onderzoek meedeed. Het gaat om schade die u krijgt tijdens het onderzoek, of binnen 4 jaar na het onderzoek. U moet schade binnen 4 jaar melden bij de verzekeraar.

Heeft u schade door het onderzoek? Meld dit dan telefonisch of per post bij deze verzekeraar:

De verzekeraar van het onderzoek is:	
Naam:	CNA Insurance Company Ltd
Adres:	Polarisavenue 140, 2132 JX Hoofddorp
Telefoonnummer:	020 57 37 274
Polisnummer:	10201366
Contactpersoon:	Mw. Esther van Herk

De verzekering betaalt maximaal € 650.000 per persoon en € 5.000.000 voor het hele onderzoek en € 7.500.000 per jaar voor alle onderzoeken van dezelfde opdrachtgever (het UMC Utrecht).

Let op: de verzekering dekt de volgende schade **niet**:

- Schade door een risico waarover we u informatie hebben gegeven in deze brief. Maar dit geldt niet als het risico groter bleek te zijn dan we van tevoren dachten. Of als het risico heel onwaarschijnlijk was.
- Schade aan uw gezondheid die ook zou zijn ontstaan als u niet aan het onderzoek had meegedaan.
- Schade die ontstaat doordat u aanwijzingen of instructies niet of niet goed opvolgde.
- Schade aan de gezondheid van uw kinderen of kleinkinderen.
- Schade door een behandelmethode die al bestaat. Of door onderzoek naar een behandelmethode die al bestaat.

Deze bepalingen staan in het 'Besluit verplichte verzekering bij medisch-wetenschappelijk onderzoek met mensen 2015'. Dit besluit staat in de Wettenbank van de overheid (https://wetten.overheid.nl).

For peer review only

For peer review only - http://bmjopen.bmj.com/site/about/guidelines.xhtml

Bijlage C: Toestemmingsformulier ouders of voogd

Behorende bij de **OPTIMA-studie (pijnstillende oordruppels bij acute middenoorontsteking)**

Ik ben gevraagd om toestemming te geven voor deelname van mijn kind aan dit medisch-wetenschappelijke onderzoek:

Naam proefpersoon (kind): …………………….. Geboortedatum: ___ / ___ / _____

- Ik heb de informatiebrief voor de ouders/verzorgers gelezen. Ook kon ik vragen stellen. Mijn vragen zijn goed genoeg beantwoord. Ik had genoeg tijd om te beslissen of ik wil dat mijn kind meedoet.
- Ik weet dat meedoen vrijwillig is. Ook weet ik dat ik op ieder moment kan beslissen dat mijn kind toch niet meedoet. Ik hoef dan niet te zeggen waarom ik dat wil.
- Ik geef de onderzoeker toestemming om mijn huisarts en apotheker te laten weten dat mijn kind meedoet aan dit onderzoek.
- Ik geef de onderzoekers toestemming om de gegevens van mijn kind te verzamelen en te gebruiken. De onderzoekers doen dit alleen om de onderzoeksvraag in dit onderzoek te beantwoorden.
- Ik weet dat voor de controle van het onderzoek sommige mensen toegang tot alle gegevens van mijn kind kunnen krijgen. Die mensen staan in deze informatiebrief. Ik geef deze mensen toestemming om de gegevens van mijn kind in te zien voor deze controle.
- Ik weet dat mijn gecodeerde gegevens naar landen buiten de EU kunnen worden gestuurd waar privacyregels van de EU niet gelden.
- Wilt u in de tabel hieronder ja of nee aankruisen?

Ik geef toestemming om mij na dit onderzoek te benaderen voor een interview over uw ervaringen met betrekking tot de studiedeelname en de behandeling.	Ja ☐	Nee☐
Ik geef toestemming om mijn kind na dit onderzoek te vragen of hij/zij wil meedoen met een vervolgonderzoek.	Ja ☐	Nee☐
Ik geef toestemming om de gegevens van mijn kind te bewaren om dit te gebruiken voor ander onderzoek, zoals in de informatiebrief staat.	Ja ☐	Nee☐

- Ik ga ermee akkoord dat mijn kind meedoet aan dit onderzoek.

For peer review only - http://bmjopen.bmj.com/site/about/guidelines.xhtml

Naam ouder/voogd**: ……………………………….

Handtekening: ………………………………… Datum: ___ / ___ / _____

Naam andere ouder/voogd**: …………………….

Handtekening: ……………………………………… Datum: ___ / ___ / _____

Ik verklaar dat ik de persoon/personen hierboven volledig heb geïnformeerd over het genoemde onderzoek.

Wordt er tijdens het onderzoek informatie bekend die de toestemming van de ouder of voogd kan beïnvloeden? Dan laat ik dit op tijd aan hem/haar weten.

Naam onderzoeker (of diens vertegenwoordiger): …………………………

Handtekening: ……………………… Datum: ___ / ___ / _____

---

* Doorhalen wat niet van toepassing is.

** Als het kind jonger dan 16 jaar is, ondertekenen de ouders die het gezag uitoefenen of de voogd dit formulier. Kinderen van 12 t/m 15 jaar die zelfstandig beslissingen kunnen nemen (wilsbekwaam zijn), moeten daarnaast zelf een formulier ondertekenen

De ouder/voogd krijgt een volledige informatiebrief mee, samen met een getekende versie van het toestemmingsformulier.

For peer review only

For peer review only - http://bmjopen.bmj.com/site/about/guidelines.xhtml

Bijlage D: Procedure informeren en toestemming geven (informed consent)

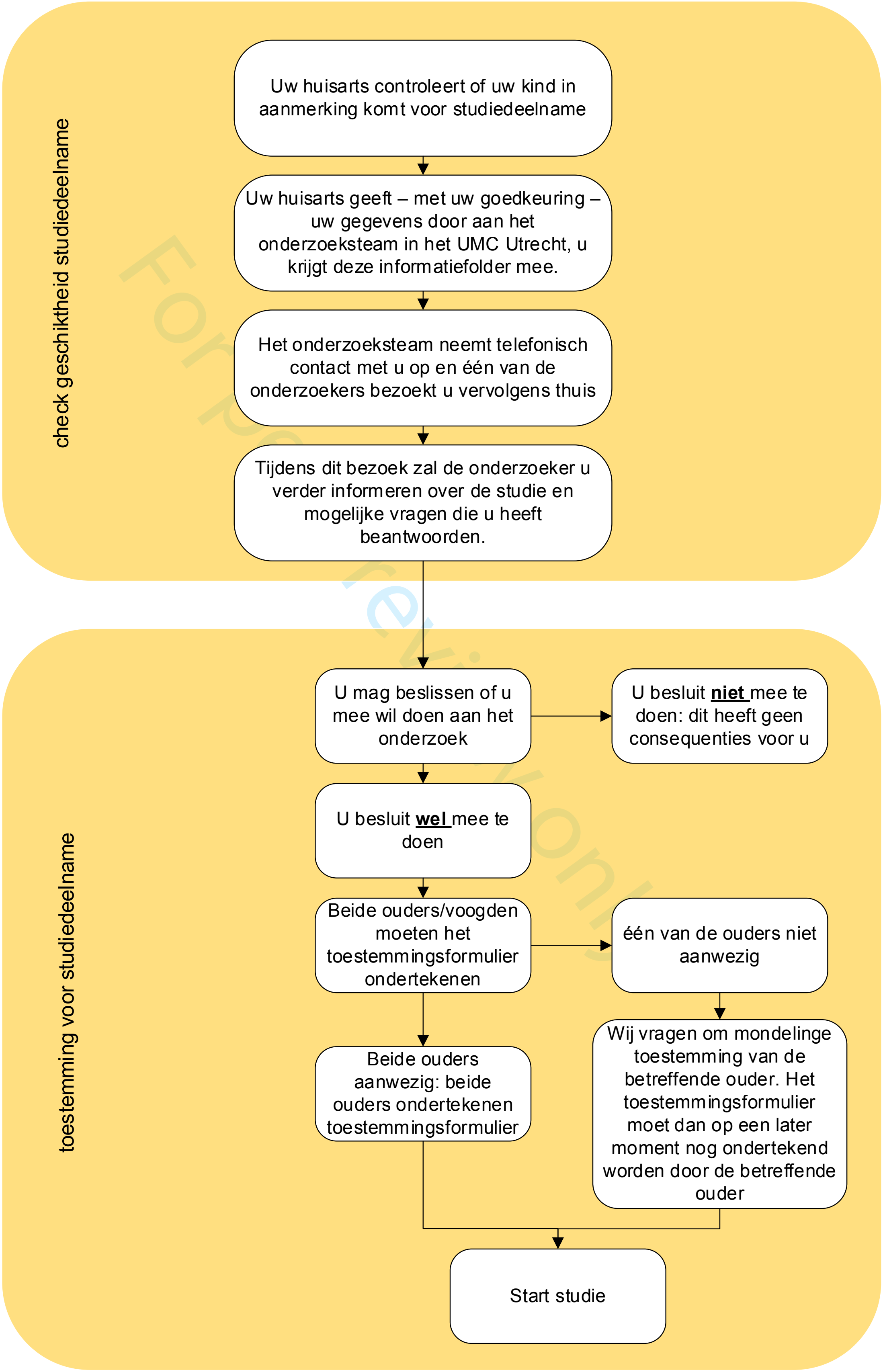

For peer review only - http://bmjopen.bmj.com/site/about/guidelines.xhtml

SPIRIT
STANDARD PROTOCOL ITEMS: RECOMMENDATIONS FOR INTERVENTIONAL TRIALS

SPIRIT 2013 Checklist: Recommended items to address in a clinical trial protocol and related documents*

Section/item	Item No	Description	Page number
**Administrative information**			
Title	1	Descriptive title identifying the study design, population, interventions, and, if applicable, trial acronym	1
Trial registration	2a	Trial identifier and registry name. If not yet registered, name of intended registry	2
	2b	All items from the World Health Organization Trial Registration Data Set	2
Protocol version	3	Date and version identifier	n/a
Funding	4	Sources and types of financial, material, and other support	13
Roles and responsibilities	5a	Names, affiliations, and roles of protocol contributors	1
	5b	Name and contact information for the trial sponsor	n/a
	5c	Role of study sponsor and funders, if any, in study design; collection, management, analysis, and interpretation of data; writing of the report; and the decision to submit the report for publication, including whether they will have ultimate authority over any of these activities	13
	5d	Composition, roles, and responsibilities of the coordinating centre, steering committee, endpoint adjudication committee, data management team, and other individuals or groups overseeing the trial, if applicable (see Item 21a for data monitoring committee)	11
**Introduction**			
Background and rationale	6a	Description of research question and justification for undertaking the trial, including summary of relevant studies (published and unpublished) examining benefits and harms for each intervention	4
	6b	Explanation for choice of comparators	4
Objectives	7	Specific objectives or hypotheses	4

For peer review only

Trial design	8	Description of trial design including type of trial (eg, parallel group, crossover, factorial, single group), allocation ratio, and framework (eg, superiority, equivalence, noninferiority, exploratory)	5
**Methods: Participants, interventions, and outcomes**			
Study setting	9	Description of study settings (eg, community clinic, academic hospital) and list of countries where data will be collected. Reference to where list of study sites can be obtained	5
Eligibility criteria	10	Inclusion and exclusion criteria for participants. If applicable, eligibility criteria for study centres and individuals who will perform the interventions (eg, surgeons, psychotherapists)	5,16 (Box 1)
Interventions	11a	Interventions for each group with sufficient detail to allow replication, including how and when they will be administered	6
	11b	Criteria for discontinuing or modifying allocated interventions for a given trial participant (eg, drug dose change in response to harms, participant request, or improving/worsening disease)	6
	11c	Strategies to improve adherence to intervention protocols, and any procedures for monitoring adherence (eg, drug tablet return, laboratory tests)	6
	11d	Relevant concomitant care and interventions that are permitted or prohibited during the trial	n/a
Outcomes	12	Primary, secondary, and other outcomes, including the specific measurement variable (eg, systolic blood pressure), analysis metric (eg, change from baseline, final value, time to event), method of aggregation (eg, median, proportion), and time point for each outcome. Explanation of the clinical relevance of chosen efficacy and harm outcomes is strongly recommended	7,8
Participant timeline	13	Time schedule of enrolment, interventions (including any run-ins and washouts), assessments, and visits for participants. A schematic diagram is highly recommended (see Figure)	5,6, Figure 1
Sample size	14	Estimated number of participants needed to achieve study objectives and how it was determined, including clinical and statistical assumptions supporting any sample size calculations	8
Recruitment	15	Strategies for achieving adequate participant enrolment to reach target sample size	5
**Methods: Assignment of interventions (for controlled trials)**			
Allocation:			

Sequence generation	16a	Method of generating the allocation sequence (eg, computer-generated random numbers), and list of any factors for stratification. To reduce predictability of a random sequence, details of any planned restriction (eg, blocking) should be provided in a separate document that is unavailable to those who enrol participants or assign interventions	6
Allocation concealment mechanism	16b	Mechanism of implementing the allocation sequence (eg, central telephone; sequentially numbered, opaque, sealed envelopes), describing any steps to conceal the sequence until interventions are assigned	6
Implementation	16c	Who will generate the allocation sequence, who will enrol participants, and who will assign participants to interventions	6
Blinding (masking)	17a	Who will be blinded after assignment to interventions (eg, trial participants, care providers, outcome assessors, data analysts), and how	n/a (open trial)
	17b	If blinded, circumstances under which unblinding is permissible, and procedure for revealing a participant's allocated intervention during the trial	n/a
**Methods: Data collection, management, and analysis**			
Data collection methods	18a	Plans for assessment and collection of outcome, baseline, and other trial data, including any related processes to promote data quality (eg, duplicate measurements, training of assessors) and a description of study instruments (eg, questionnaires, laboratory tests) along with their reliability and validity, if known. Reference to where data collection forms can be found, if not in the protocol	5-7
	18b	Plans to promote participant retention and complete follow-up, including list of any outcome data to be collected for participants who discontinue or deviate from intervention protocols	5,6
Data management	19	Plans for data entry, coding, security, and storage, including any related processes to promote data quality (eg, double data entry; range checks for data values). Reference to where details of data management procedures can be found, if not in the protocol	8
Statistical methods	20a	Statistical methods for analysing primary and secondary outcomes. Reference to where other details of the statistical analysis plan can be found, if not in the protocol	9,10
	20b	Methods for any additional analyses (eg, subgroup and adjusted analyses)	9,10

	20c	Definition of analysis population relating to protocol non-adherence (eg, as randomised analysis), and any statistical methods to handle missing data (eg, multiple imputation)	9,10
**Methods: Monitoring**			
Data monitoring	21a	Composition of data monitoring committee (DMC); summary of its role and reporting structure; statement of whether it is independent from the sponsor and competing interests; and reference to where further details about its charter can be found, if not in the protocol. Alternatively, an explanation of why a DMC is not needed	11
	21b	Description of any interim analyses and stopping guidelines, including who will have access to these interim results and make the final decision to terminate the trial	11
Harms	22	Plans for collecting, assessing, reporting, and managing solicited and spontaneously reported adverse events and other unintended effects of trial interventions or trial conduct	6
Auditing	23	Frequency and procedures for auditing trial conduct, if any, and whether the process will be independent from investigators and the sponsor	n/a
**Ethics and dissemination**			
Research ethics approval	24	Plans for seeking research ethics committee/institutional review board (REC/IRB) approval	11
Protocol amendments	25	Plans for communicating important protocol modifications (eg, changes to eligibility criteria, outcomes, analyses) to relevant parties (eg, investigators, REC/IRBs, trial participants, trial registries, journals, regulators)	n/a
Consent or assent	26a	Who will obtain informed consent or assent from potential trial participants or authorised surrogates, and how (see Item 32)	5, supplemental material 2
	26b	Additional consent provisions for collection and use of participant data and biological specimens in ancillary studies, if applicable	supplemental material 2
Confidentiality	27	How personal information about potential and enrolled participants will be collected, shared, and maintained in order to protect confidentiality before, during, and after the trial	8
Declaration of interests	28	Financial and other competing interests for principal investigators for the overall trial and each study site	13
Access to data	29	Statement of who will have access to the final trial dataset, and disclosure of contractual agreements that limit such access for investigators	8

For peer review only

For peer review only - http://bmjopen.bmj.com/site/about/guidelines.xhtml

Ancillary and post-trial care	30	Provisions, if any, for ancillary and post-trial care, and for compensation to those who suffer harm from trial participation	n/a
Dissemination policy	31a	Plans for investigators and sponsor to communicate trial results to participants, healthcare professionals, the public, and other relevant groups (eg, via publication, reporting in results databases, or other data sharing arrangements), including any publication restrictions	11
	31b	Authorship eligibility guidelines and any intended use of professional writers	n/a
	31c	Plans, if any, for granting public access to the full protocol, participant-level dataset, and statistical code	n/a
**Appendices**			
Informed consent materials	32	Model consent form and other related documentation given to participants and authorised surrogates	Supplemental material 2
Biological specimens	33	Plans for collection, laboratory evaluation, and storage of biological specimens for genetic or molecular analysis in the current trial and for future use in ancillary studies, if applicable	n/a

*It is strongly recommended that this checklist be read in conjunction with the SPIRIT 2013 Explanation & Elaboration for important clarification on the items. Amendments to the protocol should be tracked and dated. The SPIRIT checklist is copyrighted by the SPIRIT Group under the Creative Commons "Attribution-NonCommercial-NoDerivs 3.0 Unported" license.

For peer review only
